# Structure‐Based Design of Fluorogenic Substrates Selective for Human Proteasome Subunits

**DOI:** 10.1002/cbic.202000375

**Published:** 2020-07-29

**Authors:** Elmer Maurits, Christian G. Degeling, Alexei F. Kisselev, Bogdan I. Florea, Herman S. Overkleeft

**Affiliations:** ^1^ Leiden Institute of Chemistry Leiden University Einsteinweg 55 2333 CC Leiden The Netherlands; ^2^ Department of Drug Discovery and Development Harrison School of Pharmacy Auburn University Auburn AL 36849 USA

**Keywords:** fluorogenic substrates, immunoproteasome, kinetics, Michaelis-Menten kinetics, proteasome

## Abstract

Proteasomes are established therapeutic targets for hematological cancers and promising targets for autoimmune diseases. In the past, we have designed and synthesized mechanism‐based proteasome inhibitors that are selective for the individual catalytic activities of human constitutive proteasomes and immunoproteasomes: β1c, β1i, β2c, β2i, β5c and β5i. We show here that by taking the oligopeptide recognition element and substituting the electrophile for a fluorogenic leaving group, fluorogenic substrates are obtained that report on the proteasome catalytic activity also targeted by the parent inhibitor. Though not generally applicable (β5c and β2i substrates showing low activity), effective fluorogenic substrates reporting on the individual activity of β1c, β1i, β2c and β5i subunits in Raji (human B cell) lysates and purified 20S proteasome were identified in this manner. Our work thus adds to the expanding proteasome research toolbox through the identification of new and/or more effective subunit‐selective fluorogenic substrates.

## Graphical abstract

**Selective reporting**: A set of fluorogenic substrates for each of the human proteasome subunits was reverse designed based on selective proteasome inhibitors. Assays on both cell lysates and purified proteasome were conducted and revealed high selectivity could be maintained.

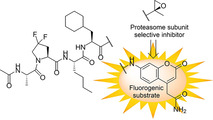

## Introduction

Proteasomes are established clinical targets for the treatment of multiple myeloma and mantle cell lymphoma and are now also considered as therapeutic targets for the treatment of autoimmune diseases.[^1^, ^2^, ^3^] Tools that report on the individual proteolytic activities of human proteasomes are essential for studies on proteasomes and their role in cellular and physiological processes, as well as for the development of effective proteasome inhibitors as candidate‐drugs.[^4^, ^5^] Proteasomes come in different flavors, featuring related yet distinct catalytic activities, and the means to report on these individually is essential to arrive at optimal candidate clinical agents in terms of efficacy and toxicity.^[6]^ All human tissues express constitutive proteasomes core particles (cCP), which harbor three catalytic subunits (two copies of each) known as β1c (cleaving within polypeptides preferably C‐terminal of acidic amino acid residues), β2c (preferring basic residues) and β5c (preferring hydrophobic residues). Some immune‐competent cells express immunoproteasome core particles (iCP), featuring three activities distinct from constitutive proteasomes (termed β1i, β2i and β5i) that might also be induced in other cell types in a cytokine‐stimulated manner.^[7]^ Several hematological cancers in fact express predominantly and in some instances almost exclusively immunoproteasomes. The currently applied proteasome‐targeting clinical drugs (bortezomib, carfilzomib, ixazomib), in contrast, do not discriminate between the active subunits of the two proteasomes and possibly side effects may be prohibited by disabling more specifically proteasome activities that predominate in hematological cancers.^[4]^ This fact underscores the importance of research tools reporting on individual proteasome activities and holds true even more when considering the fact that, besides constitutive proteasomes and immunoproteasomes, also mixed proteasomes featuring both constitutive proteasome and immunoproteasome activities exist.[^8^, ^9^]

Our work on proteasome assays has focused on the development of activity‐based probes, both subunit‐selective and pan‐proteasome‐reactive ones.[^4^, ^10^] Activity‐based probes are mechanism‐based, covalent and irreversible enzyme inhibitors equipped with a reporter entity (normally a fluorophore, biotin or a bioorthogonal group for two‐step activity‐based protein profiling). These probes in turn were derived from their untagged counterparts, themselves of interest in a biomedical context: carfilzomib, the second‐in‐class clinical proteasome inhibitor, is derived from the natural product, epoxomicin, which is a mechanism‐based proteasome inhibitor. Tuning of the oligopeptide recognition element in peptide vinyl sulfones and peptide epoxyketones – the two electrophiles introduced originally by the groups of Ploegh^[11]^ and Crews,^[12]^ respectively, and favored by us – has resulted in a set of six mechanism‐based inhibitors, one selective for each of the individual catalytic activities of human constitutive proteasomes and immunoproteasomes.[^13^, ^14^, ^15^] Having knowledge on oligopeptide sequences able to confer selectivity, we felt it opportune to assess whether selectivity would remain when redesigning the inhibitors into fluorogenic substrates – a strategy that was previously and successfully applied by Turk and Wendt and coworkers, who termed their strategy “reverse design”.^[16]^ This class of reporter entities has in fact been in use in proteasome studies – and indeed in the study of hydrolases in general – for many years, surpassing activity‐based protein profiling strategies.[^17^, ^18^] Yet, to date, only fluorogenic substrates selective for β1i, β1c, β5i and β5c proteasome subunits have been reported, with currently no means to assess β2c and β2i in fluorogenic substrate assay.[^19^, ^20^, ^21^, ^22^, ^23^, ^24^] Besides, selectivity over other subunits and other proteases can sometimes be low for the reported compounds.^[17]^ The research described here and that is based on the above thoughts presents fluorogenic substrates selective for β1c and β1i subunits as additions to the proteasome research tool portfolio. As well, fluorogenic substrates targeting β2c and β5i prove at least equal to the existing ones, whereas selective fluorogenic substrates for β2i and β5c lack significant activity. Our work brings us one step closer to a comprehensive proteasome toolkit comprising inhibitors, activity‐based probes and reporter substrates selective for each of the catalytic activities of constitutive proteasomes and immunoproteasomes alike.

## Results and Discussion

### Synthesis

**Scheme 1:**
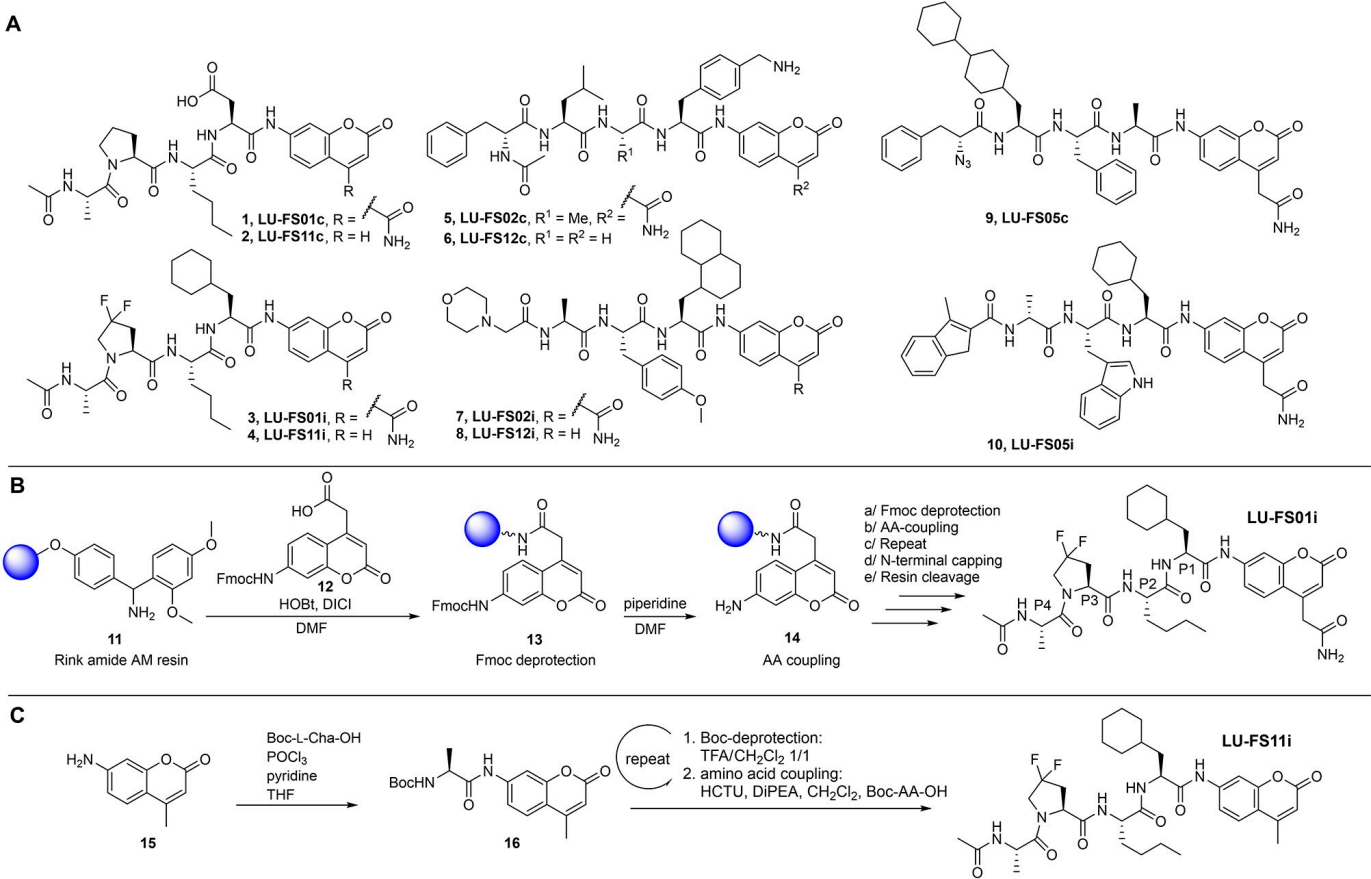
Newly developed proteasome subunit‐specific fluorogenic substrates. A) Chemical structures. B) General solid‐phase synthesis of fluorogenic substrates. C) Synthesis of AMC analogues. The terminology of the fluorogenic substrates is based on the previously published proteasome inhibitors (e. g., LU‐001i), they are abbreviated by LU (Leiden University) – FS (fluorogenic substrate): 0 (ACC) or 1 (AMC) followed by their subunit i (immunoproteasome) or c (constitutive proteasome).

The structures of the fluorogenic substrates and the synthesis schemes we employed for their preparation are depicted in Scheme 1A. At the onset of our studies, we adopted the solid‐phase peptide synthesis strategy (SPPS) developed by Craik and Ellman who employed the RINK linker, which is condensed with Fmoc‐aminocoumarin‐acetic acid (ACC) **12** as the first amino acid employed (Scheme 1B).[^25^, ^26^] Ensuing Fmoc‐SPPS, acid‐mediated cleavage from the resin and HPLC purification – demonstrated here for fluorogenic substrate LU‐FS01i – afforded the six peptide‐aminocoumaryl‐amides LU‐FS01c, LU‐FS01i, LU‐FS02c,LU‐FS02i, LU‐FS05c and LU‐FS05i in good overall yield and purity. This solid phase synthesis procedure works well for the rapid preparation of a variety of substrates, but is less effective when aiming for larger quantities of a desirable fluorogenic substrate. Structurally and functionally (fluorescent properties) close analogues can however be prepared in solution, starting from aminocoumarin (AMC) **15** (Scheme 1C), a strategy that we applied for the construction of LU‐FS11c, LU‐FS11i, LU‐FS12c, LU‐FS12i, LU‐FS15c, LU‐FS25c and LU‐FS35c.

### Substrate hydrolysis in cell extracts

**Figure 1:**
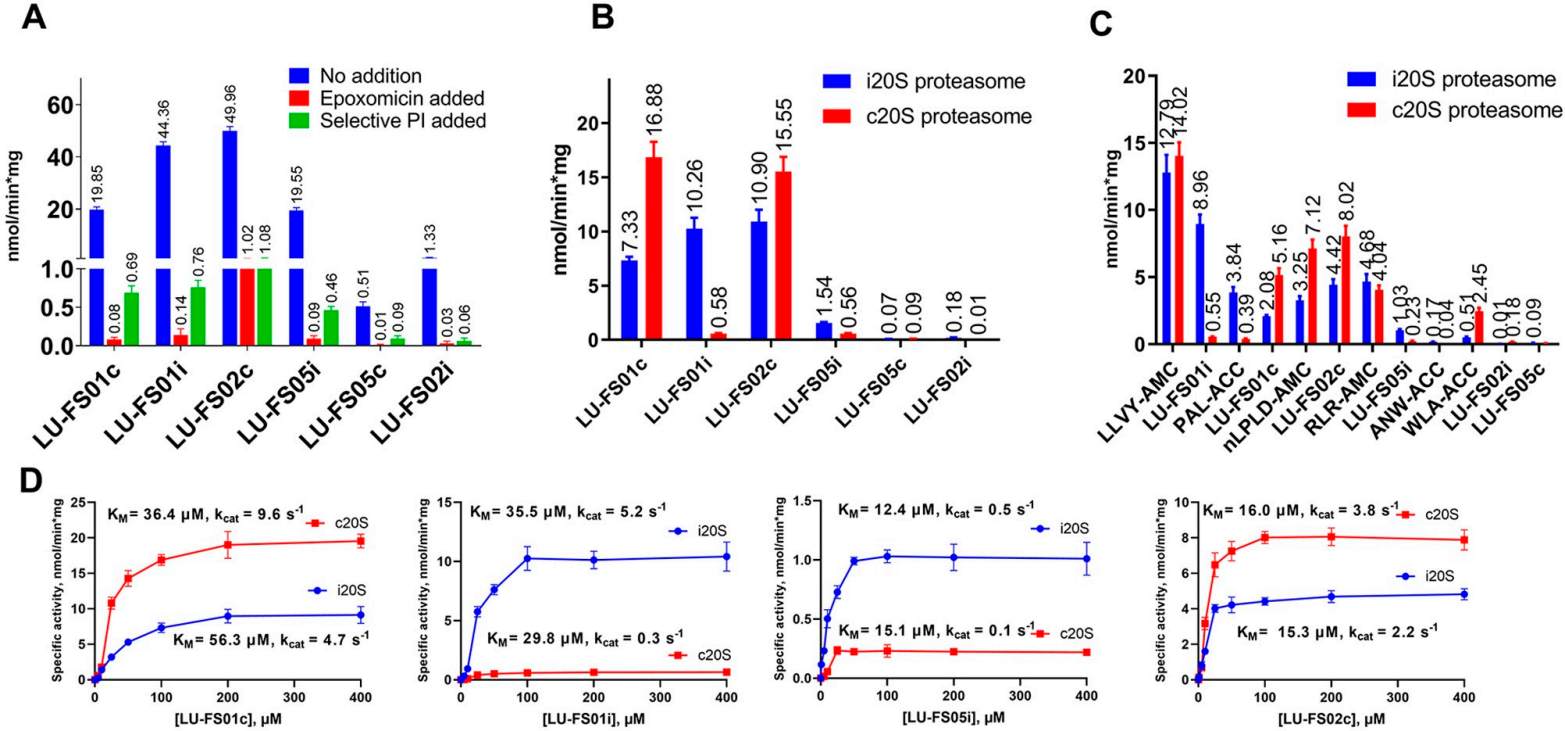
Validation and specific activities of synthesized and commercial compounds in lysates and purfified proteasome. A) Specifc activity in Raji lysate, with or without pre‐incubation of the lysate with a nonselective proteasome inhibitor (epoxomicin) or a subunit selective proteasome inhibitor. B) Hydrolysis of fluorogenic substrates in SDS activated purified c20S and i20S proteasome. Substrate hydrolysis conditions: Tris ⋅ HCl (pH 7.8) assay buffer, 2.33 nM 20S, 0.035 % SDS, 100 μM substrate concentration, 37 °C. C) Specific activity of synthesized and commercial fluorogenic substrates in PA28 activated purified 20S proteasome. Conditions: 23.3 nM PA28. D) Michaelis‐Menten characterization of (left to right) LU‐FS01c, LU‐FS01i, LU‐FS05i, and LU‐FS02c. Corresponding kinetic parameters are displayed in Table S1.

As a first evaluation of the efficacy of the synthesized peptides as fluorogenic proteasome substrates we treated Raji lysate (representing human B cell lymphoma) with these following the literature protocol (described in the Supporting Information).^[17]^ Raji lysate contain iCP and cCP, as well as other proteases.^[27]^ Measurement over time of the fluorescent signal that is the result of the released ACC/AMC group indicates proteasome activity (Figure 1A and Figure S1 in the Supporting Information). The resulting signals might, however, stem from proteasome‐mediated processing but also from other proteases able to process the fluorogenic substrates. To discriminate between proteasome‐generated fluorescence and turnover effected by other proteases the lysates were pre‐incubated with either the broad‐spectrum proteasome inhibitor, epoxomicin or a selective inhibitor complementary to the added fluorogenic substrate (e. g., LU‐FS01i and a β1i‐selective inhibitor).^[28]^ Proteasome selectivity of the applied inhibitors was established by activity‐based protein profiling using the set of activity‐based proteasome probes we reported previously, followed by SDS‐PAGE and fluorescent detection of the unmodified proteasome active sites (Figure S2).^[5]^

Figure 1A depicts selectivity and activity of the fluorogenic substrates from studies in which lysates were either pretreated with proteasome inhibitors or not. When lysate was treated with LU‐FS01c (**1**) fluorescence was observed, but not when inhibitor LU‐001c was included in the experiment. This strongly indicates that substrate LU‐FS01c indeed is processed by the intended proteasome subunit in a time‐dependent fashion and moreover that no other proteases significantly contribute to its turnover. The same holds true for LU‐FS01i, LU‐FS02c and LUFS05i, while for LU‐FS02c minor background activity was observed.

In contrast to the above substrates that were revealed to be highly effective and selective reporters on proteasome activities, LU‐FS05c and LU‐FS02i proved to be poor substrates (Figure 1A). However, their turnover can still be assigned to proteasome activity as pre‐incubation with either epoxomicin or subunit‐selective inhibitors abolished the emergence over time of fluorescence.

### Substrate hydrolysis by isolated 20s proteasomes

With the aim to obtain deeper insight in selectivity of the fluorogenic substrates towards proteasome subtype, substrate hydrolysis assays were next performed using purified iCP and cCP, termed i20S and c20S, respectively. To this end and following the literature precedents, 20S core particles were activated with 0.035 % SDS (Figures 1B and S4).^[29]^ The selectivity of the six substrates towards c20S and i20S active sites is depicted in Figure 1B and matches results obtained from measurements in Raji cell extracts. As before, LU‐FS05c and LU‐FS02i showed some selectivity towards the targeted proteasome active sites, but again proved to be poor substrates for these.

In the next experiment, assays were performed taking the newly synthesized fluorogenic substrates as well as commercial and PA28 activated purified proteasome (Figure 1C). All outcomes either correspond with literature or earlier measured results.^[30]^ Minor discrepancies with the results depicted in Figure 1B could be attributed to the different activation of the 20S particle (SDS vs PA28) and the possible subsequent different mode of action of the fluorogenic substrates. Commonly used fluorogenic substrates (for instance, LLVY‐AMC), are known to trigger gate opening and thus stimulate the activity of the 20S particles by themselves already.^[31]^ LU‐FS01c proved to outcompete its commercial counterpart (Ac‐nLPnLD‐AMC) with higher selectivity (c20S over i20S) and similar specific activities. LU‐FS01i, LU‐FS02c and LU‐FS05i all outcompete their commercial counterparts in both specific activity and selectivity.

Finally, Michaelis‐Menten kinetics were determined for the 4 most effective fluorogenic substrates from our new compounds: LU‐FS01c, LU‐FS01i, LU‐FS02c and LU‐FS05i (Figure 1D, Table S1).^[32]^ As can be seen *v*_max_ is generally reached at a substrate concentration of 100 μM, as is reported for most literature counterparts.^[17]^

## Conclusion

This work describes the translation from specific subunit‐selective proteasome inhibitors to fluorogenic substrates. The fluorogenic substrates were tested for activity and selectivity in biological assays on crude cell extracts and purified 20S proteasome. The fluorogenic substrates targeting the β2i and β5c subunits lack activity, possibly due to their low solubility in combination with high affinity and slow dissociation from the proteasome. In contrast, the other four compounds (LU‐FS01i, LU‐FS01c, LU‐FS02c, LU‐FS05i) showed high activity and selectivity in Raji (human B cell) lysates. Hydrolysis was completely suppressed by pre‐incubation with either a pan‐subunit selective proteasome inhibitor (epoxomicin) or their subunit selective inhibitor counterparts, thus indicating the selectivity of the synthesized substrates for the proteasome subunits they were designed to report on. In the past,^[33]^ we made the intriguing observation that selective and mechanism‐based inhibition of β5c in isolated 20S and 26S proteasomes led to an increase in β1c/β2c catalytic activity. Thus, crosstalk exists between the proteasome catalytic sites, and although this crosstalk might complicate interpretation of results obtained by fluorogenic substrate turnover measurements, the combination of selective inhibitors, activity‐based probes and fluorogenic substrates might also allow such effects to be probed in more detail.

Note: Our compounds are available upon request.

## Conflict of interest

The authors declare no conflict of interest.

## Supporting information

As a service to our authors and readers, this journal provides supporting information supplied by the authors. Such materials are peer reviewed and may be re‐organized for online delivery, but are not copy‐edited or typeset. Technical support issues arising from supporting information (other than missing files) should be addressed to the authors.

Supplementary
