# Supplementary material for: Structure‐Based Design of Fluorogenic Substrates Selective for Human Proteasome Subunits
Source: Chembiochem. 2020 Jul 29;21(22):3220–4. doi: 10.1002/cbic.202000375 (PMC7754458; doi:10.1002/cbic.202000375)

ChemBioChem

Supporting Information

Structure-Based Design of Fluorogenic Substrates Selective for Human Proteasome Subunits

Elmer Maurits, Christian G. Degeling, Alexei F. Kisselev, Bogdan I. Florea,* and Herman S. Overkleeft*

Supporting Information

Contents

Supplementary Figures 3
Proteasome activity assays 3
Parameters of Michaelis-Menten characterization 6
ClogP determination of fluorogenic substrates 7
Optimal ACC wavelength determination 7
Experimental Section 8
Biological procedures 8
Synthetic procedures 9
Synthesis of ACC fluorogenic substrates 10
Synthesis of AMC fluorogenic substrates 17
NMR Spectra 29
HRMS Data 53

Supplementary Figures

Proteasome activity assays

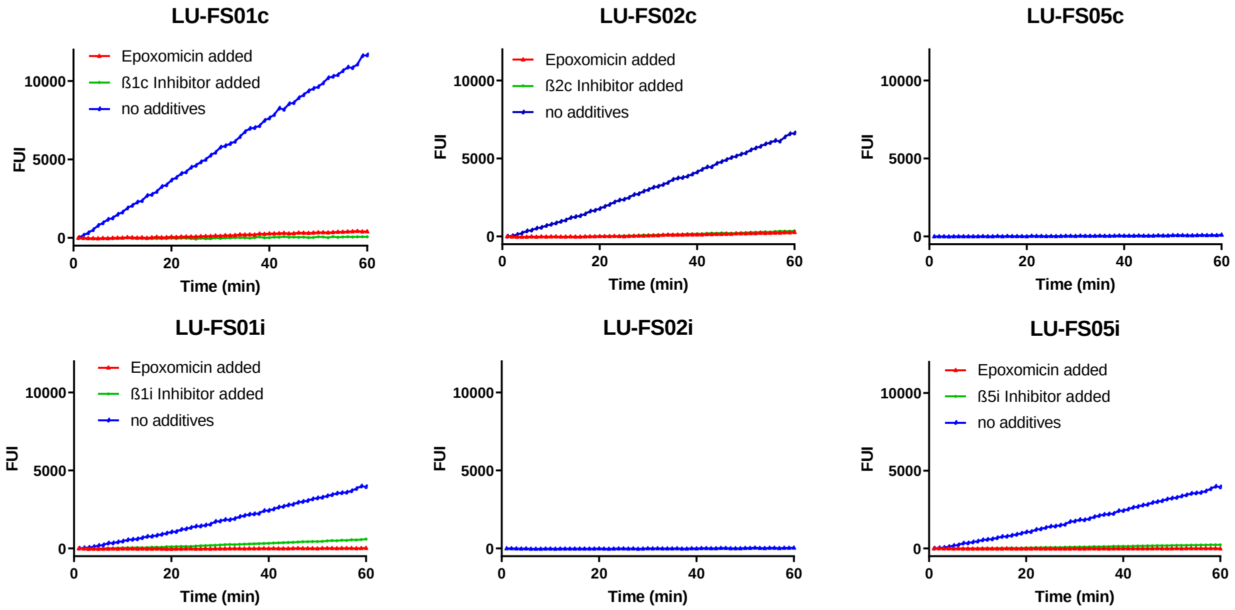

Figure S1, Fluorogenic substrate hydrolysis assay on Raji lysate in TRIS buffer. Substrate hydrolysis conditions: TRIS-HCl buffer (pH=7.5) assay-buffer, Raji lysate 10 µg/well, 10 µM Epoxomicin, 0.1-3 µM inhibitor, 100 µM fluorogenic substrate, 37°C.

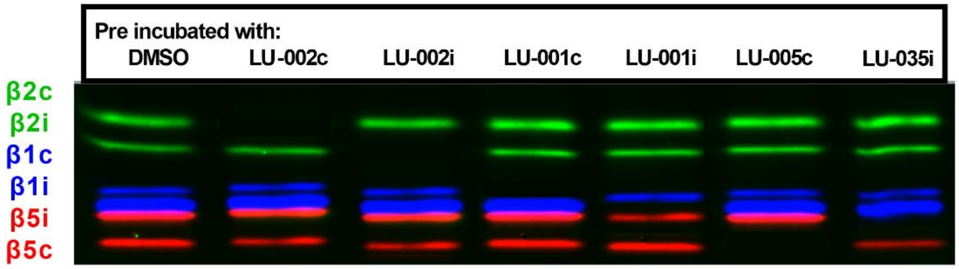

Figure S2, Competitive ABBP analysis to validate results measured in the fluorogenic substrate hydrolysis. LU-0XX are the selective proteasome inhibitors used in this experiment. SDS-PAGE cell lysate labeling conditions: TRIS-HCl buffer (pH=7.5), ABP-cocktail (100nm Cy5-NC-001, 30nM BODIPY(FL)-LU-112, 100nM BODIPY(TMR)-NC-005-VS), 37°C. 12.5% SDS-PAGE (TRIS-Glycerin), gels run (15 min at 80V, 120min at 130V).

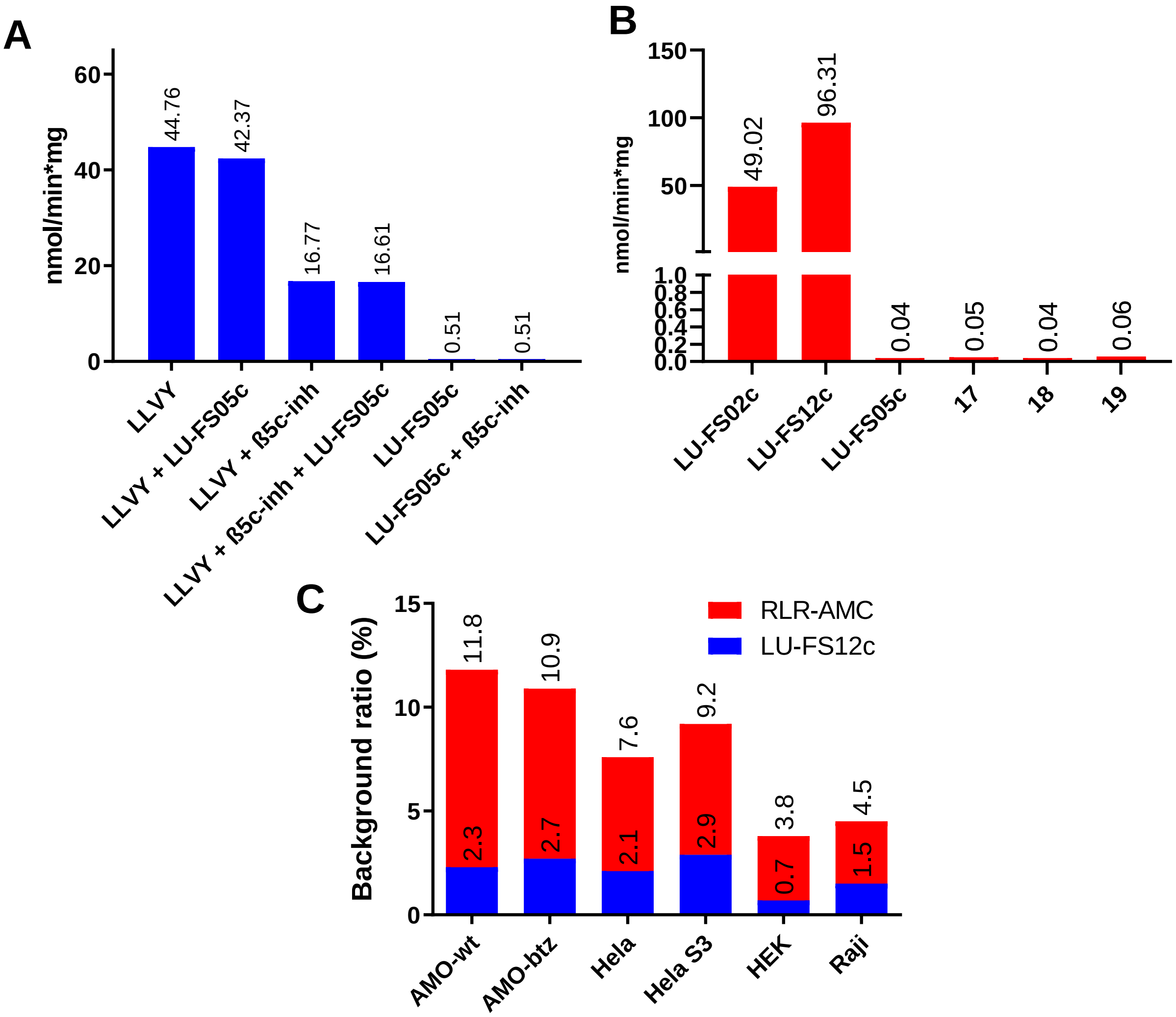

Figure S3, Additional control experiments. **A)** Activities of LU-FS05c versus LLVY-AMC to indicate a potential non-covalent inhibitory effect.* **B)** Activity of improved β2c and β5c fluorogenic substrates, measured in Raji cell lysate. **C)** Background ratio of fluorogenic activity in different lysates using the newly synthesized LU-FS12c versus commercially available RLR-AMC.

* LU-FS05c is not efficiently cleaved by the beta5c subunit. Because it is based on a powerful, selective inhibitor we hypothesised that the substrate has a high k_{on} and a very low k_{off} rate for the beta5c subunit, making it a competitive inhibitor. In order to assays this effect we have assayed the LLVY turnover in presence of the LU-FS05c substrate and found no reduction in LLVY turnover indicating that this inhibitory effect is not present.

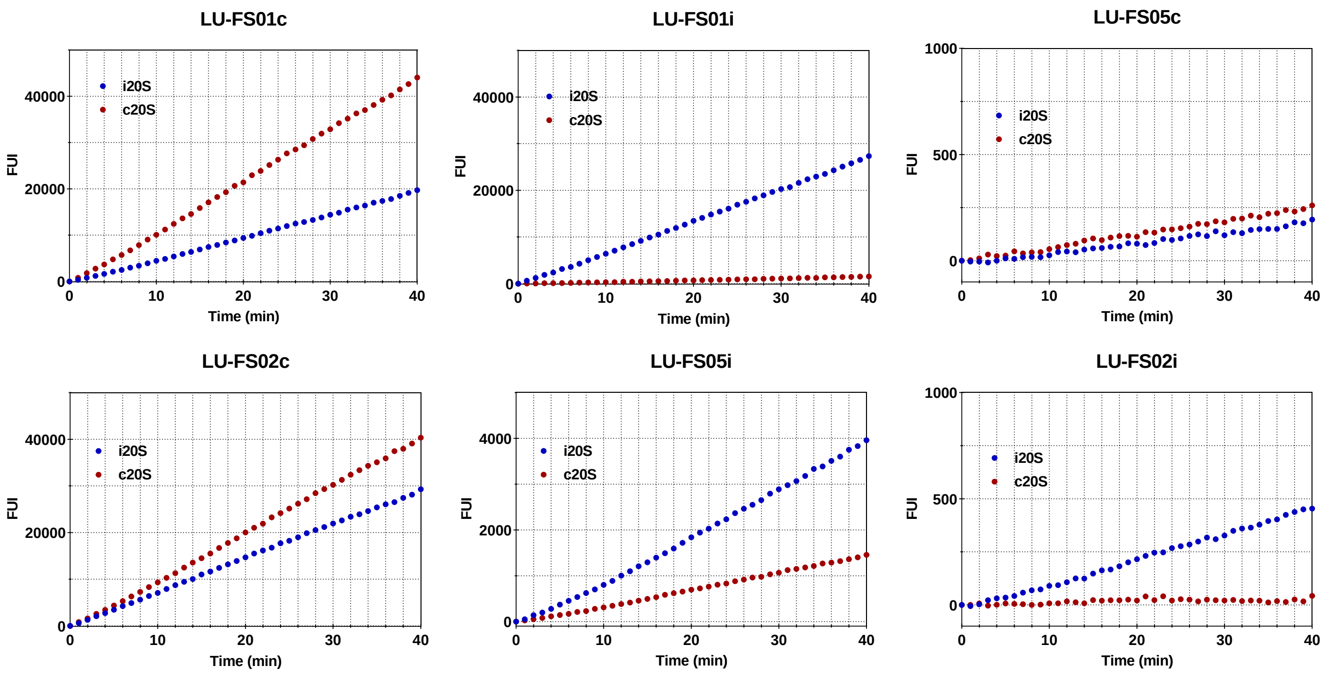

Figure S4, Purified i20S and c20S proteasome activity assay, Substrate hydrolysis conditions: HEPES-HCl (pH=7.8) assay buffer with 0.035% SDS, Proteasome 100 ng/well. 100µM final concentration of fluorogenic substrate, 37°C.

Parameters of Michaelis-Menten characterization

Table S1, Michaelis-Menten kinetics parameters determined in purified c20S and i20S. Calculated by Graphpad 8.1.1.

Substrate	K_m (μM)	k_{cat} (s^{-1})	V_{max} (nmol*min^{-1}*mg)	k_{cat}/K_m ($μM^{-1}s^{-1}$)	Ratio k_{cat}/K_m (i20S/c20S)	Ratio V_{max} (i20S/c20S)
**LU-FS01c**						
**i20S**	56.3	4.7	11.0	0.084	0.32	0.49
**c20S**	36.4	9.6	22.4	0.26		
**LU-FS01i**						
**i20S**	35.5	5.2	12.2	0.14	14	17
**c20S**	29.8	0.3	0.7	0.010		
**LU-FS05i**						
**i20S**	12.4	0.5	1.1	0.040	6.1	3.7
**c20S**	15.1	0.1	0.3	0.0066		
**LU-FS02c**						
**i20S**	15.3	2.2	5.2	0.14	0.58	0.58
**c20S**	16.0	3.8	8.9	0.24		

ClogP determination of fluorogenic substrates

Table S2, calculated logP values of fluorogenic substrates. In the first row CLogP was calculated with ALOGPS 2.1 and in the second row ClogP was calculated with Chemdraw 16.0

Compound	ClogP (ALOGPS)	ClogP (Chemdraw)
LU-FS01i	2.94 - 4.99	3.27
LU-FS01c	0.68 - 4.10	-0.25
LU-FS02i	3.63 - 5.43	4.28
LU-FS02c	2.36 - 5.61	1.33
LU-FS12c	3.18 - 4.87	4.72
LU-FS05i	4.98 - 6.06	5.3
LU-FS05c	4.42 - 5.23	7.63
**17**, LU-FS15c	5.47 - 5.76	8.86
**18**, LU-FS25c	4.09 - 4.91	9.99
**19**, LU-FS35c	2.22 - 5.77	- 2.48

Optimal ACC wavelength determination

Table S3, 3D-plot showing the optimal Ex/Em with the least amount of background signal from the ACC-peptide.

Background Signal (%)

Ex/Em(nm)	445	450	455
360	12,4	10,2	9,5
365	8,9	8,2	7,0
370	6,4	6,2	5,2
375	5,4	5,1	4,9

Relative Signal (kFU)

Ex/Em (nm)	445	450	455
360	9.9	10.1	9.4
365	10.3	10.7	10.2
370	8.8	9.3	8.6
375	7.3	7.6	7.2

Experimental Section

Biological procedures

Inhibitors and buffers used for the measurements of proteasome activity

Lysate assay buffer: 50 mM TRIS-HCl pH 7.5, 40 mM KCl, 5 mM $MgCl_2$, 0.5 mM Mg-ATP, 1 mM DTT, 0.05 mg/mL BSA, 50 µg/mL protein (as determined by Bradford assay)
20S purified proteasome assay buffer: HEPES-HCl pH 7.8*,* 10 mM NaCl, 1.5 mM $MgCl_2$, 1 mM EDTA, 250 mM sucrose, 5mM DTT, 0.05 mg/mL BSA, 100 ng/mL PA28 or 0.035% SDS, 50 ng/mL 20S proteasome (purchased from BostonBiochem, catalog number E-360 and E370)
Proteasome inhibitors: 10 µM epoxomicin (pan-subunit), 1 µM LU-001i (β1i), 1 µM LU-001c (β1c), 3 µM LU-002i (β2i), 0.1 µM LU-002c (β2c), 1 µM LU-005c (β5c) or 1 µM LU-035i (β5i)

Lysate preparation

Lysates of cells were prepared by treating cell pellets with 4 volumes of lysis buffer containing 50 mM TRIS-HCl pH 7.5, 2 mM DTT, 5 mM $MgCl_2$, 10% glycerol, 2 mM ATP, and 0.05% digitonin for 15-60 min, followed by centrifugation at 20000g to squeeze out the soluble proteome from the cells. Protein concentration was determined using Qubit® protein assay kit (Thermofisher).

Fluorogenic substrate activity assay

In a polypropylene 96-well plate, assay mixture is prepared by mixing 100 µL assay buffer (lacking purified proteasome or lysate) with 2 µL substrate (10 mM stock). Addition of 100 µL assay buffer (pre-mixed with lysate or purified 20S proteasome) yielded the desired final 100 µM substrate concentration. All activity measurements were directly recorded on a pre-heated TECAN plate reader at 37°C in quadruplo using λ_{ex} = 365 nm and λ_{em} = 450 nm for ACC substrates (as determined by Table S3) and λ_{ex} = 345 nm and λ_{em} = 445 nm for AMC substrates in 1 min intervals for a 2 h period. Michaelis-Menten calculations were performed using a non-linear fit in Prism v.6.0.
Pre-incubation of the lysate assay buffer with the desired proteasome inhibitor for 1 h at 37°C delivered the proteasome subunit inactivated lysate for substrate analysis.

Competitive ABPP by enzyme precipitation and SDS-PAGE

After the fluorogenic substrate activity assay, 10 µL 10x ABP-cocktail (Cy5-NC-001, BODIPY(FL)-LU-112, BODIPY(TMR)-NC-005-VS) was added to each well and incubated for 60 min at 37°C to label the residual active proteasome subunits. The wells were transferred to individual Eppendorf tubes, proteins were precipitated by $CHCl_3$/MeOH according to Wessel and Flüggel (doi: 10.1016/0003-2697(84)90782-6). The pellet was washed twice with PBS and dissolved in 20 µL 0.01% SDS in PBS followed by 5 min boiling with 10 µL gel-loading buffer. Electrophoresis was performed

on 12.5% SDS-PAGE gel for 15 min at 80 V and then 2 h at 130 V. On each gel, 2.5 µl of page ruler was used. Multiplex fluorescent detection of residual ABPs was performed on a ChemiDoc™ MP System with Cy5, Cy3 and Cy2 channels.

Synthetic procedures

All reagents were of commercial grade and used as received unless stated otherwise. Solvents used in synthesis were dried and stored over 4Å molecular sieves, except MeOH and ACN which were stored over 3Å molecular sieves. Triethylamine (Et_3N) and di-isopropylethylamine (DiPEA) were stored over KOH pellets.

Column chromatography was performed on silica gel 60 Å (40-63 µm, Macherey-Nagel). TLC analysis was performed on Macherey-Nagel aluminium sheets (silica gel 60 F_{254}).

TLC was used to monitor reactions with visualization by UV at wavelength 254 nm and by one of following treatments: spraying with either cerium molybdate spray (25 g/L $(NH4)_6Mo_7O_{24}$, 10 g/L $(NH4)_4Ce(SO_4)_4{\cdot}H_2O$ in 10% H_2SO_4 water solution) or $KMnO_4$ spray (20 g/L $KMnO_4$ and 10 g/L K_2CO_3 in water) followed by charring at c.a. 250°C. **TLC-MS** analysis was performed on an Advion Expression^L EMS in combination with a Plate Express interface (eluted with MeOH/H_2O 90%/10% v/v + 0.1% formic acid, flow rate 0.20 mL/min).

LC-MS analysis was performed on a Finnigan Surveyor HPLC system with a Nucleodur C18 Gravity 3um 50 x 4.60 mm column (detection at 200-600 nm) coupled to a Finnigan LCQ Advantage Max mass spectrometer with ESI or coupled to a Thermo LCQ Fleet Ion mass spectrometer with ESI. The Method used was 10→90% 13.5 min (0→0.5 min: 10% MeCN; 0.5→8.5 min: 10% to 90% MeCN; 8.5→ 11 min: 90% MeCN; 11→13.5 min: 10% MeCN).

High-resolution mass spectrometry (HRMS) was performed on a Thermo Scientific Q Exactive HF Orbitrap mass spectrometer equipped with an electrospray ion source in positive-ion mode (source voltage 3.5 kV, sheath gas flow 10, capillary temperature 275°C) with resolution R = 240.000 at m/z 400 (mass range of 150-6000) correlated to an external calibration, or on a Waters Synapt G2-Si (TOF) equipped with an electrospray ion source in positive mode (source voltage 3.5 kV) and LeuEnk (m/z = 556.2771) as internal lock mass.

^{1}H and ^{13}C NMR spectra were recorded on a Bruker AV-400 NMR, a Bruker DMX-400 NMR instrument (400 and 101 MHz respectively), a Bruker AV-500 NMR instrument (500 and 126 MHz respectively), and Bruker AV-600 NMR instrument (600 and 151 MHz respectively). Chemical shifts (δ) are given in ppm relative to tetramethylsilane as internal standard or the residual signal of the deuterated solvent. Coupling constants (*J*) are given in Hz. All given ^{13}C-APT spectra are proton decoupled. Assignment of NMR spectra was based on ^{1}H-COSY and ^{1}H-^{13}C-HSQC.

HPLC purification was performed on a Gilson HPLC system coupled to a Magerey-Nagel Nucleodur C18 Gravity 5 µm 250 ×10 mm column, or on an Agilent 1200

HPLC/6130 MS system coupled to a Magerey-Nagel Nucleodur C18 Gravity 5µm 250×10 mm column or on a Waters autopurifier HPCL/MS system coupled to a Phenomenex Gemini 5µm 150×21.2 mm column.

Synthesis of ACC fluorogenic substrates

Procedure for Fmoc protection during solid phase synthesis

The Fmoc-protected amino acids were prepared by treating the free amine with Fmoc-Cl in H_2O/Organic solvent system. The choice of organic solvent varied based upon which free amine was used (THF or 1,4-dioxane).
The free amine and $NaHCO_3$ (1.2 eq) were added to H_2O followed by the addition of the organic solvent. The H_2O/organic solvent ratio used was 1/1 (v/v). The solution was cooled in an ice bath and stirred rapidly before addition of Fmoc-Cl (1.2 eq). Fmoc-Cl was dissolved in either THF or 1,4-dioxane and slowly added to the reaction mixture. After addition of the Fmoc-Cl the reaction was stirred at 0^oC for at least 1h. Then, the reaction mixture was allowed to warm to RT and stirred overnight. TLC was used to monitor the reaction until its completion. The reaction mixture was evaporated *in vacuo* and the remaining solid was dissolved in H_2O. The solution was extracted with Et_2O (4x) and the aqueous phase was acidified to pH 2 with 2 M HCl and extracted with EtOAc (2x). The combined organic fractions were washed once with brine, dried over $MgSO_4$, filtered and evaporated *in vacuo*. The product was further purified using flash column chromatography.

Kaiser test procedure

A set of 3 reagents was made as a stock for the Kaiser test. Solution A; 500 mg ninhydrin in 10 mL EtOH. Solution B; 20 g phenol in 10 mL EtOH. Solution 3; 200 µL 1 mM solution of KCN diluted to a total volume of 10 mL with pyridine. Three drops of each solution together with a small amount of resin was added to a LC-MS vial, mixed and stored at 80^oC for 5 min. The solution should be either blue (indication of free amines) or yellow (no free amines).

ACC synthesis

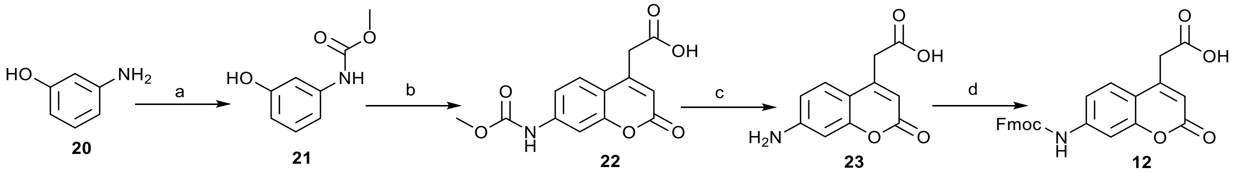

Scheme S2. Synthesis of Fmoc-ACC. Reagents and conditions: **a)** Methyl chloroformate (1.1 eq.), $NaHCO_3$, EtOAc/H_2O (15/1), rt, 1h, quant.; **b)** Citric acid (1.2 eq.), H_2SO_4,

70°C – 0°C, 18h, 63%; **c)** NaOH (10 eq.), H_2O, 100°C, 1h, 91%; **d)** Fmoc-Cl (1.1 eq), TMSCl (2.2 eq.), DiPEA (2.2 eq.), DCM, 45°C, 3h, 81%.

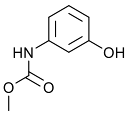

Methyl (3-hydroxyphenyl)carbamate (21). 3-aminophenol (**20**) (30.5 g, 183 mmol), m-aminophenol (20.0 g, 183 mmol) and sodium bicarbonate (18.1 g, 219 mmol) were suspended in 15:1 EtOAc/H_2O mixture with a total volume of 280 mL at 0°C. Next, Methyl chloroformate (20.1 g, 212 mmol) was added over the course of 0.5 h with rapid stirring. After addition of methyl chloroformate the mixture was stirred for an additional 1 h after which H_2O (5 mL) was added followed by stirring for an additional 3 h. The organic layer was separated and washed with H_2O, followed by washing with 1 M H_2SO_4, H_2O and brine. The organic extract was dried over $MgSO_4$, filtered and evaporated *in vacuo* yielding the title compound as a pale solid (27.6 g, 165 mmol, 90%). Rf 0.5 1/20 MeOH/DCM. ^{1}H NMR (400 MHz, $CDCl_3$) δ 7.38 (s, 1H), 7.14 (t, *J* = 8.1 Hz, 1H), 6.74 (s, 1H), 6.61 (dddd, *J* = 20.3, 8.2, 2.3, 0.9 Hz, 3H), 3.79 (s, 3H). ^{13}C NMR (101 MHz, $CDCl_3$) δ 156.7, 138.5, 129.8, 110.6, 52.5.

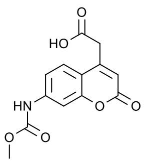

3-methoxycarbonylamino-4-aceticacidcoumerine (22). Citric acid (28.8 g, 150 mmol) was added to concentrated H_2SO_4 (33 mL). The mixture was heated to 70°C over the course of 0.5 h. The formation of 1,3-acetonedicarboxylic acid was indicated by the release of gas. After the evolution of gas had slackened, the solution was cooled to 0°C using an ice bath. The reaction mixture turned bright yellow. Compound **21** (20.0 g, 112 mmol) was added slowly over the course of 0.5 h. Then, concentrated H_2SO_4 (13.5 mL) was added. The reaction mixture was stirred overnight at 0°C using a cryostat. The reaction was poured onto ice and the mixture was stirred until crystallization ceased. The crystals were filtered and washed with H_2O, cold MeOH, Et_2O and dried in a vacuum oven. This yielded the title compound as a white solid (18.5 g, 82 mmol, 56%). ^{1}H NMR (400 MHz, DMSO-d_6) δ 12.80 (s, 1H), 10.19 (s, 1H), 7.63 (d, *J* = 8.8 Hz, 1H), 7.57 (d, *J* = 2.1 Hz, 1H), 7.38 (dd, *J* = 8.8, 2.1 Hz, 1H), 6.33 (s, 1H), 3.86 (s, 2H), 3.71 (s, 4H). ^{13}C NMR (101 MHz, DMSO-d_6) δ 170.63, 160.02, 153.83, 149.91, 142.83, 126.20, 113.70, 104.53, 37.04.

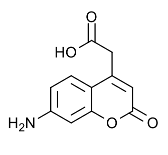

4-aminocoumerin-4-acetic acid (23). Compound **22** (18.5 g, 82 mmol) was added to a round-bottom flask equipped with a reflux condenser, it was dissolved in H_2O (170 mL) containing NaOH (40 g, 650 mmol). The reaction mixture was stirred overnight under reflux conditions. The reaction was cooled to 0°C and adjusted to pH 2 by dropwise addition of conc. HCl. The mixture slowly turned cloudy. The next morning the mixture contained crystals which were collected by filtration. The resulting mixture turned cloudy during the day and contained crystals the next morning. Again, the crystals were collected by filtration and this step was repeated once more the crystals were washed with cold H_2O and Et_2O and were

dried in a vacuum oven to yield **3** (22.3 g, 80 mmol, 97%) as a fluffy bright yellow powder. ^{1}H NMR (400 MHz, DMSO-*d*$_6$) δ 12.73 (s, 1H), 7.33 (d, *J* = 8.7 Hz, 1H), 6.55 (dd, *J* = 8.6, 2.2 Hz, 1H), 6.42 (d, *J* = 2.1 Hz, 1H), 6.16 (s, 2H), 5.98 (s, 1H), 3.73 (s, 2H). ^{13}C NMR (101 MHz, DMSO-*d*$_6$) δ 170.9, 160.8, 155.7, 153.2, 150.4, 126.4, 111.3, 109.0, 108.2, 98.6, 37.3.

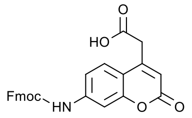

N-(Fluorenylmethoxycarbonyl)aminocoumarin-4-acetic acid (12). Compound **23** (1.50 g, 6.87 mmol) and DCM (40 mL) were added to a 250 mL round-bottom flask equipped with a reflux condenser. Next, TMSCl (1.63 g, 15.1 mmol) and DIPEA (1.94 g, 15.1 mmol) were added while stirring and the reaction was heated to reflux for 3 h. After reflux the reaction mixture was cooled to 0ºC using an ice bath after which Fmoc-Cl (1.94 g, 7.53 mmol) was added in small portions. The reaction was left to stir at 0ºC for 1 h before it was allowed to warm to RT and stirred overnight. Addition of MeOH caused the formation of a white precipitate. The product was collected by filtration and washed with cold MeOH, Et_2O and dried in a vacuum oven to yield compound **5** (2.71 g, 6.16 mmol, 90%). ^{1}H NMR (400 MHz, DMSO-*d*$_6$) δ 12.80 (s, 1H), 10.20 (s, 1H), 7.91 (d, *J* = 7.5 Hz, 2H), 7.76 (d, *J* = 7.4 Hz, 2H), 7.62 (d, *J* = 8.7 Hz, 1H), 7.55 (s, 1H), 7.39 (dt, *J* = 29.3, 7.4 Hz, 5H), 6.33 (s, 1H), 4.56 (d, *J* = 6.4 Hz, 2H), 4.34 (t, 1H), 3.86 (s, 2H). HRMS calculated for $C_{28}H_{33}NO_4$ 442.12856 $[M+H]^+$; found 442.12854.

Solid phase peptide synthesis compound library

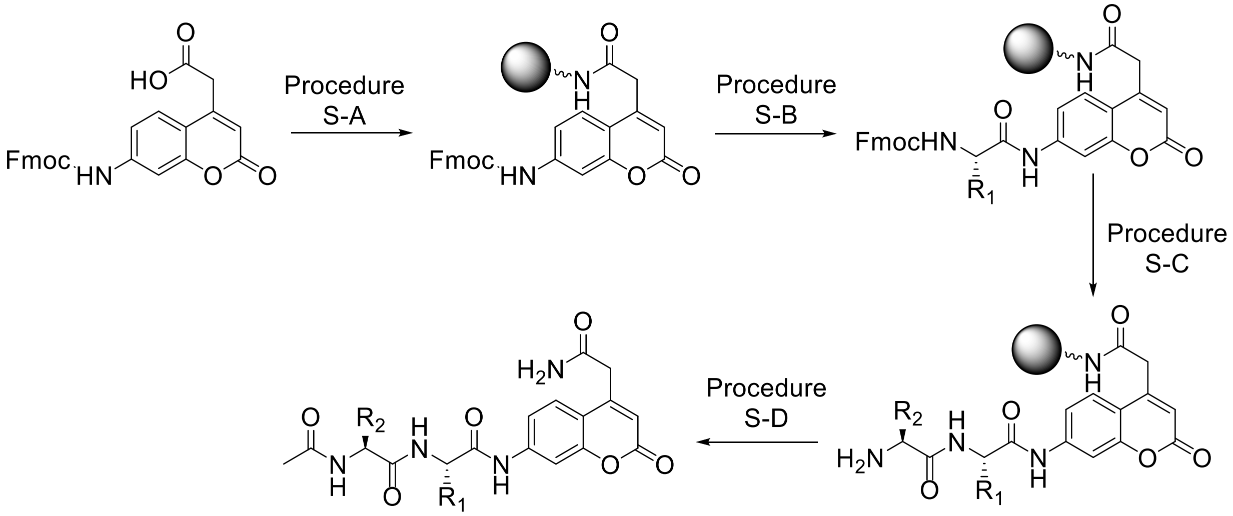

Scheme S3, General solid-phase peptide synthesis route towards peptide-ACC fluorogenic substrate. Reagents and conditions of procedures: **S-A)** i) Piperidine, DMF; ii) Fmoc-ACC, HOBt, DICI, DMF; **S-B)** i) Piperidine, DMF; ii) Fmoc-AA-OH, HATU, Collidine, DMF, **S-C)** i) Piperidine, DMF; ii) Fmoc-AA-OH, HOBt, DICI, DMF iii) Piperidine, DMF; **S-D)** i) Ac_2O, Pyr, DMF; ii) TFA, DCM, prep-HPLC purification

Procedure S-A: ACC-Rink Amide resin

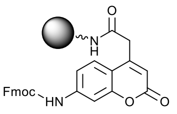

Rink Amide resin (5.03 g, 1.21 mmol) was placed in two 20 mL syringes equipped with a filter and the resin was gently shaken in DMF for 0.5 h using a mechanical shaker. After filtration and washing with DMF (3x), a solution of 20% piperidine in DMF was added and the resin was shaken for 5 min. The resin was filtered, washed with DMF (3x) and new 20% piperidine in DMF was added. After 5 min the resin was filtered, washed with DMF (3x) and fresh 20% Piperidine in DMF was added and the resin was again shaken for 25 min followed by filtration and washing with DMF (6x). Compound **5** (1.32 g, 3.01 mmol), DICI (378 mg, 3.01 mmol), HOBt (465 mg, 3.01 mmol) were pre-activated in THF and added to the resin. The reaction mixture was stirred overnight. The resin was filtered and washed with DMF (6x) and then the reaction was repeated with compound **5** (1.32 g, 3.01 mmol), DICI (378 mg, 3.01 mmol), HOBt (465 mg, 3.01 mmol) in THF respectively to improve the yield of ACC coupling to the resin. After the second coupling the resin was washed with DMF (6x), MeOH (3x), DCM (3x) and Et_2O (2x). Kaiser test was used to indicate completion of the coupling to the resin. For N-terminus acetylation, a capping solution of 5% (v/v %) Ac_2O, 0.1 M DIPEA in DMF was used and the reaction was gently shaken for 0.5 h. A small amount of resin was subjected to cleavage using a mixture of TFA:TIPS:H_2O (v/v/v % 95:2.5:2.5). The resin was cleaved in a LC-MS vial using 1 drop of cleaving solution and after the resin turned red, a mixture of H_2O:ACN:tBuOH (v/v/v % 1/1/1) was added and mixed as a sample for LC-MS analysis.

Procedure S-B: AA_1-ACC-Rink Amide resin

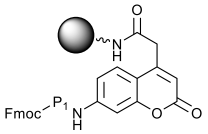

To a syringe equipped with a filter containing ACC-Rink Amide resin (0.2 mmol), 20% piperidine/DMF was added (3x after 5, 15 and 25 min) with washing with DMF (3x) between each deprotection. After removal of the Fmoc-group, amino acid (0.8 mmol), HATU (304 mg, 0.8 mmol) and 2,4,6-trimethylpyridine (97 mg, 0.8 mmol) in DMF were activated for 2 min and added to the ACC-Rink amide resin. The reaction was gently shaken overnight, and the resin was washed with DMF (6x). At this point the resin was subjected to a Kaiser test using the described procedure. After the Kaiser test a capping solution (5% Ac_2O, 0.1 M DIPEA in DMF) was added to the resin and the reaction was gently shaken for 30 min. After the reaction the resin was washed with DMF (3x), MeOH (3x), DCM (3x) and Et_2O (2x). A small amount of resin was subjected to cleavage using a mixture of TFA:TIPS:H-$_2$O (v/v/v % 95:2.5:2.5). The resin was cleaved in a LC-MS vial using 1 drop of cleaving solution and after the resin turned red, a mixture of H_2O:ACN:tBuOH (v/v/v % 1/1/1) was added and mixed as a sample for LC-MS analysis to confirm the AA coupling.

Procedure S-C: P_2, P_3, P_4 – ACC-Rink Amide resin

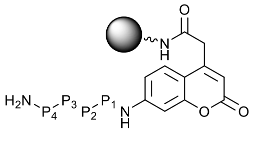

To a syringe equipped with a filter containing AA_1-ACC-Rink Amide resin (0.2 mmol), 20% piperidine/DMF was added (3x after 5, 15 and

25 min) with washing with DMF (3x) between each deprotection. After removal of the Fmoc-group, amino acid (0.8 mmol), HOBt (108 mg, 0.8 mmol) and DICI (101 mg, 0.8 mmol) in DMF were activated for 2 min and added to the ACC-Rink amide resin. The reaction was gently shaken overnight and the resin was washed with DMF (6x). At this point the resin was subjected to a Kaiser test using the described procedure. After the Kaiser test 20% piperidine in DMF was added (3x after 5, 15 and 25 min) to remove the Fmoc-group followed by addition of a capping solution (5% AC_2O, 0.1 M DIPEA in DMF) and the reaction was gently shaken for 30 min. After the reaction the resin was washed with DMF (3x), MeOH (3x), DCM (3x) and Et_2O (2x). A small amount of resin was subjected to cleavage using a mixture of TFA:TIPS:H_2O (v/v/v % 95:2.5:2.5). The resin was cleaved in a LC-MS vial using 1 drop of cleaving solution and after the resin turned red, a mixture of H_2O:ACN:tBuOH (v/v/v % 1/1/1) was added and mixed as a sample for LC-MS analysis to confirm the AA coupling. This procedure was repeated until all desired AAs were coupled.

Procedure S-D: Fluorogenic substrate cleavage from Rink amide resin

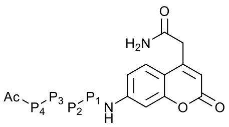

After complete synthesis of the fluorogenic substrate-rink amide resin, the peptide was cleaved from the resin using a mixture of TFA:TIPS:H_2O (v/v/v % 95:2.5:2.5). The resin was shaken for 2 h after which it was added to Et_2O (20 mL, in corning tube). A white precipitant would become visible and the resin was washed once more with a small amount of cleaving mixture. The resultant precipitant was centrifuged using an ultra-centrifuge at 5000 RPM for 15 min. The excess Et_2O was removed and the precipitant was washed once more with Et_2O. After centrifugation (5000 RPM for 15 min), the Et_2O was discarded and the resulting with solid was dried before purification by prep-HPLC. The purified peptides were evaporated under *vacuo* and co-evaporated 5 times with milli-Q water. The purified product was dissolved in ACN/H_2O (1:4 v/v) and lyophilized.

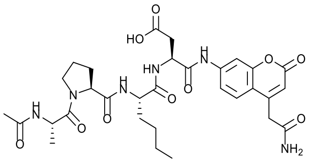

LU-FS01c, Ac-Ala-Pro-Nle-Asp-ACC (1). ^{1}H NMR (500 MHz, DMSO-d_6) δ 12.49 (s, 1H), 10.03 (s, 1H), 8.24 (d, *J* = 7.5 Hz, 1H), 8.13 (d, *J* = 7.4 Hz, 1H), 8.09 (d, *J* = 6.6 Hz, 1H), 7.82 (d, *J* = 2.0 Hz, 1H), 7.70 (d, *J* = 8.8 Hz, 1H), 7.66 (d, *J* = 2.4 Hz, 1H), 7.58 (dd, *J* = 8.8, 2.0 Hz, 1H), 7.20 (d, *J* = 2.4 Hz, 1H), 6.31 (s, 1H), 4.67 – 4.60 (m, 1H), 4.53 (t, *J* = 7.1 Hz, 1H), 4.34 (dd, *J* = 8.2, 3.8 Hz, 1H), 4.08 (dd, *J* = 6.3, 2.2 Hz, 1H), 3.66 (s, 2H), 3.63 – 3.58 (m, 1H), 3.57 – 3.50 (m, 1H), 2.84 (dd, *J* = 16.7, 5.5 Hz, 1H), 2.68 (dd, *J* = 16.7, 8.1 Hz, 1H), 2.54 (s, 3H), 2.03 (dd, *J* = 8.0, 3.4 Hz, 1H), 1.90 – 1.76 (m, 6H), 1.64 (d, *J* = 5.9 Hz, 1H), 1.54 (ddd, *J* = 13.3, 10.1, 4.7 Hz, 1H), 1.32 – 1.21 (m, 4H), 1.19 (d, *J* = 6.9 Hz, 3H), 0.90 – 0.76 (m, 3H). ^{13}C NMR (126 MHz, DMSO-d_6) δ 172.9, 172.3, 172.3, 171.8, 170.6, 170.2, 169.4, 160.5, 154.3, 151.3, 142.5, 115.3, 47.3, 39.1, 36.1, 31.5, 29.4, 27.8, 25.1, 22.5. HRMS calculated for $C_{36}H_{48}N_6O_8$ 679.27043 $[M+H]^+$; found 679.27047.

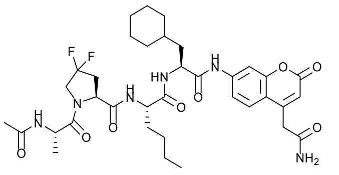

LU-FS01i, Ac-A-4,4-Difluoroproline-Nle-Cha-ACC (3). ^{1}H NMR (500 MHz, DMSO-d_6) δ 10.28 (s, 1H), 8.24 (d, *J* = 7.0 Hz, 1H), 8.11 (d, *J* = 7.3 Hz, 1H), 8.05 (d, *J* = 7.8 Hz, 1H), 7.81 (d, *J* = 2.1 Hz, 1H), 7.70 (d, *J* = 8.7 Hz, 1H), 7.66 (d, *J* = 2.2 Hz, 1H), 7.49 (dd, *J* = 8.8, 2.1 Hz, 1H), 7.19 (d, *J* = 2.2 Hz, 1H), 6.31 (s, 1H), 4.58 (dd, *J* = 9.2, 6.1 Hz, 1H), 4.48 – 4.41 (m, 2H), 4.30 (td, *J* = 12.8, 7.7 Hz, 1H), 4.19 (td, *J* = 7.9, 5.7 Hz, 1H), 4.00 (dt, *J* = 17.9, 11.1 Hz, 1H), 3.50 (d, *J* = 3.2 Hz, 1H), 2.83 – 2.68 (m, 1H), 2.44 – 2.26 (m, 1H), 1.81 (s, 3H), 1.65 (d, *J* = 8.8 Hz, 5H), 1.61 – 1.52 (m, 4H), 1.34 (d, *J* = 9.2 Hz, 1H), 1.29 – 1.22 (m, 5H), 1.17 (d, *J* = 6.9 Hz, 3H), 1.12 (d, *J* = 8.7 Hz, 2H), 0.99 – 0.86 (m, 2H), 0.84 (q, *J* = 4.0 Hz, 3H). ^{13}C NMR (126 MHz, DMSO-d_6) δ 172.4, 172.2, 172.1, 170.3, 170.2, 169.7, 160.5, 158.8, 158.5, 154.3, 151.3, 142.6, 127.8, 115.2, 70.3, 54.0, 53.8, 39.4, 39.1, 37.5, 37.3, 37.1, 33.7, 32.1, 32.0, 31.6, 28.0, 27.8, 26.6, 26.4, 26.1, 22.5, 22.6. HRMS calculated for $C_{36}H_{48}F_2N_6O_8$ 731.35968 $[M+H]^+$; found 731.35748.

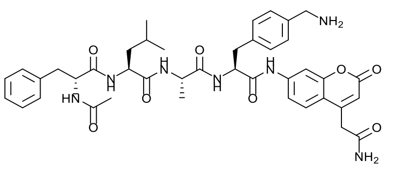

LU-FS02c, Ac-Phe-Leu-Ala-Phe(Boc-4-aminomethyl)-ACC (5). ^{1}H NMR (500 MHz, DMSO-d6) δ 10.55 (s, 1H), 8.30 – 8.16 (m, 4H), 8.11 (d, J = 8.1 Hz, 2H), 7.97 (d, J = 7.1 Hz, 1H), 7.79 (d, J = 2.0 Hz, 1H), 7.74 – 7.65 (m, 2H), 7.45 (dd, J = 8.7, 2.0 Hz, 1H), 7.39 – 7.31 (m, 4H), 7.24 (d, J = 4.4 Hz, 4H), 7.21 – 7.14 (m, 2H), 6.32 (s, 1H), 4.62 (td, J = 8.0, 5.5 Hz, 1H), 4.49 (ddd, J = 10.1, 8.2, 4.0 Hz, 1H), 4.31 – 4.17 (m, 2H), 3.98 (q, J = 5.8 Hz, 2H), 3.11 (dd, J = 13.8, 5.3 Hz, 1H), 3.01 – 2.90 (m, 2H), 2.70 (dd, J = 14.2, 10.4 Hz, 1H), 1.74 (s, 3H), 1.57 (dp, J = 12.7, 6.1 Hz, 1H), 1.51 – 1.40 (m, 2H), 1.21 (d, J = 7.0 Hz, 3H), 0.84 (dd, J = 25.0, 6.5 Hz, 6H). ^{13}C NMR (126 MHz, DMSO-d6) δ 172.44, 171.76, 171.53, 170.60, 169.70, 169.39, 160.03, 153.81, 150.85, 141.98, 138.04, 137.77, 132.19, 114.83, 42.05, 37.34. HRMS calculated for $C_{41}H_{49}N_7O_8$ 768.37390 $[M+H]^+$; found 768.37156.

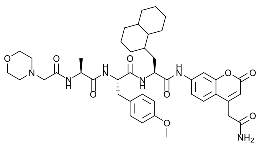

LU-FS02i, Morph-Ala-Tyr(Me)-1-DecAla-ACC (7). ^{1}H NMR (500 MHz, DMSO-d6) δ 10.50 (s, 1H), 8.76 (d, J = 7.4 Hz, 1H), 8.22 (dd, J = 20.7, 8.0 Hz, 2H), 7.80 (dd, J = 4.6, 2.0 Hz, 1H), 7.72 (d, J = 8.8 Hz, 1H), 7.70 – 7.64 (m, 1H), 7.46 (dt, J = 8.8, 2.5 Hz, 1H), 7.19 (s, 1H), 7.14 (dd, J = 8.6, 3.4 Hz, 2H), 6.77 – 6.66 (m, 2H), 6.32 (s, 1H), 4.50 (d, J = 7.5 Hz, 2H), 4.47 – 4.41 (m, 1H), 4.41 – 4.33 (m, 2H), 3.66 (s, 4H), 3.62 (d, J = 5.6 Hz, 5H), 2.95 (dd, J = 13.7, 5.3 Hz, 1H), 2.72 (dd, J = 13.8, 9.2 Hz, 1H), 1.69 (d, J = 12.6 Hz, 3H), 1.65 – 1.51 (m, 5H), 1.46 (s, 4H), 1.35 (s, 3H), 1.28 (d, J = 7.8 Hz, 2H), 1.21 (d, J = 7.0 Hz, 5H), 1.18 – 1.10 (m, 3H), 1.06 (t, J = 13.6 Hz, 1H). ^{13}C NMR (126 MHz, DMSO-d6) δ 171.79, 171.45, 171.06, 169.67, 163.41, 160.06, 158.57, 158.30, 158.03, 157.77, 153.83, 150.88, 142.16, 129.39, 69.81, 63.06, 56.54, 51.82, 38.58, 36.68, 26.38, 26.14, 25.00, 20.88, 19.66. HRMS calculated for $C_{43}H_{55}N_6O_9$ 801.42109 $[M+H]^+$; found 801.41820.

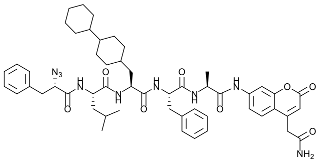

LU-FS05c, N_3Phe-Leu-BiCha-Phe-Ala-ACC (9). ^{1}H NMR (500 MHz, DMSO-d_6) δ 10.79 (s, 1H), 10.38 (s, 1H), 8.16 (d, *J* = 6.8 Hz, 1H), 8.05 (d, *J* = 8.0 Hz, 2H), 7.79 (d, *J* = 2.0 Hz, 1H), 7.70 (d, *J* = 8.7 Hz, 1H), 7.65 (s, 1H), 7.59 (d, *J* = 7.9 Hz, 1H), 7.47 (dd, *J* = 8.8, 2.0 Hz, 1H), 7.38 – 7.26 (m, 2H), 7.20 (d, *J* = 17.7 Hz, 2H), 7.13 (t, *J* = 5.0 Hz, 2H), 7.08 – 6.93 (m, 3H), 6.56 (s, 2H), 6.31 (s, 1H), 4.56 – 4.50 (m, 1H), 4.41 – 4.32 (m, 2H), 3.65 (s, 2H), 1.76 (d, *J* = 9.2 Hz, 3H), 1.63 – 1.57 (m, 1H), 1.49 (t, *J* = 7.3 Hz, 2H), 1.33 (d, *J* = 7.1 Hz, 3H), 0.87 (dd, *J* = 20.5, 6.5 Hz, 6H). HRMS calculated for $C_{53}H_{67}N_9O_8$ 958.52093 [M+H]$^+$; found 958.51858.

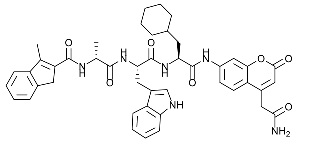

LU-FS05i, 3-methylidene-2-carboxamide-AW-Cha-ACC (10). ^{1}H NMR (500 MHz, DMSO-d6) δ 10.84 (d, J = 2.4 Hz, 1H), 10.33 (s, 1H), 8.17 (d, J = 7.8 Hz, 1H), 7.99 (d, J = 7.6 Hz, 1H), 7.85 (d, J = 6.9 Hz, 1H), 7.82 (d, J = 2.0 Hz, 1H), 7.73 – 7.63 (m, 2H), 7.55 (d, J = 7.9 Hz, 1H), 7.50 – 7.46 (m, 3H), 7.35 (td, J = 7.4, 1.3 Hz, 1H), 7.32 (dd, J = 7.3, 1.3 Hz, 1H), 7.29 (d, J = 2.6 Hz, 2H), 7.27 – 7.25 (m, 1H), 7.19 (d, J = 6.3 Hz, 4H), 7.15 (d, J = 2.3 Hz, 1H), 7.08 (s, 2H), 6.99 (ddd, J = 8.2, 7.0, 1.2 Hz, 1H), 6.90 (ddd, J = 8.0, 6.9, 1.0 Hz, 1H), 6.31 (s, 1H), 4.56 (td, J = 7.8, 5.1 Hz, 1H), 4.49 (q, J = 7.6 Hz, 1H), 4.39 (p, J = 7.1 Hz, 1H), 3.66 (s, 2H), 3.62 (dd, J = 11.1, 2.5 Hz, 2H), 3.19 (dd, J = 14.8, 5.0 Hz, 1H), 3.05 (dd, J = 14.8, 8.2 Hz, 1H), 2.37 (t, J = 2.3 Hz, 3H), 1.75 – 1.62 (m, 2H), 1.59 – 1.52 (m, 4H), 1.29 (d, J = 7.1 Hz, 5H), 1.09 (p, J = 12.4, 11.7 Hz, 3H), 0.85 (dq, J = 22.7, 11.1, 10.1 Hz, 2H). ^{13}C NMR (126 MHz, DMSO-d6) δ 173.19, 172.40, 171.88, 170.20, 165.99, 160.57, 158.63, 158.38, 154.33, 151.35, 145.75, 145.66, 142.75, 142.65, 136.55, 133.69, 127.92, 115.24, 110.26, 39.09, 38.71, 33.64, 32.32, 27.67, 26.54, 26.26, 26.04. HRMS calculated for $C_{45}H_{48}N_6O_7$ 785.36627 [M+H]$^+$; found 785.36681.

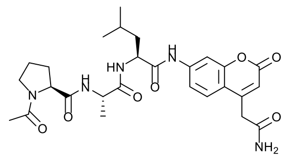

Ac-PAL-ACC (23). ^{1}H NMR (500 MHz, DMSO-d_6) δ 8.15 (d, *J* = 6.9 Hz, 1H), 7.88 (d, *J* = 7.8 Hz, 1H), 7.81 (d, *J* = 2.0 Hz, 1H), 7.71 (dd, *J* = 8.8, 3.4 Hz, 1H), 7.66 (s, 1H), 7.54 (dd, *J* = 8.8, 2.0 Hz, 1H), 7.19 (s, 1H), 6.31 (s, 1H), 4.37 (ddd, *J* = 10.7, 7.1, 3.6 Hz, 2H), 4.25 (dd, *J* = 8.7, 3.7 Hz, 1H), 4.20 (q, *J* = 7.1 Hz, 1H), 3.65 (s, 4H), 3.62 – 3.56 (m, 2H), 3.48 (dt, *J* = 9.6, 6.9 Hz, 1H), 2.21 – 2.05 (m, 1H), 2.00 (s, 3H), 1.89 – 1.84 (m, 2H), 1.70 – 1.60 (m, 2H), 1.61 – 1.48 (m, 2H), 1.25 (q, *J* = 7.3, 6.6 Hz, 4H), 0.98 – 0.84 (m, 8H). HRMS calculated for $C_{27}H_{35}N_5O_7$ 542.26068 [M+H]$^+$; found 542.26094.

Synthesis of AMC fluorogenic substrates

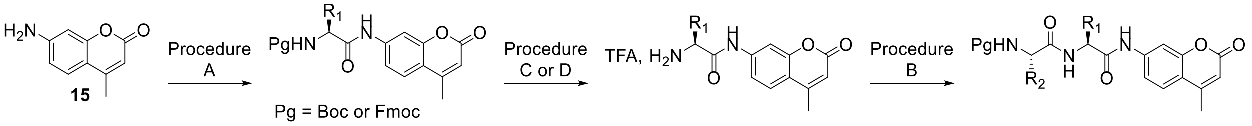

Scheme S1. General synthetic route towards peptide-AMC fluorogenic substrate. Reagents and conditions: **A)** $POCl_3$, Pyr, Pg-AA-OH, THF; **B)** HCTU, DiPEA, Boc-AA-OH; **C)** TFA/DCM; **D)** NaN_3, DMF, 50°C

Procedure A: AA-coupling to AMC

AMC **15** (1.2 eq.) and free acid (1.0 eq.) were dissolved in dry THF and cooled to 0ºC. Pyridine (10 eq.) was added, followed by slow addition of $POCl_3$ (3.7 eq.) over the course of 1 h. After stirring for an additional 1.5 h, the reaction was quenched by the addition of sat. aq. $NaHCO_3$. Subsequently, THF was evaporated under *vacuo* and the mixture was redissolved in EtOAc, washed with 1 M HCl (2x), sat. aq. $NaHCO_3$ (2x) and brine. The organic layer was dried over $MgSO_4$, filtered and concentrated in *vacuo* and used without further purification.

Procedure B: AA-coupling

Free amine (1.0 eq.) and free acid (1.2 eq.) were dissolved in DCM, followed by addition of HCTU (1.2 eq.) and DiPEA (3.5 eq.). After stirring overnight, the reaction mixture was concentrated in *vacuo* and re-dissolved in EtOAc, washed with 1 M HCl (2x), sat. aq. $NaHCO_3$ (2x) and brine (in case of morpholino acetic acid coupling, no 1 M HCl washing). The organic layer was dried over $MgSO_4$, filtered and concentrated in *vacuo*. Purification by silica gel flash column chromatography yielded the title compound.

Procedure C: Boc-deprotection

The appropriate Boc-protected amino acid derivative was dissolved in TFA:DCM 1:1 and stirred for 20 min. Co-evaporation with toluene (3x) and $CHCl_3$ (1x) afforded the TFA-salt, which was used without further purification unless stated otherwise.

Procedure D: Fmoc-deprotection

The appropriate Fmoc-protected amino acid derivative was dissolved in DMF (0.5 M) and N_3 (1.2 eq.) was added. The reaction mixture was stirred overnight at 50ºC followed by evaporation of the solvent under *vacuo*. Purification by silica gel flash column chromatography yielded the title compound.

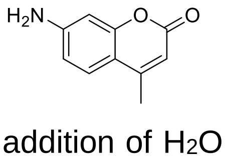

7-Amino-4-methylcoumarin (AMC, 15). m-Aminophenol (31.3 g, 287 mmol) and $NaHCO_3$ (35.0 g, 402 mmol) were dissolved in EtOAc (220 mL) and cooled to 0ºC. Methyl chloroformate (25.6 mL, 330 mmol) was added dropwise in the course of 1 h, then stirred 1 h, followed by the addition of H_2O (80 mL) and stirring for another 4 h. The aqueous layer was separated, and the organic layer was washed successively with H_2O, 1 M HCl, H_2O and brine. The organic layer was dried over $MgSO_4$, filtered and concentrated in *vacuo*. The crystalline residue was recrystallized from boiling toluene, filtered, rinsed with pentane and dried under *vacuo*, yielding intermediate *m*-(N-Methoxycarbonylamino)phenol.

Crude *m*-(N-Methoxycarbonylamino)phenol (41.8 g, 250 mmol) was mixed with ethyl acetoacetate (45 mL, 339 mmol) and slowly added to conc. H_2SO_4 (108 mL), after which the mixture was stirred for 2 h and diluted with a mixture of H_2O and ice (500 mL). A white precipitate was formed, which was filtered and rinsed with H_2O, MeOH, Et_2O and

pentane. The crude intermediate methoxycarbonylamino-4-methylcoumarin was dried under *vacuo*.

Then, it was dissolved in H_2O (63 mL) and KOH (45 g, 800 mmol) was added. The reaction mixture was stirred for exactly 20 min in a pre-heated bath at 90ºC, followed by cooling to 0ºC and the addition of cold H_2O (250 mL). Then, conc. HCl was added slowly until pH < 5, leading to crystallization of the product. The product was filtered over a glass filter, rinsed with H_2O and Et_2O followed by drying under *vacuo*. This yielded the tile compound as a yellow powder (18.8 g, 107 mmol, 37%). ^{1}H NMR (300 MHz, DMSO-d6) δ 7.40 (dd, J = 8.6, 2.1 Hz, 1H), 6.56 (dt, J = 8.6, 2.2 Hz, 1H), 6.40 (d, J = 2.2 Hz, 1H), 6.11 (s, 2H), 5.90 (t, J = 1.3 Hz, 1H), 2.30 (q, J = 1.2 Hz, 3H). ^{13}C NMR (75 MHz, D2O) δ 160.8, 155.5, 153.8, 153.1, 126.2, 111.2, 108.8, 107.5, 98.5, 18.0. LC-MS (linear gradient 10 → 90% MeCN/H_2O, 0.1% TFA, 12.5 min): R_t (min): 4.14 (ESI-MS (m/z): 176.13 (M+H$^+$)).

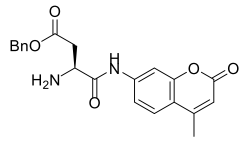

H$_2$N-Asp(Bn)-AMC (24). This compound was prepared by a peptide coupling according to procedure **A** on a 5.85 mmol scale using Boc-Asp(Bn)-OH, followed by deprotection using procedure **C**. This yielded the title compound as a brown syrup (1.88 g, 3.9 mmol, 80%). LC-MS (linear gradient 10 → 90% MeCN/H_2O, 0.1% TFA, 12.5 min): R_t (min): 5.01 (ESI-MS (m/z): 381.07 (M+H$^+$)). ^{1}H NMR (400 MHz, $CDCl_3$) δ 7.58 (d, J = 2.1 Hz, 1H), 7.52 (d, J = 8.6 Hz, 1H), 7.43 (dd, J = 8.6, 2.1 Hz, 1H), 7.35 (d, J = 2.6 Hz, 5H), 6.21 (d, J = 1.3 Hz, 1H), 5.88 (d, J = 8.5 Hz, 1H), 5.17 (d, J = 2.4 Hz, 2H), 4.68 (s, 1H), 3.08 (dd, J = 17.2, 4.4 Hz, 1H), 2.82 (dd, J = 17.1, 6.4 Hz, 1H), 2.41 (d, J = 1.3 Hz, 3H). ^{13}C NMR (101 MHz, $CDCl_3$) δ 172.0, 169.4, 161.1, 154.4, 152.2, 141.0, 135.3, 128.8, 128.7, 128.4, 125.3, 116.4, 115.8, 113.7, 107.5, 77.5, 77.2, 76.8, 67.3, 51.6.

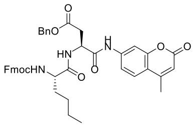

Fmoc-Nle-Asp(Bn)-AMC (25). This compound was prepared according to procedure **B** on a 3.9 mmol scale using Fmoc-Nle-OH. Purification by silica gel flash column chromatography (MeOH in DCM – 0% to 3% v/v) yielded the title compound (2.47 g, 3.32 mol, 85%). LC-MS (linear gradient 10 → 90% MeCN/H_2O, 0.1% TFA, 12.5 min): R_t (min): 8.89 (ESI-MS (m/z): 716.07 (M+H$^+$)).

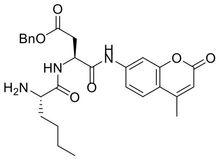

H$_2$N-Nle-Asp(Bn)-AMC (26). This compound was deprotected according to procedure **D** on a 3.3 mmol. Purification by silica gel flash column chromatography (MeOH in DCM – 0% to 8% v/v) yielded the title compound (1.3 g, 2.64 mmol, 80%). ^{1}H NMR (400 MHz, $CDCl_3$) δ 8.58 (d, J = 7.9 Hz, 1H), 7.66 (d, J = 2.1 Hz, 1H), 7.49 (d, J = 8.6 Hz, 1H), 7.40 – 7.30 (m, 6H), 6.20 (q, J = 1.2 Hz, 1H), 5.25 – 5.14 (m, 2H), 4.92 (q, J = 6.3 Hz, 1H), 3.45 (dd, J = 8.4, 4.2 Hz, 1H), 2.98 (dd, J = 16.8, 5.1 Hz, 1H), 2.89 (dd, J = 16.8, 6.6 Hz, 1H), 2.40 (d, J = 1.3 Hz, 3H), 1.93 – 1.80 (m, 1H), 1.56 – 1.46 (m, 1H),

1.35 (t, J = 3.2 Hz, 1H), 0.91 (q, J = 6.0, 5.3 Hz, 4H). LC-MS (linear gradient 10 → 90% MeCN/H_2O, 0.1% TFA, 12.5 min): R_t (min): 5.31 (ESI-MS (m/z): 493.22 (M+H$^+$)).

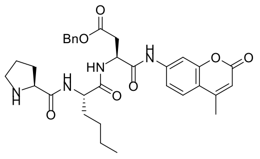

H_2N-Pro-Nle-Asp(Bn)-AMC (27) This compound was prepared according to procedure **B** on a 1.7 mmol scale using Boc-Pro-OH, followed by deprotection using procedure **C**. Purification by silica gel flash column chromatography (MeOH in DCM – 0% to 3% v/v) yielded the title compound (1.00 g, 1.7 mmol, quant.). ^{1}H NMR (400 MHz, $CDCl_3$) δ 7.98 – 7.85 (m, 2H), 7.72 (d, J = 8.8 Hz, 1H), 7.54 (d, J = 9.1 Hz, 1H), 7.29 (s, 5H), 6.22 (q, J = 1.1 Hz, 1H), 5.23 – 5.06 (m, 3H), 4.33 – 4.28 (m, 1H), 4.22 (dt, J = 9.0, 4.6 Hz, 1H), 3.48 (dt, J = 15.5, 7.0 Hz, 2H), 3.16 (dd, J = 17.2, 6.8 Hz, 1H), 3.06 – 2.96 (m, 1H), 2.83 (s, 3H), 2.44 (d, J = 1.2 Hz, 3H), 2.26 (dd, J = 12.5, 5.9 Hz, 1H), 2.14 (dt, J = 13.0, 8.1 Hz, 1H), 2.05 – 1.86 (m, 3H), 1.76 (dd, J = 8.9, 4.2 Hz, 1H), 1.43 – 1.22 (m, 2H), 0.93 (t, J = 6.8 Hz, 4H). ^{13}C NMR (101 MHz, $CDCl_3$) δ 171.5, 152.3, 128.6, 128.4, 128.2, 124.8, 116.1, 113.4, 107.7, 77.4, 77.0, 76.7, 66.9, 60.6, 55.5, 50.1, 38.6, 28.4, 27.8, 22.4, 18.6, 13.9. LC-MS (linear gradient 10 → 90% MeCN/H_2O, 0.1% TFA, 12.5 min): R_t (min): 5.70 (ESI-MS (m/z): 590.27 (M+H$^+$)). HRMS calculated for $C_{32}H_{36}N_4O_8$ 590.2740 [M+H]$^+$; found 590.2741

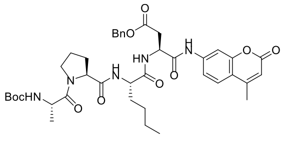

Boc-Ala-Pro-Nle-Asp(Bn)-AMC (28). This compound was prepared in a peptide coupling according to procedure **B** on a 1.7 mmol scale using Boc-Ala-OH. Purification by silica gel flash column chromatography (MeOH in DCM – 0% to 3% v/v) yielded the title compound (1.29 g, 1.7 mmol, quant.). ^{1}H NMR (400 MHz, $CDCl_3$) δ 9.00 (s, 1H), 7.89 – 7.82 (m, 2H), 7.74 (d, J = 8.6 Hz, 1H), 7.54 (t, J = 8.9 Hz, 1H), 7.40 – 7.31 (m, 5H), 6.21 (p, J = 1.2 Hz, 1H), 5.33 – 5.27 (m, 1H), 5.14 (d, J = 3.3 Hz, 1H), 5.05 (td, J = 7.6, 4.6 Hz, 1H), 4.57 – 4.49 (m, 1H), 4.43 (q, J = 7.2 Hz, 1H), 4.20 (dt, J = 10.4, 5.2 Hz, 1H), 3.81 (dt, J = 11.9, 6.2 Hz, 1H), 3.60 (td, J = 10.7, 10.1, 4.8 Hz, 1H), 3.22 (dd, J = 16.7, 7.2 Hz, 1H), 3.05 – 2.98 (m, 1H), 2.83 (s, 2H), 2.43 (dd, J = 3.1, 1.2 Hz, 3H), 2.30 – 2.00 (m, 2H), 1.97 – 1.66 (m, 1H), 1.46 (d, J = 4.7 Hz, 9H), 1.40 (s, 2H), 1.33 (dd, J = 19.4, 7.0 Hz, 9H), 0.92 (dt, J = 7.3, 3.8 Hz, 3H). ^{13}C NMR (101 MHz, $CDCl_3$) δ 174.0, 173.3, 171.8, 171.5, 169.3, 161.5, 154.2, 152.5, 141.8, 128.8, 128.7, 128.5, 128.3, 128.3, 125.0, 116.2, 113.4, 107.9, 107.8, 80.4, 67.0, 61.7, 61.1, 55.5, 50.6, 49.5, 49.0, 47.7, 38.8, 30.7, 28.4, 28.4, 28.3, 28.1, 25.7, 22.4, 22.2, 18.7, 17.8, 14.0, 13.9. LC-MS (linear gradient 10 → 90% MeCN/H_2O, 0.1% TFA, 12.5 min): R_t (min): 7.98 (ESI-MS (m/z): 762.13 (M+H$^+$)).

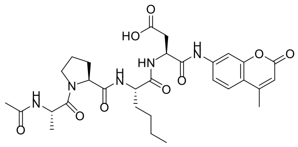

LU-FS11c, Ac-Ala-Pro-Nle-Asp-AMC (2). This compound was deprotected according to procedure **C**. This was followed treatment with Ac_2O (17 mmol, 1.6 mL), DiPEA (8.5 mmol, 1.5

mL) in DCM (50 mL) for 4 h at RT. Subsequently H_2O was added and the H_2O layer was extracted with DCM (3x) followed by concentration in *vacuo*. Purification by silica gel flash column chromatography (MeOH in DCM – 0% to 3% v/v) yielded intermediate Ac-Ala-Pro-Nle-Asp(Bn)-AMC (1.52 mmol, 90%). LC-MS (linear gradient 10 → 90% MeCN/H_2O, 0.1% TFA, 12.5 min): R_t (min): 6.77 (ESI-MS (m/z): 704.07 ($M+H^+$)). Ac-Ala-Pro-Nle-Asp(Bn)-AMC (1.07 g, 1.53 mmol) was debenzylated by dissolving it in MeOH (5 mL) followed by the addition of 10% Pd/C (50 mg). The reaction mixture was flushed with N_2 and put under an H_2 atmosphere. After 30 min the mixture was filtered over Celite, rinsed with MeOH and concentrated under *vacuo*. Purification by silica gel flash column chromatography (MeOH in DCM – 0% to 3% v/v) yielded the title compound (0.63 g, 1.04 mmol, 68%). ^{1}H NMR (500 MHz, DMSO-d6) δ 10.22 (s, 1H), 8.26 (d, J = 7.6 Hz, 1H), 8.15 (d, J = 7.4 Hz, 1H), 8.10 (d, J = 6.6 Hz, 1H), 7.78 (dd, J = 6.4, 2.0 Hz, 1H), 7.71 (d, J = 8.7 Hz, 1H), 7.60 (dd, J = 8.7, 2.0 Hz, 1H), 6.26 (d, J = 1.4 Hz, 1H), 4.67 – 4.60 (m, 1H), 4.53 (p, J = 7.1 Hz, 1H), 4.35 (dd, J = 8.4, 3.6 Hz, 1H), 4.14 – 4.05 (m, 1H), 3.64 – 3.58 (m, 1H), 3.54 (dd, J = 9.5, 6.9 Hz, 1H), 2.76 (dd, J = 16.6, 5.9 Hz, 1H), 2.69 – 2.61 (m, 1H), 2.39 (d, J = 1.3 Hz, 3H), 2.10 – 2.00 (m, 1H), 1.85 (dt, J = 11.3, 8.2 Hz, 2H), 1.81 (s, 3H), 1.71 – 1.62 (m, 1H), 1.56 (dd, J = 8.7, 4.7 Hz, 1H), 1.31 – 1.22 (m, 7H), 1.19 (d, J = 7.0 Hz, 3H), 1.10 (s, 2H), 0.86 – 0.80 (m, 3H). ^{13}C NMR (126 MHz, DMSO) δ 172.4, 171.7, 171.3, 170.3, 168.9, 160.0, 153.6, 153.2, 142.2, 125.9, 115.3, 115.2, 112.4, 105.8, 59.6, 53.4, 50.8, 46.8, 46.1, 31.3, 31.1, 28.9, 27.3, 24.6, 22.3, 21.9, 21.9, 18.0, 17.0, 13.9, 13.8. LC-MS (linear gradient 10 → 90% MeCN/H_2O, 0.1% TFA, 12.5 min): R_t (min): 5.16 (ESI-MS (m/z): 613.87 ($M+H^+$)). HRMS calculated for $C_{30}H_{40}N_5O_9$ 614.28205 $[M+H]^+$; found 614.28172.

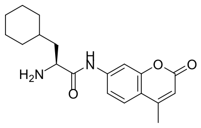

H_2N-Cha-AMC (29). This compound was prepared by a peptide coupling according to procedure **A** on a 5.85 mmol scale using Boc-Cha-OH, followed by deprotection using procedure **C**. This yielded the title compound as a brown syrup (1.92 g, 5.85 mmol, quant.). ^{1}H NMR (500 MHz, MeOD) δ 7.82 (d, J = 2.1 Hz, 1H), 7.67 (d, J = 8.7 Hz, 1H), 7.46 (dd, J = 8.7, 2.1 Hz, 1H), 6.20 (q, J = 1.2 Hz, 1H), 4.13 (dd, J = 8.8, 5.7 Hz, 1H), 2.42 (d, J = 1.3 Hz, 3H), 1.91 – 1.64 (m, 7H), 1.47 (s, 4H), 1.35 – 1.16 (m, 4H), 1.03 (dtd, J = 14.1, 10.4, 2.2 Hz, 2H). ^{13}C NMR (126 MHz, MeOD) δ 168.51, 161.74, 153.92, 153.90, 153.78, 141.39, 125.53, 116.28, 115.77, 112.61, 106.87, 106.64, 51.91, 38.98, 33.42, 33.33, 32.00, 31.98, 27.48, 25.96, 25.87, 25.59, 17.24. LC-MS (linear gradient 10 → 90% MeCN/H_2O, 0.1% TFA, 12.5 min): R_t (min): 8.31 (ESI-MS (m/z): 329.27 ($M+H^+$)).

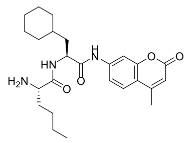

H_2N-Nle-Cha-AMC (30). This compound was prepared according to procedure **B** on a 2.3 mmol scale using Fmoc-Nle-OH, followed by deprotection using procedure **D**. Purification by silica gel flash column chromatography (MeOH in DCM – 0% to 3% v/v) yielded the title

compound (0.84 g, 1.9 mmol, 81%). LC-MS (linear gradient 10 → 90% MeCN/H_2O, 0.1% TFA, 12.5 min): R_t (min): 8.21 (ESI-MS (m/z): 442.26 (M+H^+)).

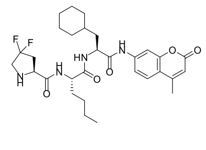

H_2N-F_2Pro-Nle-Cha-AMC (31). This compound was prepared according to procedure **B** on a 1.2 mmol scale using Boc-F_2Pro-OH, followed by deprotection using procedure **C**. Purification by silica gel flash column chromatography (MeOH in DCM – 0% to 3% v/v) yielded the title compound (0.81 g, 1.2 mmol, quant.). LC-MS (linear gradient 10 → 90% MeCN/H_2O, 0.1% TFA, 12.5 min): R_t (min): 8.34 (ESI-MS (m/z): 674.93 (M+H^+)).

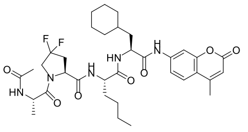

LU-FS11i, Ac-Ala-F_2Pro-Nle-Cha-AMC (4). This compound was prepared in a peptide coupling according to procedure **B** on a 1.2 mmol scale using Boc-Ala-OH. Subsequently, it was deprotected according to procedure **C**. This was followed treatment with Ac_2O (2.5 mL, 21 mmol), DiPEA (7.3 mmol, 1.25 mL) in DCM (10 mL) for 4 h at RT. Then, H_2O was added and the H_2O layer was extracted with DCM (3x) followed by concentration in *vacuo*. Purification by silica gel flash column chromatography (MeOH in DCM – 0% to 3% v/v) yielded the title compound (0.577 g, 0.84 mmol, 70%). ^1H NMR (500 MHz, MeOD) δ 7.98 (s, 2H), 7.90 (s, 2H), 7.86 (dd, J = 7.2, 2.0 Hz, 1H), 7.65 (dd, J = 8.7, 2.6 Hz, 1H), 7.59 (ddd, J = 8.7, 5.1, 2.1 Hz, 1H), 6.22 – 6.18 (m, 1H), 4.54 (ddd, J = 22.7, 10.6, 4.6 Hz, 1H), 4.32 (dp, J = 13.5, 6.5 Hz, 1H), 4.24 – 4.08 (m, 1H), 2.99 (s, 5H), 2.86 (d, J = 0.7 Hz, 6H), 2.64 – 2.51 (m, 1H), 2.47 – 2.41 (m, 3H), 1.99 (d, J = 12.4 Hz, 3H), 1.87 – 1.72 (m, 2H), 1.73 (s, 1H), 1.72 (d, J = 3.3 Hz, 1H), 1.67 (ddd, J = 14.0, 10.9, 4.9 Hz, 1H), 1.44 – 1.31 (m, 4H), 1.31 – 1.15 (m, 3H), 1.09 – 0.88 (m, 2H). ^{13}C NMR (126 MHz, MeOD) δ 175.82, 174.87, 174.85, 173.55, 173.51, 173.37, 173.34, 173.19, 173.13, 172.50, 164.83, 163.20, 163.16, 155.27, 155.20, 155.18, 155.17, 143.35, 143.25, 127.55, 126.64, 126.57, 126.51, 117.42, 117.27, 117.20, 117.18, 117.10, 117.05, 113.70, 113.61, 113.59, 108.18, 108.03, 107.93, 60.43, 56.39, 54.85, 54.59, 53.45, 53.31, 53.15, 51.09, 49.23, 46.99, 39.97, 39.91, 38.04, 37.85, 37.65, 36.95, 35.66, 35.37, 35.11, 34.98, 34.85, 33.26, 33.00, 32.88, 32.45, 31.72, 31.64, 29.28, 29.06, 27.53, 27.44, 27.39, 27.23, 27.17, 23.40, 23.28, 22.41, 22.39, 21.96, 19.32, 18.54, 17.58, 16.99, 16.17, 15.50, 14.34, 14.25. LC-MS (linear gradient 10 → 90% MeCN/H_2O, 0.1% TFA, 12.5 min): R_t (min): 8.16 (ESI-MS (m/z): 688.07 (M+H^+)). HRMS calculated for $C_{35}H_{48}F_2N_5O_7$ 688.35218 $[M+H]^+$; found 688.35178.

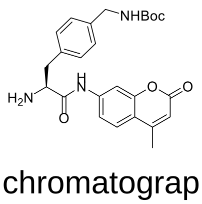

H_2N-Phe(Boc-4-aminomethyl)-AMC (32). This compound was prepared by a peptide coupling according to procedure **A** on a 1.00 mmol scale using Fmoc-Phe(Boc-4-aminomethyl)-OH, followed by deprotection using procedure **D**. Purification by silica gel flash column chromatography (MeOH in DCM – 0% to 3% v/v) yielded the title compound (0.38 g, 0.82 mmol, 82%). LC-MS (linear gradient 10 → 90% MeCN/H_2O, 0.1% TFA, 12.5 min): R_t (min): 8.98 (ESI-MS (m/z): 574.20 (M+H^+)). ^1H NMR (300 MHz, MeOD) δ 7.73 (d, J =

2.1 Hz, 1H), 7.65 (dd, J = 8.9, 2.0 Hz, 1H), 7.39 (dd, J = 8.7, 2.1 Hz, 1H), 7.19 (d, J = 1.6 Hz, 4H), 6.22 – 6.18 (m, 1H), 5.49 (d, J = 1.6 Hz, 1H), 4.16 (s, 2H), 3.70 (t, J = 6.8 Hz, 1H), 3.06 (dd, J = 13.4, 6.6 Hz, 1H), 2.96 – 2.83 (m, 1H), 2.42 (s, 3H), 1.42 (s, 9H). ^{13}C NMR (75 MHz, MeOD) δ 175.5, 163.2, 155.2, 143.1, 137.3, 130.5, 128.4, 126.7, 117.2, 117.2, 113.6, 108.0, 58.4, 44.7, 42.2, 28.8, 18.5.

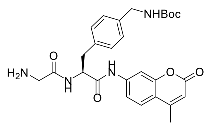

H_2N-Gly-Phe(Boc-4-aminomethyl)-AMC (33). This compound was prepared according to procedure **B** on a 0.82 mmol scale using Fmoc-Gly-OH, followed by deprotection using procedure **D**. Purification by silica gel flash column chromatography (MeOH in DCM – 0% to 5% v/v) yielded the title compound (0.34 g, 0.68 mmol, 83%). LC-MS (linear gradient 10 → 90% MeCN/H_2O, 0.1% TFA, 12.5 min): R_t (min): 5.50 (ESI-MS (m/z): 508.73 (M+H$^+$)).

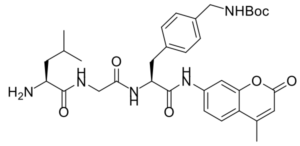

H_2N-Leu-Gly-Phe(Boc-4-aminomethyl)-AMC (34). This compound was prepared according to procedure **B** on a 0.68 mmol scale using Fmoc-Leu-OH, followed by deprotection using procedure **D**. Purification by silica gel flash column chromatography (MeOH in DCM – 0% to 5% v/v) yielded the title compound (0.24g, 0.38 mmol, 56%). LC-MS (linear gradient 10 → 90% MeCN/H_2O, 0.1% TFA, 12.5 min): R_t (min): 5.85 (ESI-MS (m/z): 622.13 (M+H$^+$)).

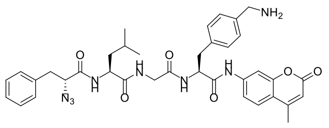

LU-FS12c, N_3Phe-Leu-Gly-Phe(Boc-4-aminomethyl)-AMC (6). This compound was prepared according to procedure **B** on a 0.15 mmol scale using N_3-Phe-OH , followed by deprotection using procedure **C**. Purification by silica gel flash column chromatography (MeOH in DCM – 0% to 3% v/v) yielded the title compound (12.6 mg, 0.018 mmol, 12%). ^{1}H NMR (500 MHz, MeOD) δ 7.78 (d, J = 2.0 Hz, 1H), 7.70 (d, J = 8.7 Hz, 1H), 7.52 (dd, J = 8.7, 2.1 Hz, 1H), 7.43 – 7.35 (m, 4H), 7.33 – 7.25 (m, 2H), 7.28 – 7.19 (m, 3H), 6.25 (t, J = 1.3 Hz, 1H), 4.77 (dd, J = 8.5, 6.3 Hz, 1H), 4.35 (dd, J = 8.1, 6.6 Hz, 1H), 4.15 – 4.08 (m, 2H), 4.07 (s, 2H), 3.92 (d, J = 16.7 Hz, 1H), 3.77 – 3.70 (m, 1H), 3.36 (s, 3H), 3.35 – 3.28 (m, 1H), 3.28 – 3.17 (m, 1H), 3.11 (dd, J = 13.8, 8.5 Hz, 1H), 2.98 (dd, J = 14.1, 8.7 Hz, 1H), 2.46 (d, J = 1.3 Hz, 3H), 2.12 – 2.05 (m, 1H), 2.03 (s, 1H), 1.93 – 1.86 (m, 1H), 1.66 – 1.54 (m, 3H), 1.44 – 1.21 (m, 6H), 1.02 – 0.87 (m, 6H). LC-MS (linear gradient 10 → 90% MeCN/H_2O, 0.1% TFA, 12.5 min): R_t (min): 8.49 (ESI-MS (m/z): 695.13 (M+H$^+$)). HRMS calculated for $C_{37}H_{43}N_8O_6$ 695.33001 [M+H]$^+$; found 695.32951.

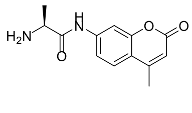

H_2N-Ala-AMC (35). This compound was prepared by a peptide coupling according to procedure **A** on a 5.85 mmol scale using Boc-Ala-OH, followed by deprotection using procedure **C**. Purification by silica gel flash column chromatography (MeOH in DCM – 0% to 3% v/v) yielded the title

compound (0.454 g, 0.78 mmol, 75%). ^{1}H NMR (400 MHz, $CDCl_3$) δ 9.50 (s, 1H), 7.73 (d, J = 1.9 Hz, 1H), 7.40 (d, J = 8.6 Hz, 1H), 7.11 (d, J = 8.6 Hz, 1H), 6.08 (s, 1H), 5.45 (d, J = 7.0 Hz, 1H), 4.47 (d, J = 10.0 Hz, 1H), 2.36 (s, 3H), 2.17 (s, 1H), 1.46 (d, J = 7.0 Hz, 3H). ^{13}C NMR (101 MHz, CDCl3) δ 172.1, 161.2, 153.9, 152.6, 141.5, 124.9, 115.6, 115.4, 113.0, 107.0, 80.9, 51.1, 18.5, 17.7. LC-MS (linear gradient 10 → 90% MeCN/H_2O, 0.1% TFA, 12.5 min): R_t (min): 3.40 (ESI-MS (m/z): 247.07 (M+H$^+$)).

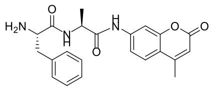

H$_2$N-Phe-Ala-AMC (36). This compound was prepared according to procedure **B** on a 5.3 mmol scale using Boc-Phe-OH, followed by deprotection using procedure **C**. Purification by silica gel flash column chromatography (MeOH in DCM – 0% to 3% v/v) yielded the title compound (1.884 g, 3.71 mmol, 70%). ^{1}H NMR (400 MHz, MeOD) δ 7.84 (d, J = 2.0 Hz, 1H), 7.75 – 7.69 (m, 1H), 7.47 (dd, J = 8.7, 2.1 Hz, 1H), 7.33 – 7.29 (m, 4H), 6.25 (d, J = 1.3 Hz, 1H), 4.56 (q, J = 7.1 Hz, 1H), 4.16 (dd, J = 8.5, 5.6 Hz, 1H), 3.05 (dd, J = 14.3, 8.5 Hz, 1H), 2.47 (d, J = 1.3 Hz, 4H), 1.48 (d, J = 7.1 Hz, 4H). ^{13}C NMR (101 MHz, MeOD) δ 172.9, 169.5, 163.2, 155.4, 155.3, 143.4, 135.5, 130.6, 130.1, 128.8, 127.1, 126.8, 120.9, 117.3, 117.0, 113.7, 110.7, 107.9, 55.5, 51.3, 38.6, 18.5, 18.1. LC-MS (linear gradient 10 → 90% MeCN/H_2O, 0.1% TFA, 12.5 min): R_t (min): 7.09 (ESI-MS (m/z): 393.93 (M+H$^+$)).

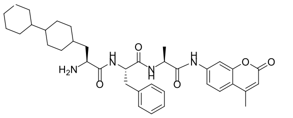

H$_2$N-BiCha-Phe-Ala-AMC (37). This compound was prepared according to procedure **B** on a 0.96 mmol scale using Boc-BiCha-OH, followed by deprotection using procedure **C**. Purification by silica gel flash column chromatography (MeOH in DCM – 0% to 3% v/v) yielded the title compound (0.805 g, 0.95 mmol, 99%). ^{1}H NMR (500 MHz, MeOD) δ 7.87 – 7.81 (m, 1H), 7.76 – 7.68 (m, 1H), 7.64 – 7.46 (m, 1H), 7.37 – 7.12 (m, 5H), 6.24 (d, J = 2.1 Hz, 1H), 4.72 – 4.63 (m, 1H), 4.63 – 4.52 (m, 1H), 4.52 – 4.38 (m, 1H), 4.36 – 4.18 (m, 1H), 4.15 – 4.07 (m, 1H), 3.25 – 3.14 (m, 1H), 2.50 – 2.41 (d, 3H), 1.69 (d, J = 38.9 Hz, 12H), 1.58 – 1.06 (m, 8H), 1.00 – 0.71 (m, 4H). LC-MS (linear gradient 10 → 90% MeCN/H_2O, 0.1% TFA, 12.5 min): R_t (min): 7.08 (ESI-MS (m/z): 629.20 (M+H$^+$)).

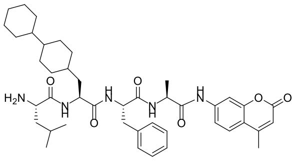

H$_2$N-Leu-BiCha-Phe-Ala-AMC (38). This compound was prepared according to procedure **B** on a 0.078 mmol scale using Boc-Leu-OH, followed by deprotection using procedure **C**. Purification by silica gel flash column chromatography (MeOH in DCM – 0% to 3% v/v) yielded the title compound (33 mg, 0.07 mmol, 90%). LC-MS (linear gradient 10 → 90% MeCN/H_2O, 0.1% TFA, 12.5 min): R_t (min): 7.51 (ESI-MS (m/z): 472.33 (M+H$^+$)).

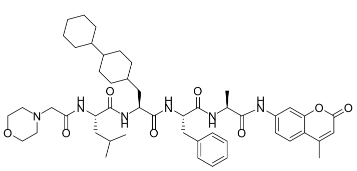

LU-FS15c, Morph-Leu-BiCha-Phe-Ala-AMC (17). This compound was prepared according to procedure **B** on a 0.101

mmol scale using Moph-OH. Purification by silica gel flash column chromatography (MeOH in DCM – 0% to 3% v/v) was followed by Prep-HPLC purification. After freezedrying this yielded the title compound (7.76 mg, 89 µmol, 9%). ^{1}H NMR (500 MHz, MeOD) δ 7.91 (s, 1H), 7.86 (d, J = 2.1 Hz, 1H), 7.73 (d, J = 8.7 Hz, 1H), 7.55 (ddd, J = 14.6, 8.4, 1.6 Hz, 2H), 7.38 (ddd, J = 8.3, 7.3, 1.2 Hz, 1H), 7.30 (dd, J = 7.6, 1.2 Hz, 1H), 7.30 – 7.21 (m, 4H), 7.25 – 7.14 (m, 1H), 6.25 (t, J = 1.3 Hz, 1H), 4.55 (dd, J = 8.9, 5.5 Hz, 1H), 4.48 – 4.39 (m, 2H), 4.29 (dd, J = 9.8, 5.1 Hz, 1H), 4.16 – 4.05 (m, 1H), 3.90 – 3.84 (m, 1H), 3.79 (s, 4H), 3.76 – 3.70 (m, 1H), 3.46 (s, 1H), 3.24 – 3.20 (m, 2H), 3.19 – 3.12 (m, 4H), 3.01 (dd, J = 14.0, 8.9 Hz, 1H), 2.84 (s, 4H), 2.70 (s, 1H), 2.47 (d, J = 1.3 Hz, 3H), 1.83 – 1.67 (m, 3H), 1.72 (s, 7H), 1.67 – 1.46 (m, 3H), 1.44 (d, J = 7.1 Hz, 3H), 1.41 (d, J = 9.8 Hz, 1H), 1.34 (d, J = 24.7 Hz, 4H), 1.29 (s, 3H), 1.22 (s, 3H), 1.20 – 1.10 (m, 2H), 1.08 (d, J = 8.8 Hz, 1H), 0.92 (dd, J = 11.0, 6.5 Hz, 5H). LC-MS (linear gradient 10 → 90% MeCN/H_2O, 0.1% TFA, 12.5 min): R_t (min): 7.74 (ESI-MS (m/z): 869.47 (M+H$^+$)). HRMS calculated for $C_{49}H_{69}N_6O_8$ 869.51714 [M+H]$^+$; found 869.51621.

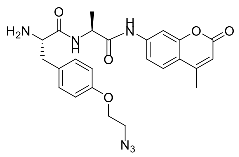

H_2N-Tyr(N_3)-Ala-AMC (39). This compound was prepared according to procedure **B** on a 1.0 mmol scale using Boc-N_3Tyr-OH, followed by deprotection using procedure **C**. Purification by silica gel flash column chromatography (MeOH in DCM – 0% to 3% v/v) yielded the title compound (0.454 g, 0.78 mmol, 75%). ^{1}H NMR (400 MHz, MeOD) δ 7.76 (d, J = 2.0 Hz, 1H), 7.65 (s, 3H), 7.48 (d, J = 8.7 Hz, 1H), 7.31 (dt, J = 9.0, 2.2 Hz, 1H), 7.22 – 7.17 (m, 2H), 7.15 – 7.10 (m, 2H), 7.09 – 7.03 (m, 4H), 6.77 – 6.71 (m, 2H), 6.08 (t, J = 1.4 Hz, 1H), 4.57 (q, J = 7.1 Hz, 1H), 4.27 (d, J = 23.8 Hz, 1H), 4.21 (d, J = 6.6 Hz, 1H), 3.96 – 3.86 (m, 2H), 3.66 – 3.57 (m, 1H), 3.41 (dd, J = 5.9, 4.0 Hz, 2H), 3.23 (dd, J = 14.2, 6.3 Hz, 1H), 3.07 (dd, J = 14.1, 7.3 Hz, 1H), 2.75 (s, 2H), 2.31 (d, J = 1.3 Hz, 4H), 2.24 (s, 4H), 1.46 (dd, J = 7.1, 3.5 Hz, 4H), 1.16 (t, J = 7.1 Hz, 1H). ^{13}C NMR (101 MHz, MeOD) δ 172.6, 169.6, 163.2, 161.0, 160.7, 159.1, 158.3, 155.0, 154.9, 143.1, 143.0, 138.7, 131.9, 131.7, 131.6, 129.8, 129.0, 128.3, 127.8, 127.5, 126.4, 126.1, 118.2, 117.0, 116.9, 115.9, 115.7, 115.4, 113.5, 107.9, 79.0, 68.1, 68.0, 65.0, 58.3, 55.5, 51.4, 51.0, 44.1, 38.8, 37.5, 21.5, 18.6, 18.2, 17.9. LC-MS (linear gradient 10 → 90% MeCN/H_2O, 0.1% TFA, 12.5 min): R_t (min): 7.65 (ESI-MS (m/z): 479.13 (M+H$^+$)).

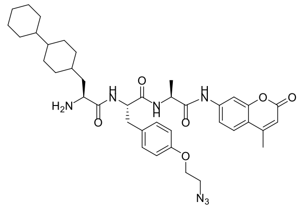

H_2N-BiCha-Tyr(N_3)-Ala-AMC (40). This compound was prepared according to procedure **B** on a 0.78 mmol scale using Boc-BiCha-OH, followed by deprotection using procedure **C**. Purification by silica gel flash column chromatography (MeOH in DCM – 0% to 3% v/v) yielded the title compound (0.103 g, 0.15 mmol, 19%). ^{1}H NMR (400 MHz, $CDCl_3$) δ 8.01 – 7.91 (m, 1H), 7.69 – 7.62 (m, 1H), 7.54 – 7.42 (m, 2H), 7.22 – 7.02 (m, 4H), 6.89 (dd, J = 8.8, 2.6 Hz, 1H), 6.19 – 6.12 (m, 2H), 4.80 – 4.63 (m, 2H), 4.58 – 4.47 (m, 1H), 4.12 (q, J = 6.1, 5.5

Hz, 1H), 4.06 (dq, J = 15.0, 4.9 Hz, 1H), 3.61 (dd, J = 5.6, 4.2 Hz, 1H), 3.56 (q, J = 5.6, 4.9 Hz, 1H), 3.20 (dd, J = 6.5, 4.2 Hz, 1H), 2.42 – 2.37 (m, 4H), 1.78 – 1.59 (m, 14H), 1.55 – 1.39 (m, 16H), 1.25 – 1.05 (m, 6H), 1.03 – 0.74 (m, 2H). ^{13}C NMR (101 MHz, CDCl3) δ 173.2, 170.9, 170.4, 161.3, 157.8, 154.2, 153.3, 152.2, 141.9, 133.4, 130.4, 130.3, 128.4, 125.0, 124.7, 116.2, 116.0, 115.9, 115.3, 114.9, 113.3, 107.6, 84.3, 83.9, 77.4, 67.1, 57.6, 55.6, 50.2, 49.6, 43.2, 41.8, 38.7, 35.8, 31.3, 30.6, 30.3, 29.8, 29.5, 28.6, 28.3, 28.0, 27.9, 27.9, 26.9, 26.8, 25.5, 18.6, 17.3. LC-MS (linear gradient 10 → 90% MeCN/H_2O, 0.1% TFA, 12.5 min): R_t (min): 10.88 (ESI-MS (m/z): 714.27 ($M+H^+$)).

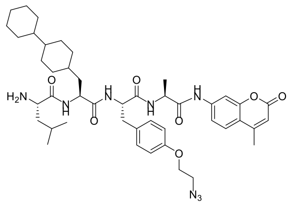

H_2N-Leu-BiCha-Tyr(N_3)-Ala-AMC (41) This compound was prepared according to procedure **B** on a 0.125 mmol scale using Boc-Leu-OH, followed by deprotection using procedure **C**. Purification by silica gel flash column chromatography (MeOH in DCM – 0% to 3% v/v) yielded the title compound (0.024 g, 0.026 mmol, 21%). LC-MS (linear gradient 10 → 90% MeCN/H_2O, 0.1% TFA, 12.5 min): R_t (min): 10.77 (ESI-MS (m/z): 827.33 ($M+H^+$)).

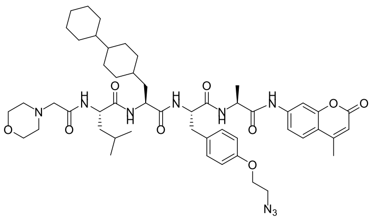

LU-FS25i, Morph-Leu-BiCha-Tyr(N_3)-Ala-AMC (18). This compound was prepared according to procedure **B** on a 0.026 mmol scale using Morph-OH. Purification by silica gel flash column chromatography (MeOH in DCM – 0% to 3% v/v) was followed by prep-HPLC purification. After freezedrying this yielded the title compound (7.76 mg, 0.0089 mmol, 9%). ^{1}H NMR (400 MHz, DMSO-d6) δ 8.26 – 7.87 (m, 1H), 7.80 (t, J = 2.2 Hz, 1H), 7.71 (dd, J = 8.8, 1.6 Hz, 1H), 7.51 (ddt, J = 13.3, 8.6, 2.2 Hz, 1H), 7.16 (dd, J = 13.2, 8.5 Hz, 2H), 6.86 – 6.80 (m, 1H), 6.80 – 6.72 (m, 1H), 6.29 – 6.21 (m, 1H), 4.54 – 4.34 (m, 1H), 4.17 – 4.09 (m, 1H), 4.06 (s, 8H), 3.64 – 3.55 (m, 6H), 3.16 (s, 23H), 3.07 – 2.92 (m, 1H), 2.68 (s, 10H), 2.45 – 2.35 (m, 7H), 1.62 (dd, J = 31.1, 10.7 Hz, 7H), 1.46 – 1.36 (m, 1H), 1.36 – 1.30 (m, 2H), 1.30 – 1.20 (m, 5H), 1.18 – 0.96 (m, 3H), 0.92 – 0.68 (m, 9H). ^{13}C NMR (101 MHz, DMSO) δ 160.2, 156.8, 153.9, 153.2, 142.3, 130.5, 130.1, 126.0, 115.3, 115.3, 114.2, 112.5, 105.9, 66.9, 66.2, 53.8, 53.3, 49.8, 48.7, 48.6, 47.7, 43.0, 42.0, 41.2, 30.2, 29.9, 26.5, 26.4, 24.5, 23.1, 21.7, 21.7, 18.0, 17.8. LC-MS (linear gradient 10 → 90% MeCN/H_2O, 0.1% TFA, 12.5 min): R_t (min): 8.02 (ESI-MS (m/z): 954.60 ($M+H^+$)). HRMS calculated for $C_{51}H_{72}N_9O_9$ 954.54475 $[M+H]^+$; found 954.54399.

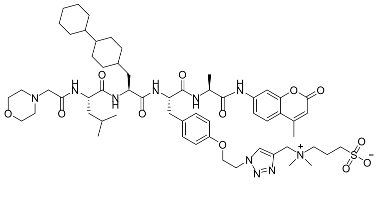

LU-FS35i, Morph-Leu-BiCha-Tyr(Solubilizer)-Ala-AMC (19). A solution of Morph-Leu-BiCha-Tyr(N_3)-Ala-AMC (5.7 µmol) in DMF (5 mL) was purged with N_2. Then, sodium ascobate (2.3 mg), $CuSO_4$ (7.8 mg) and alkyne linker (4.4

mg) were added. After stirring overnight at RT the solvent was evaporated. Purification by silica gel flash column chromatography (MeOH in DCM – 0% to 3% v/v) was followed by Prep-HPLC purification. After freezedrying this yielded the title compound (6.07 µg, 5.2 µmol, 91%). LC-MS (linear gradient 10 → 90% MeCN/H_2O, 0.1% TFA, 12.5 min): R_t (min): 6.46 (ESI-MS (m/z): 1159.60 ($M+H^+$)). HRMS calculated for $C_{59}H_{86}N_{10}O_{12}S$ 1159.62202 $[M+H]^+$; found 1159.62215.

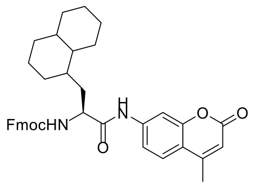

Fmoc-1-DecAla-AMC (42). This compound was prepared by a peptide coupling according to procedure **A** on a 0.27 mmol scale using Fmoc-1DecAla-OH. Purification by silica gel flash column chromatography (MeOH in DCM – 0% to 3% v/v) yielded the title compound (0.119 g, 0.20 mmol, 73%). LC-MS (linear gradient 10 → 90% MeCN/H_2O, 0.1% TFA, 12.5 min): R_t (min): 10.80 (ESI-MS (m/z): 605.13 ($M+H^+$)).

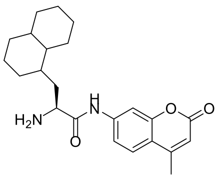

H_2N-1-DecAla-AMC (43). This compound was deprotected according to procedure **D** on a 0.20 mmol. Purification by silica gel flash column chromatography (MeOH in DCM – 0% to 8% v/v) yielded the title compound (0.067 g, 0.18 mol, 88%). LC-MS (linear gradient 10 → 90% MeCN/H_2O, 0.1% TFA, 12.5 min): R_t (min): 6.03 (ESI-MS (m/z): 383.20 ($M+H^+$)).

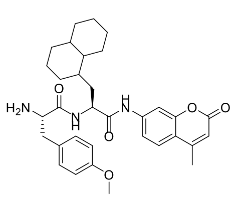

H_2N-Tyr(Me)-1-DecAla-AMC (44). This compound was prepared according to procedure **B** on a 0.43 mmol scale using Boc-Tyr(Me)-OH, followed by deprotection using procedure **C**. Purification by silica gel flash column chromatography (MeOH in DCM – 0% to 3% v/v) yielded the title compound (0.236 g, 0.36 mmol, 84%). ^1H NMR (400 MHz, MeOD) δ 7.88 (dd, J = 2.9, 2.1 Hz, 1H), 7.85 – 7.72 (m, 2H), 7.53 (ddd, J = 8.7, 3.7, 2.1 Hz, 1H), 7.24 – 7.14 (m, 2H), 6.83 – 6.74 (m, 2H), 6.68 (dd, J = 8.7, 2.2 Hz, 1H), 6.54 (d, J = 2.2 Hz, 1H), 6.30 (dd, J = 2.7, 1.4 Hz, 1H), 4.59 (dt, J = 8.2, 5.7 Hz, 1H), 4.13 (dt, J = 10.0, 8.2 Hz, 1H), 3.80 (d, J = 10.4 Hz, 1H), 3.73 – 3.64 (m, 3H), 3.31 – 3.11 (m, 1H), 3.07 – 3.00 (m, 1H), 2.84 (s, 2H), 2.50 (dt, J = 7.0, 1.6 Hz, 5H), 1.91 – 1.18 (m, 12H). LC-MS (linear gradient 10 → 90% MeCN/H_2O, 0.1% TFA, 12.5 min): R_t (min): 9.36 (ESI-MS (m/z): 560.13 ($M+H^+$)).

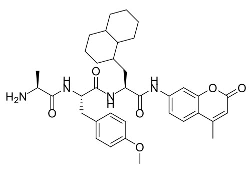

H_2N-Ala-Tyr(Me)-1-DecAla-AMC (45). This compound was prepared according to procedure **B** on a 0.35 mmol scale using Boc-Ala-OH, followed by deprotection using procedure **C**. Purification by silica gel flash column chromatography (MeOH in DCM – 0% to 3%

v/v) yielded the title compound (0.337 g, 0.35 mmol, quant.). LC-MS (linear gradient 10 → 90% MeCN/H_2O, 0.1% TFA, 12.5 min): R_t (min): 10.08 (ESI-MS (m/z): 631.13 ($M+H^+$))

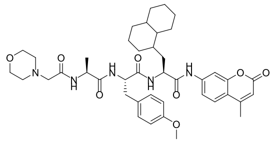

LU-FS12i, Morph-Ala-Tyr(Me)-1-DecAla-AMC (7). This compound was prepared according to procedure **B** on a 0.04 mmol scale using Morph-OH. Purification by silica gel flash column chromatography (MeOH in DCM – 0% to 3% v/v) yielded the title compound (16.56 mg, 0.022 mmol, 55%). ^{1}H NMR (400 MHz, $CDCl_3$) δ 9.40 (s, 1H), 7.87 (ddd, J = 8.6, 6.1, 2.1 Hz, 1H), 7.79 (dd, J = 3.5, 2.0 Hz, 1H), 7.74 – 7.61 (m, 1H), 7.54 (dd, J = 8.7, 3.1 Hz, 1H), 7.10 – 6.96 (m, 3H), 6.77 – 6.65 (m, 2H), 6.23 – 6.15 (m, 1H), 4.77 – 4.58 (m, 1H), 4.51 (s, 1H), 3.82 – 3.59 (m, 8H), 3.23 – 2.87 (m, 3H), 2.55 – 2.45 (m, 1H), 2.45 – 2.38 (m, 3H), 2.12 – 1.70 (m, 1H), 1.66 (d, J = 12.7 Hz, 2H), 1.59 – 1.08 (m, 9H). ^{13}C NMR (101 MHz, $CDCl_3$) δ 172.9, 171.6, 171.4, 161.4, 158.8, 154.2, 152.6, 142.1, 130.5, 127.7, 125.2, 116.2, 116.1, 114.2, 113.3, 107.6, 67.1, 66.9, 61.6, 55.4, 55.2, 55.2, 54.8, 53.9, 53.8, 52.9, 52.5, 49.9, 43.4, 41.3, 38.7, 38.1, 37.9, 37.6, 32.8, 26.8, 26.8, 25.6, 21.4, 20.2, 18.7, 17.4, 12.6. LC-MS (linear gradient 10 → 90% MeCN/H_2O, 0.1% TFA, 12.5 min): R_t (min): 7.07 (ESI-MS (m/z): 758.27 ($M+H^+$)). HRMS calculated for $C_{42}H_{56}N_5O_8$ 758.41234 $[M+H]^+$; found 758.41152.

NMR Spectra

^{1}H-NMR ACC (23)

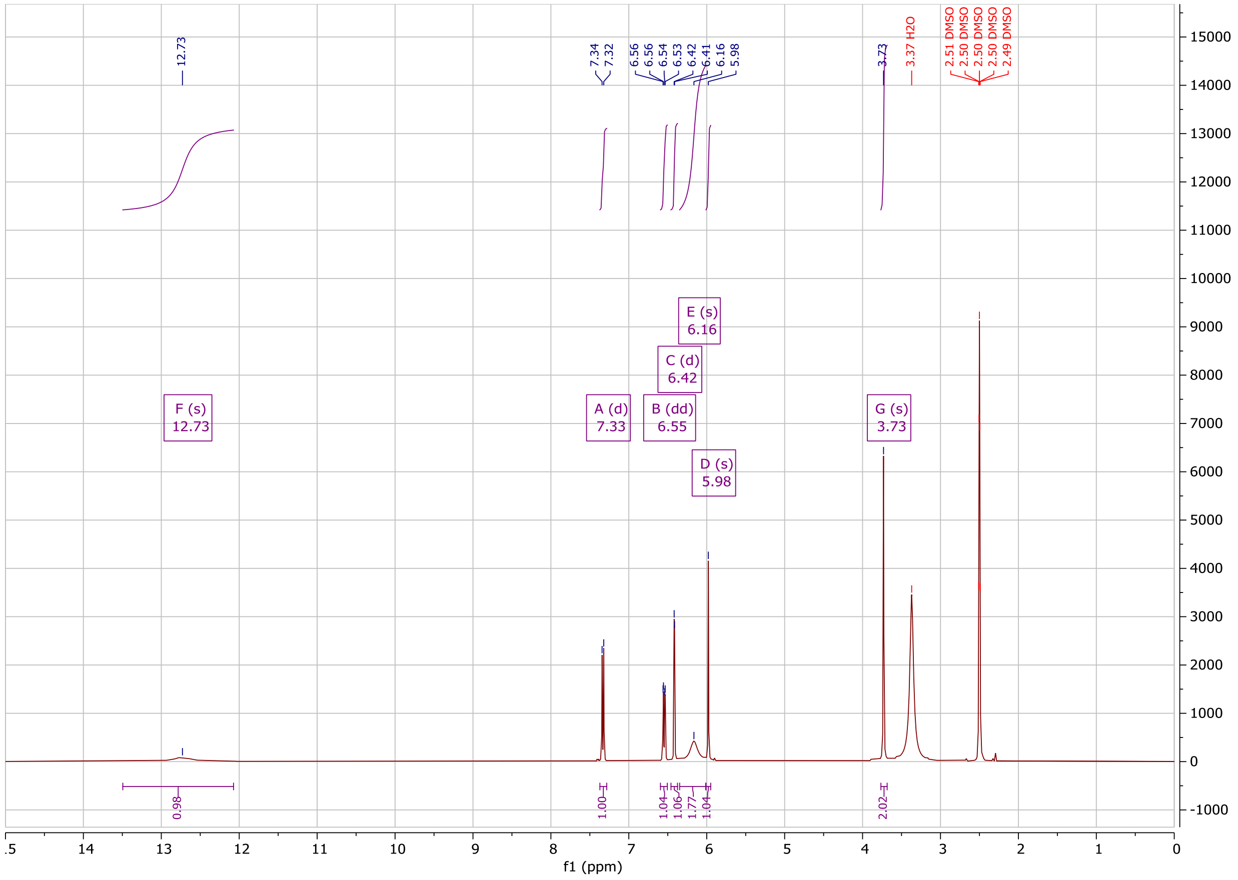

^{13}C-NMR ACC (23)

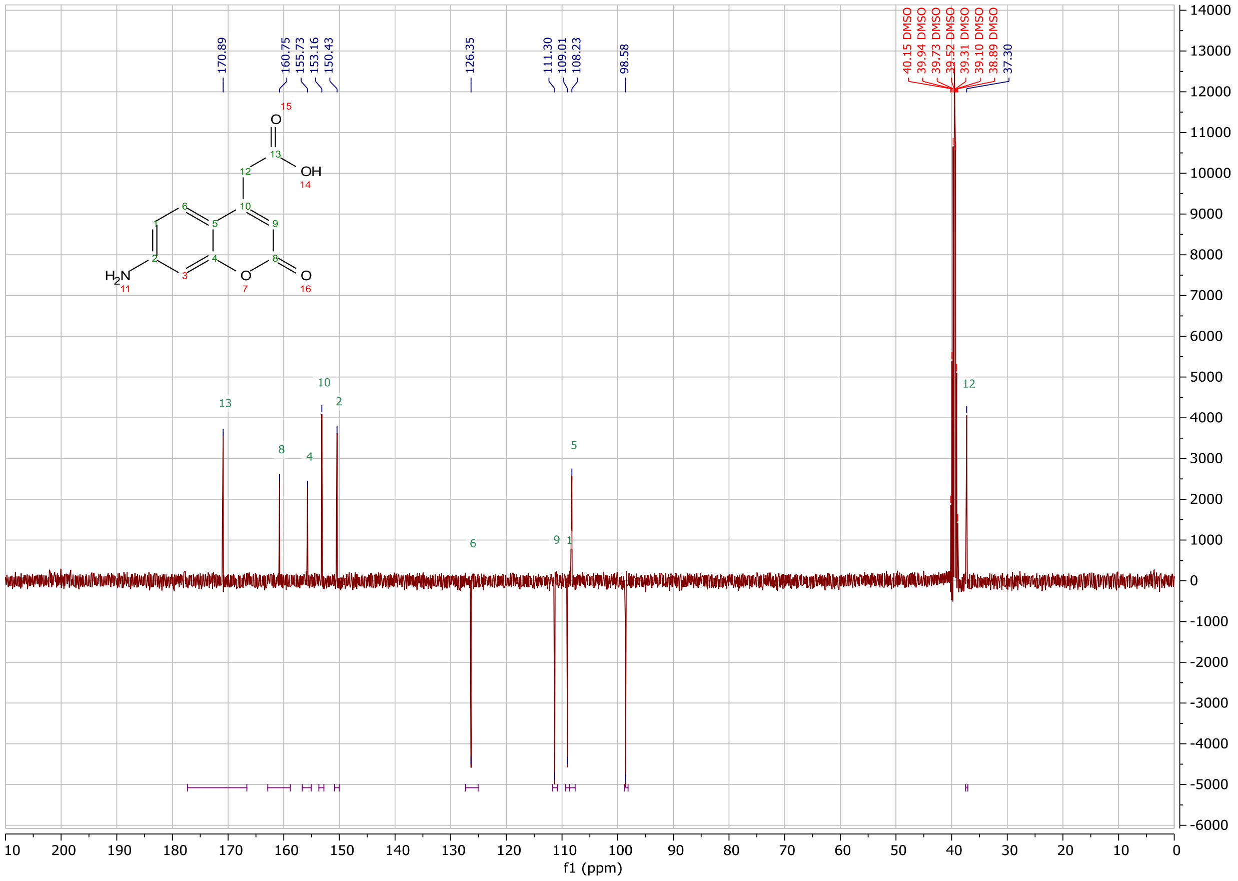

^{1}H-NMR AMC (15)

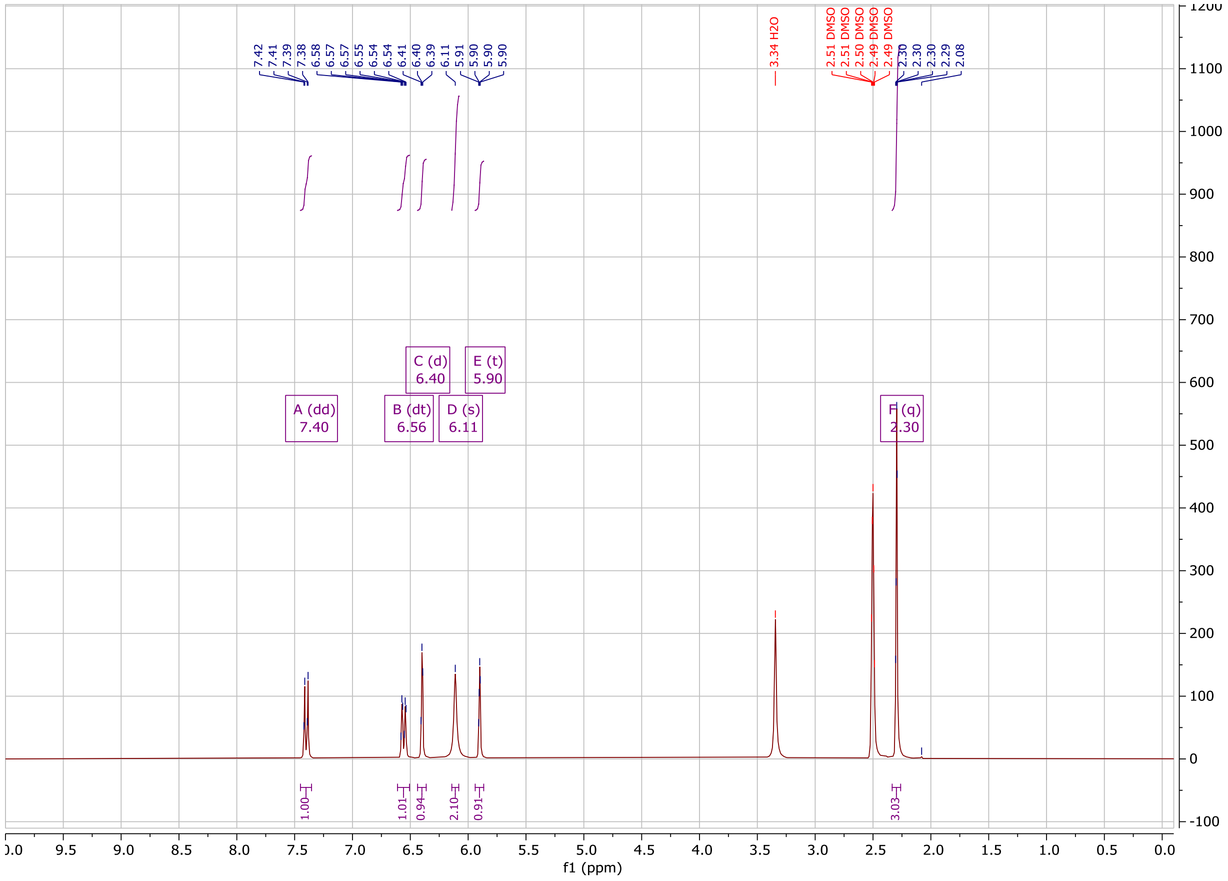

7.42
7.41
7.39
7.38
6.58
6.57
6.57
6.55
6.54
6.54
6.41
6.40
6.39
6.11
5.91
5.90
5.90
5.90
3.34 H2O
2.51 DMSO
2.51 DMSO
2.50 DMSO
2.49 DMSO
2.49 DMSO
2.30
2.30
2.30
2.29
2.08
A (dd) 7.40
B (dt) 6.56
C (d) 6.40
D (s) 6.11
E (t) 5.90
F (q) 2.30
1.00
1.01
0.94
2.10
0.91
3.03
f1 (ppm)

^{13}C-NMR AMC (15)

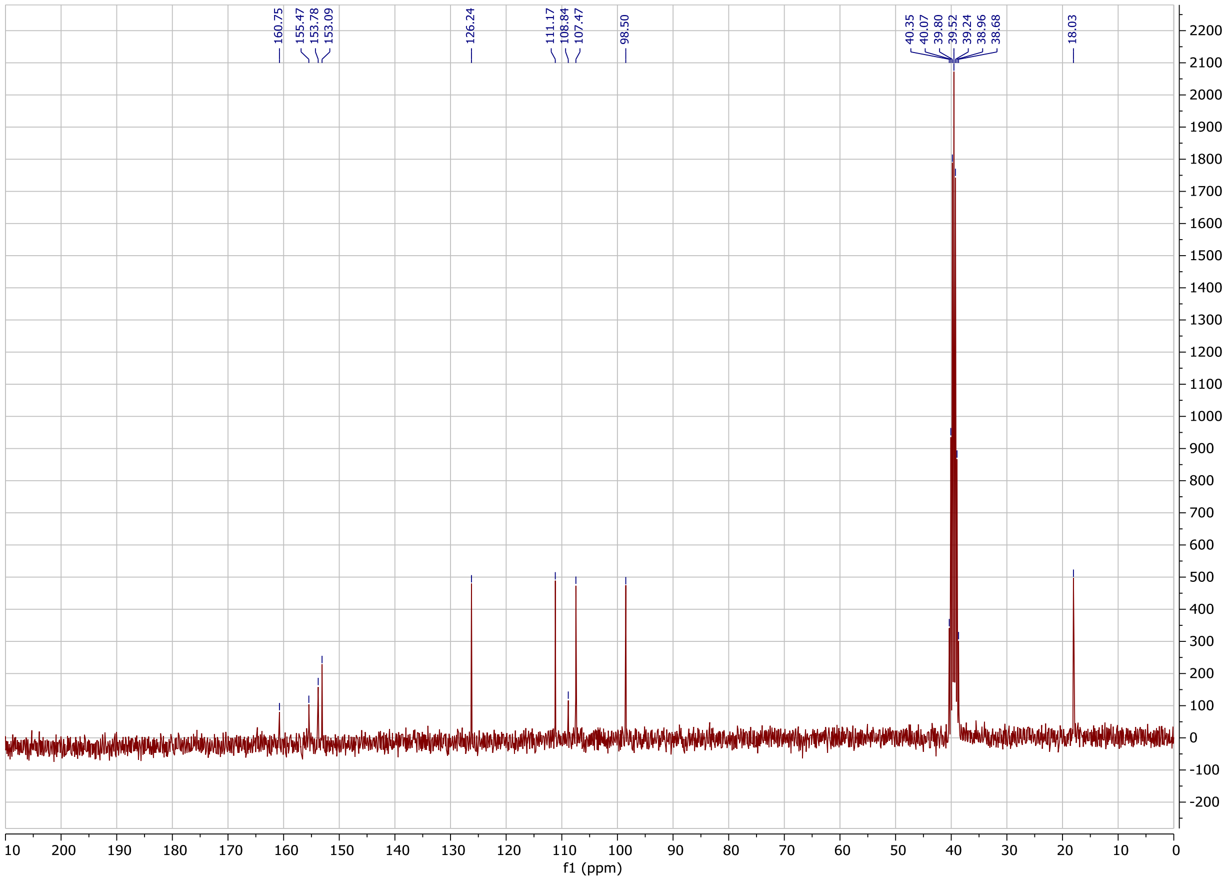

^{1}H-NMR LU-FS01i

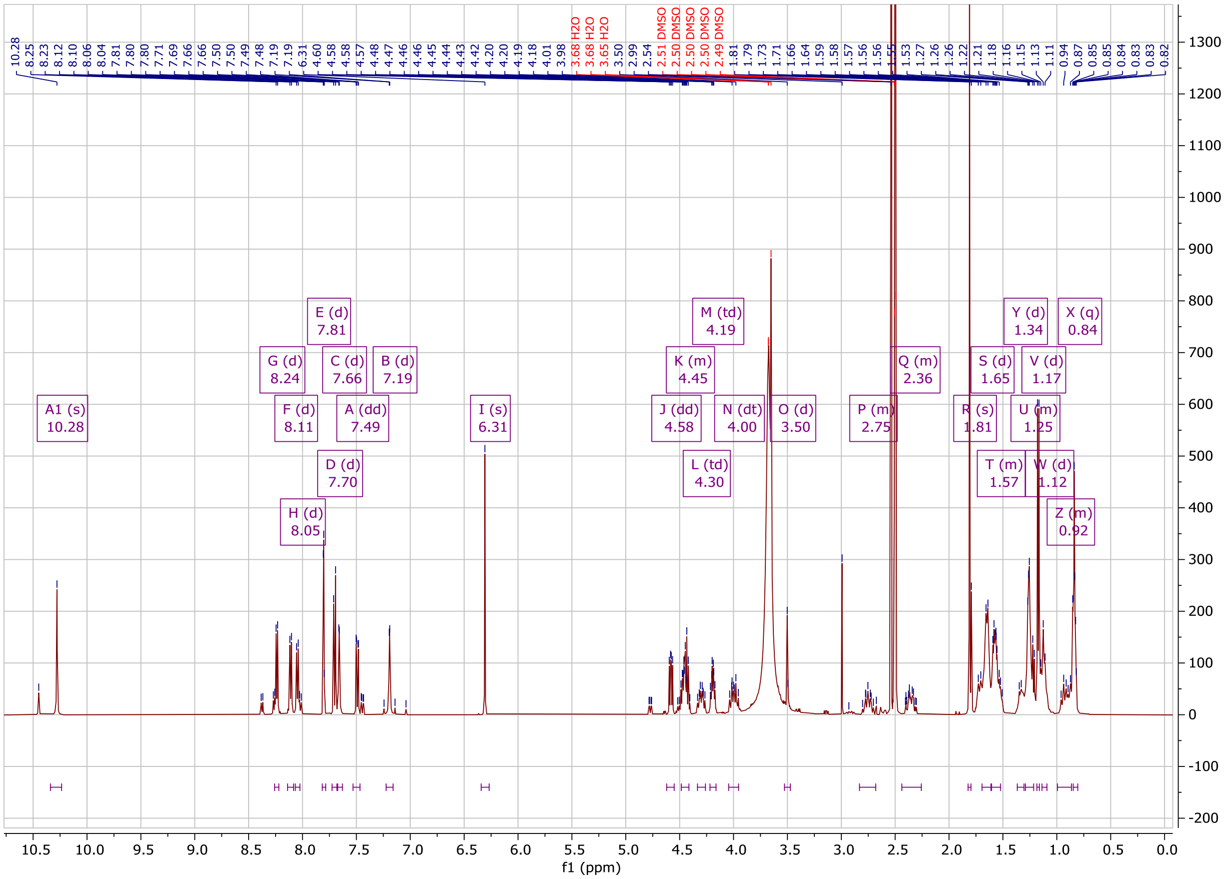

^{13}C-NMR LU-FS01i

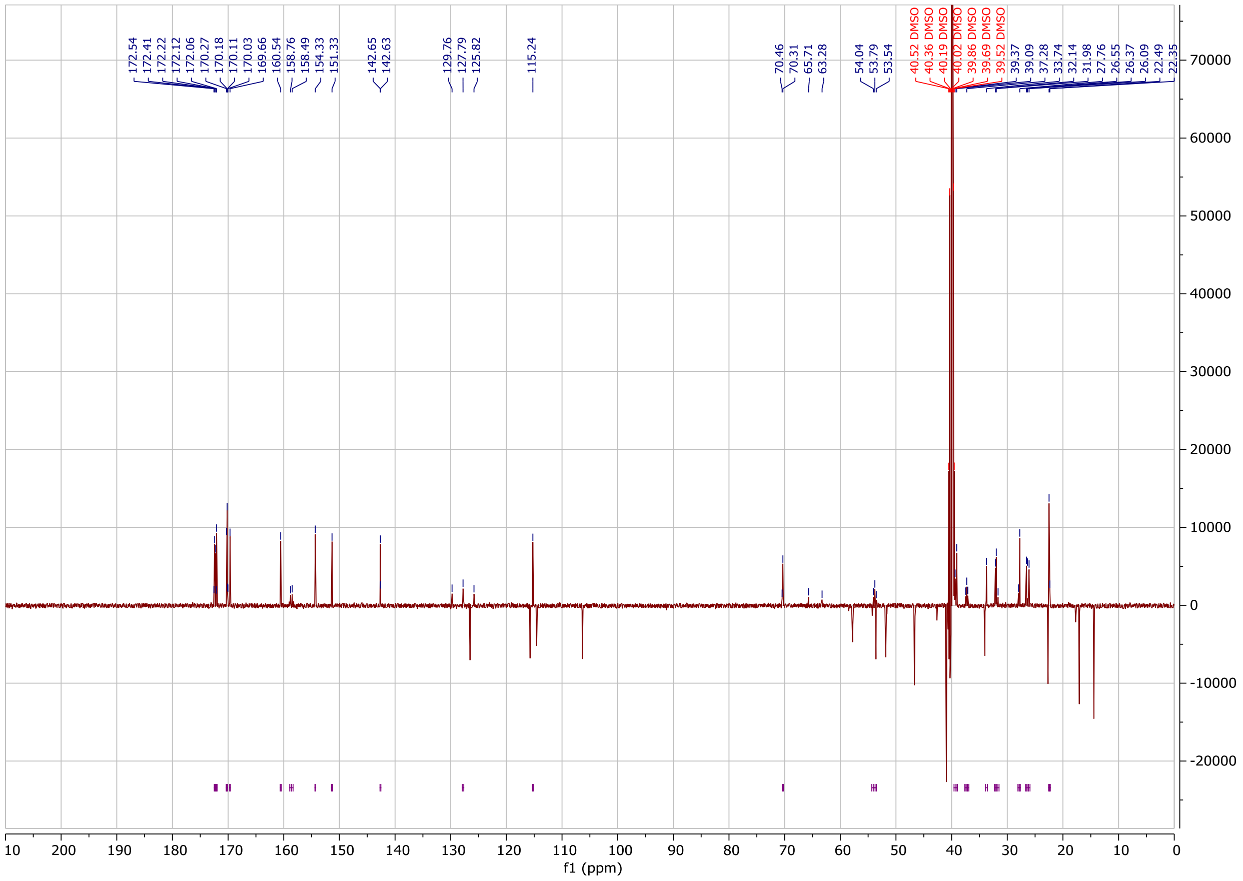

^{1}H-NMR LU-FS01c

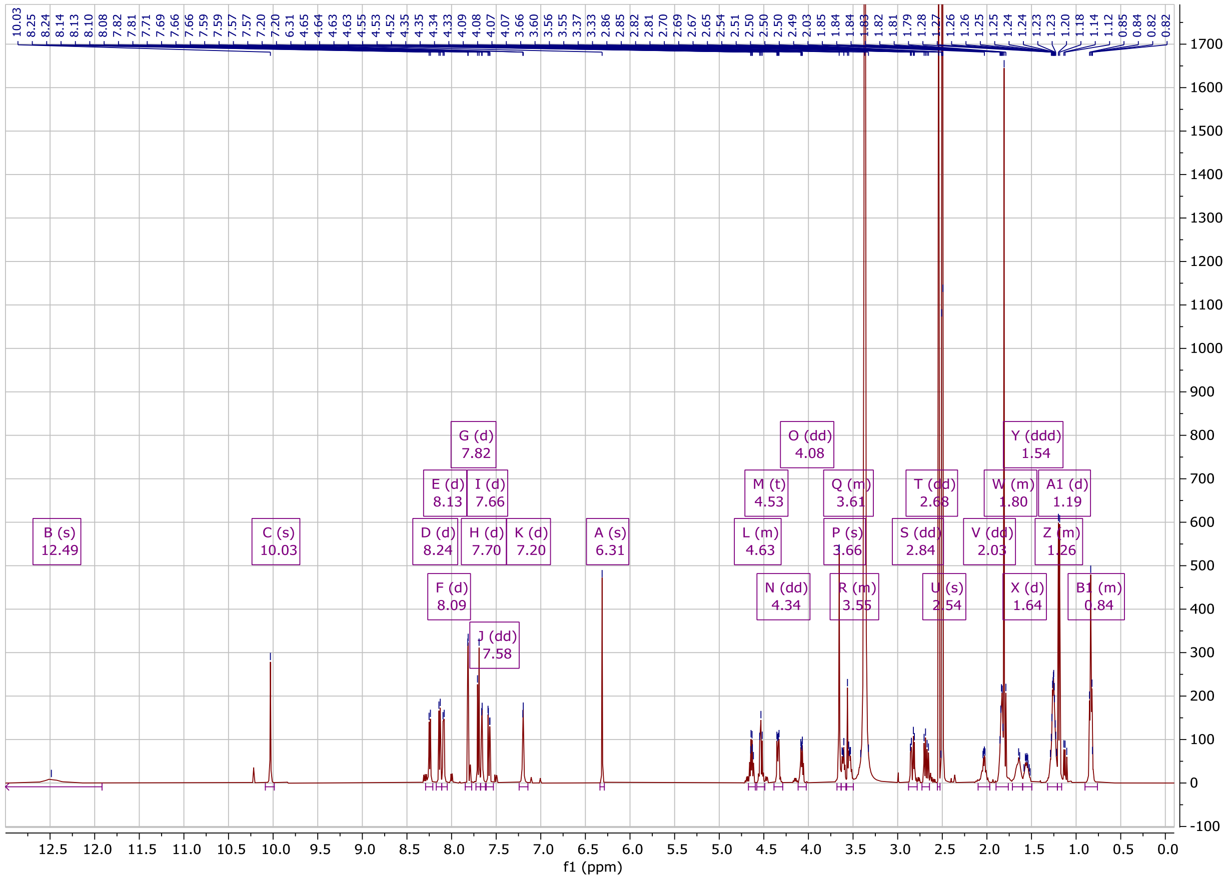

^{13}C-NMR LU-FS01c

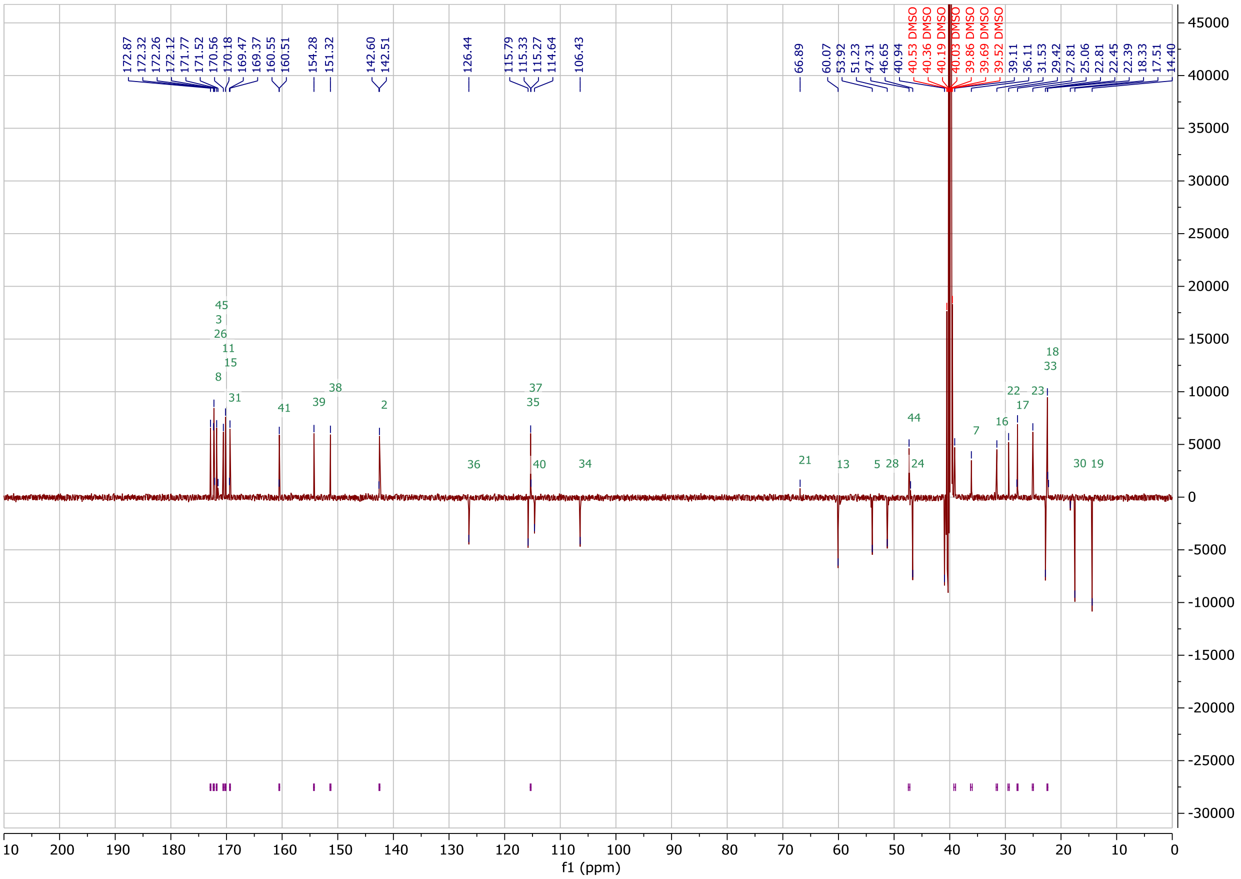

^{1}H-NMR LU-FS02i

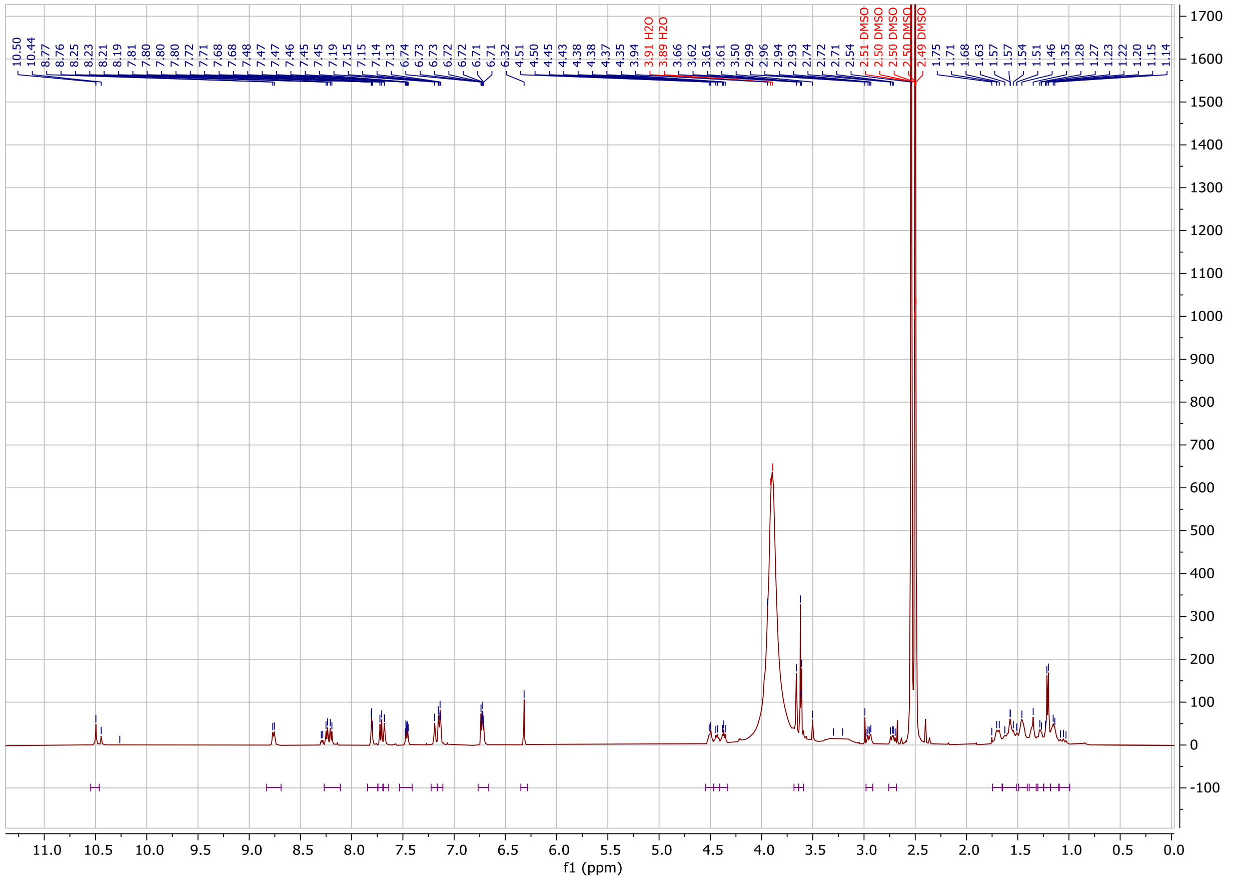

^{13}C-NMR LU-FS02i

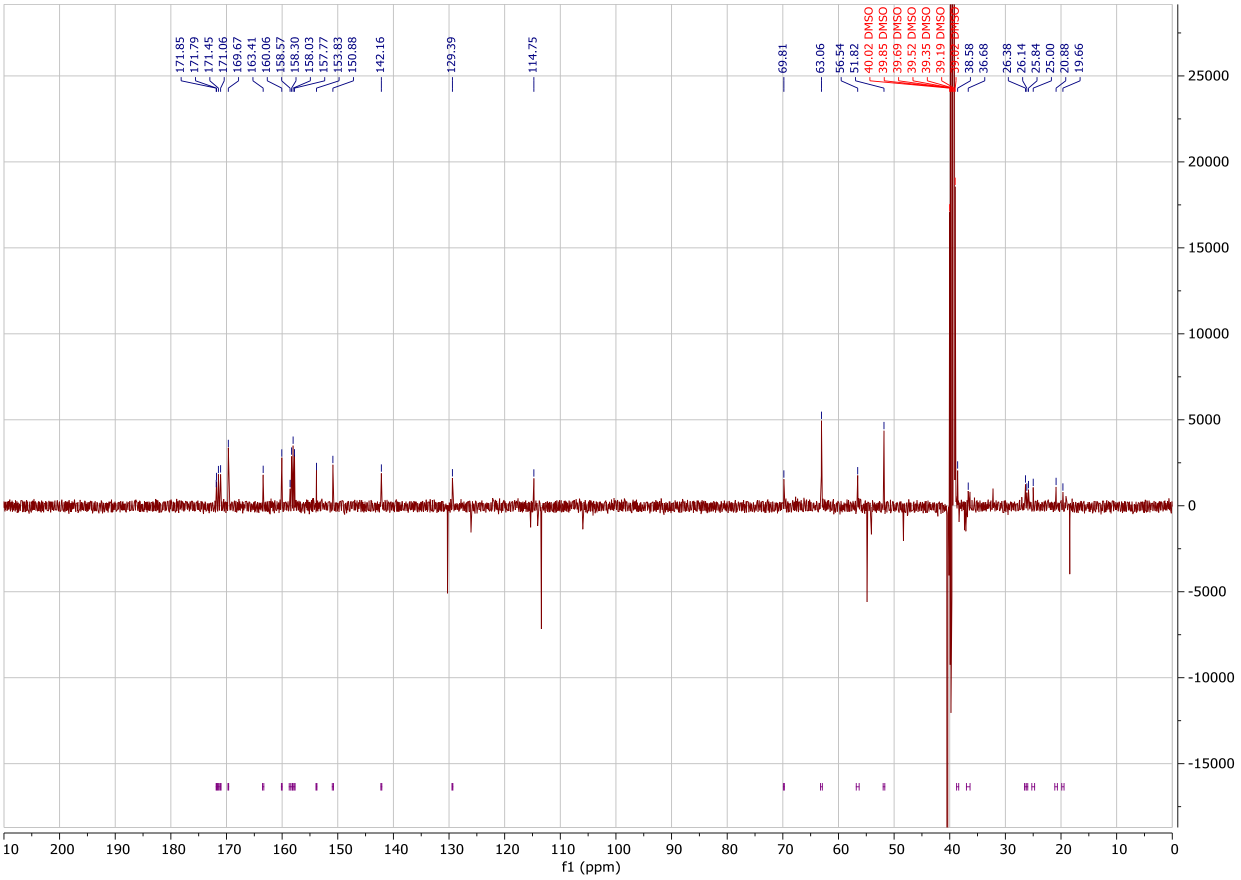

^{1}H-NMR LU-FS02c

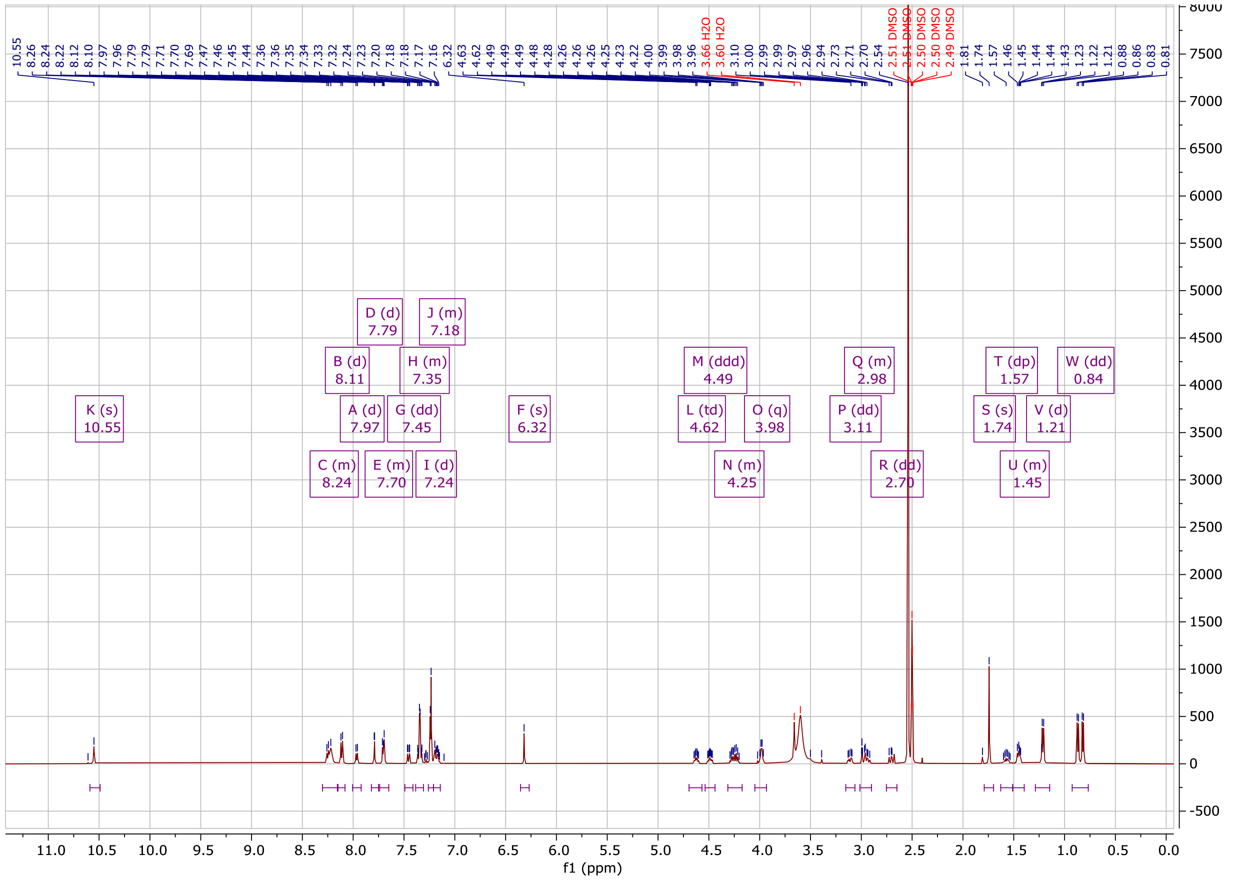

^{13}C-NMR LU-FS02c

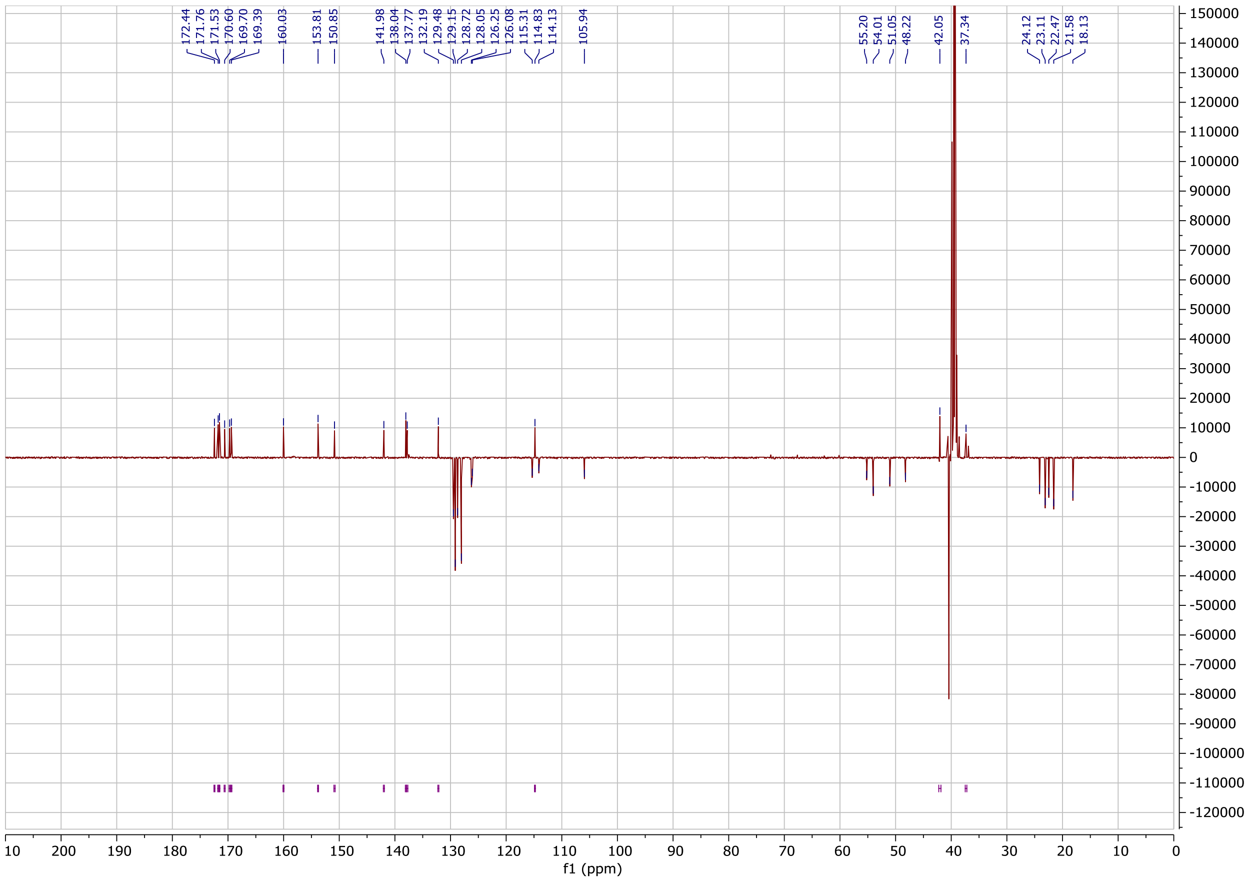

^{1}H-NMR LU-FS05i

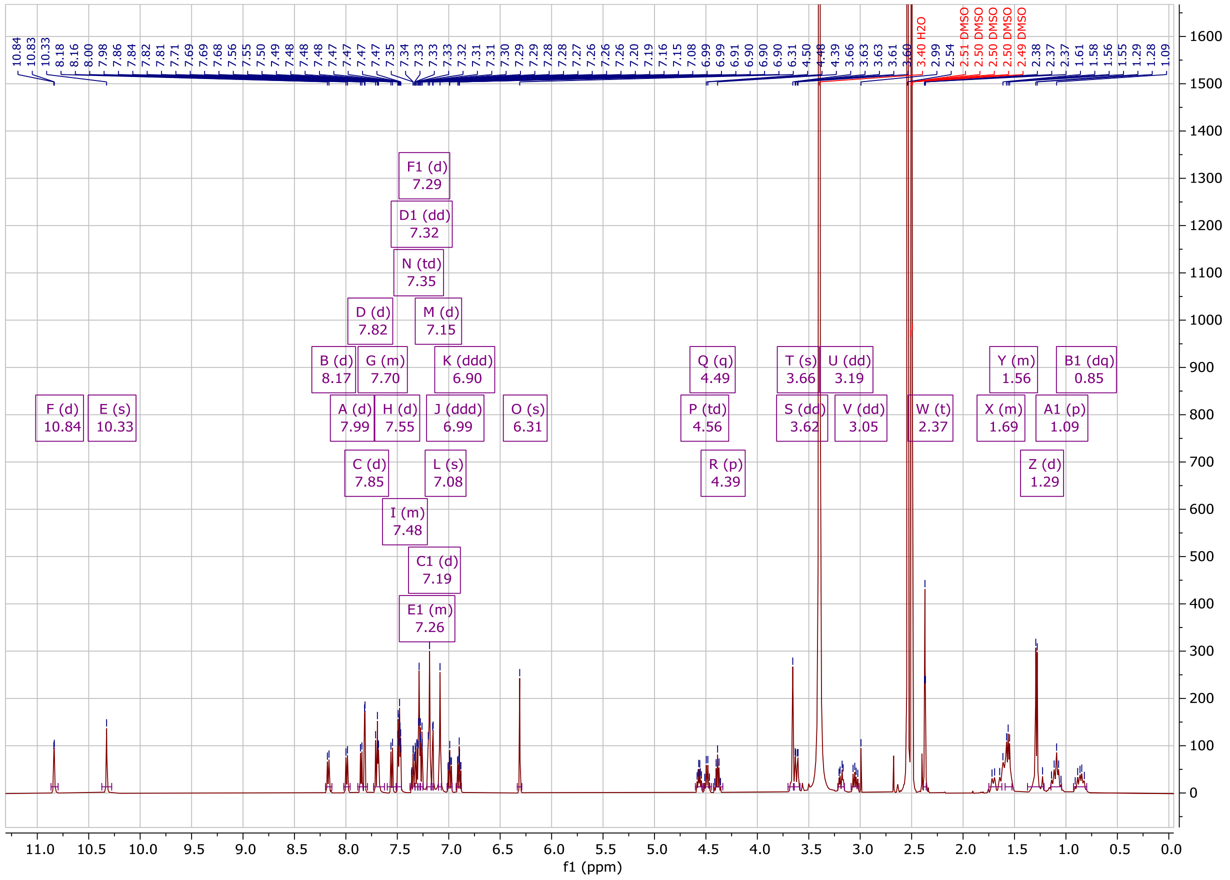

^{13}C-NMR LU-FS05i

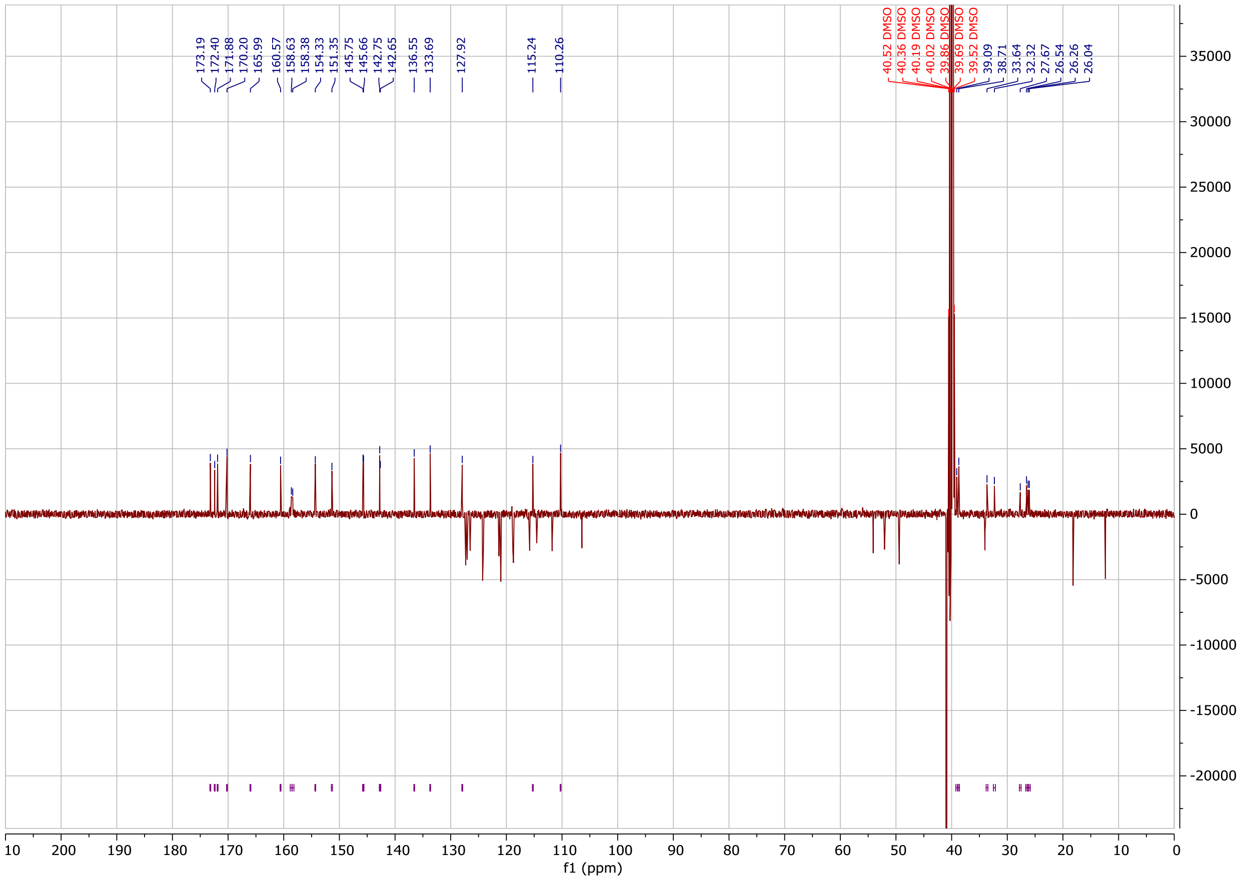

^{1}H-NMR LU-FS11c

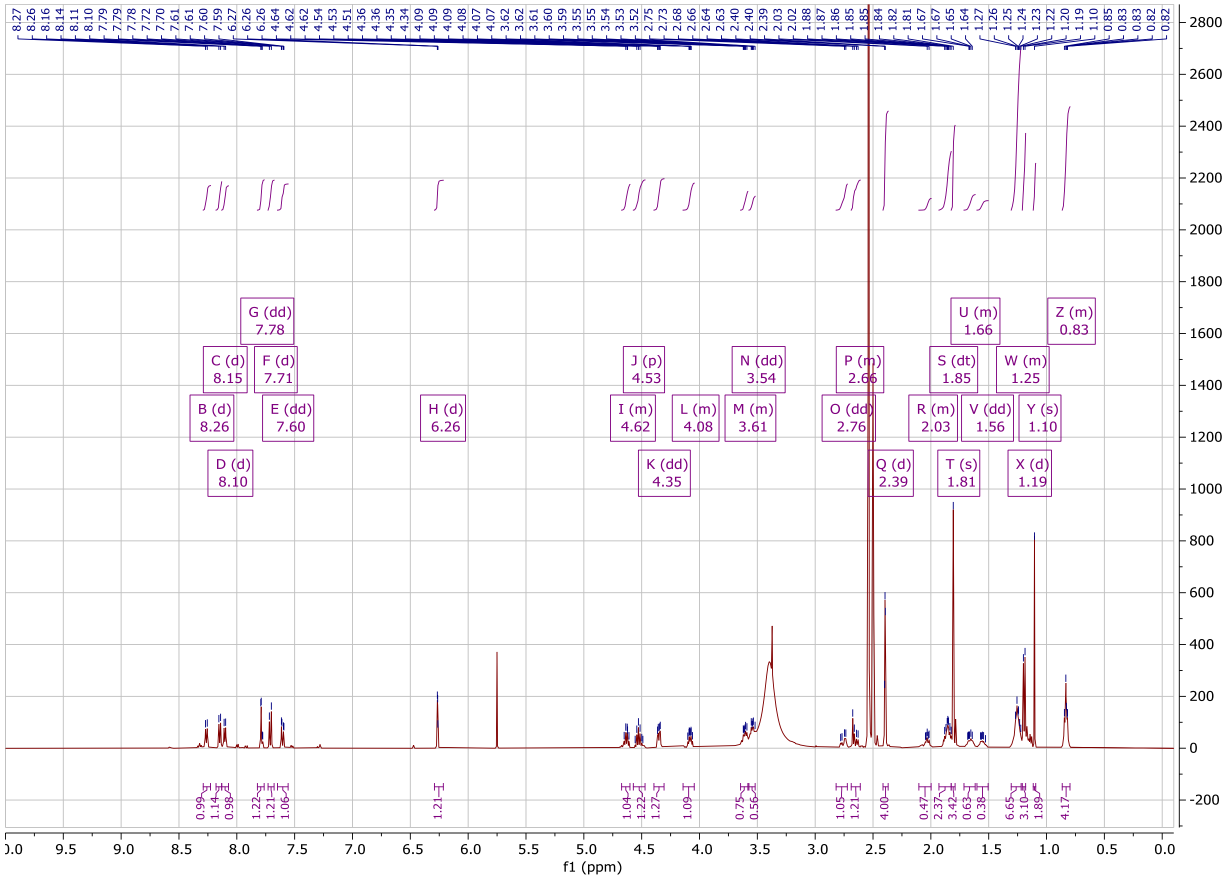

^{13}C-NMR LU-FS11c

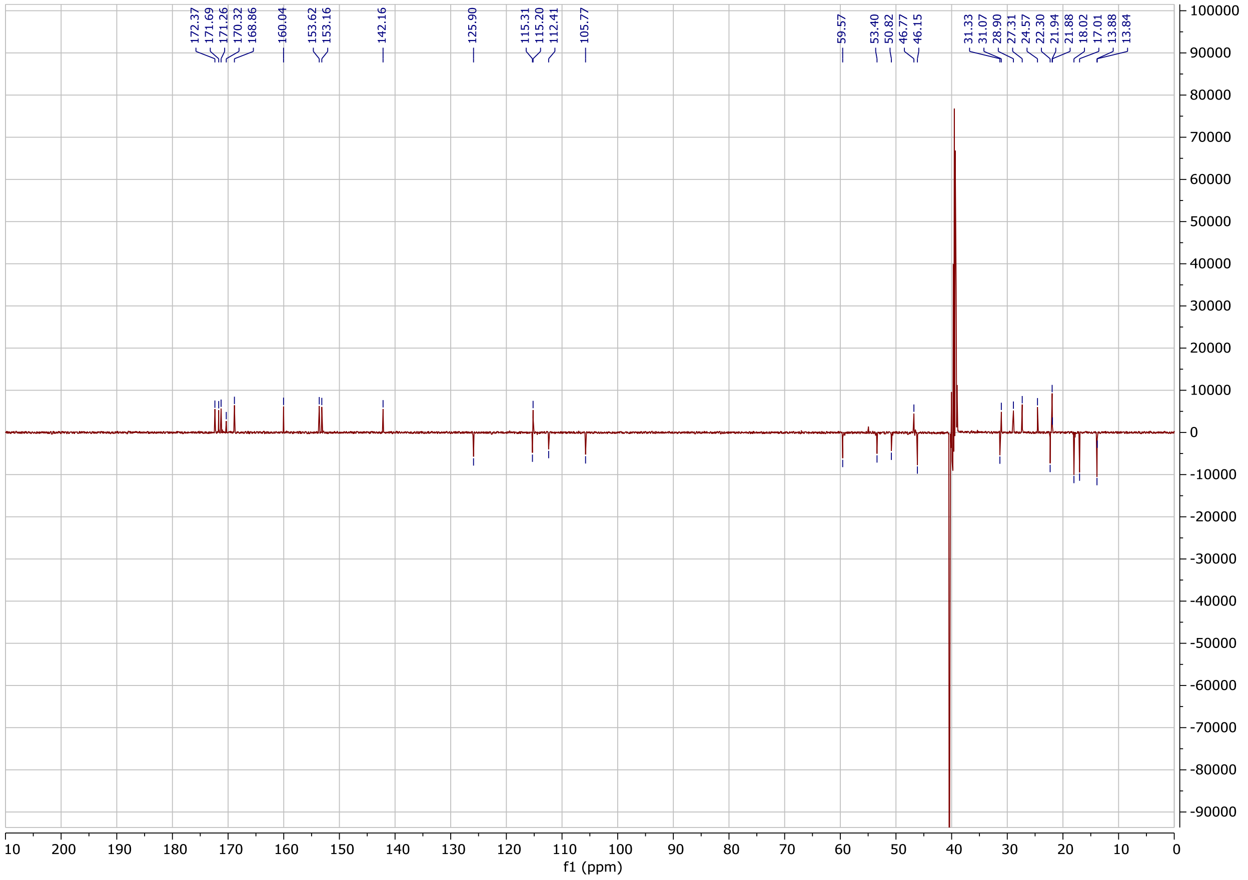

^{1}H-NMR LU-FS11i

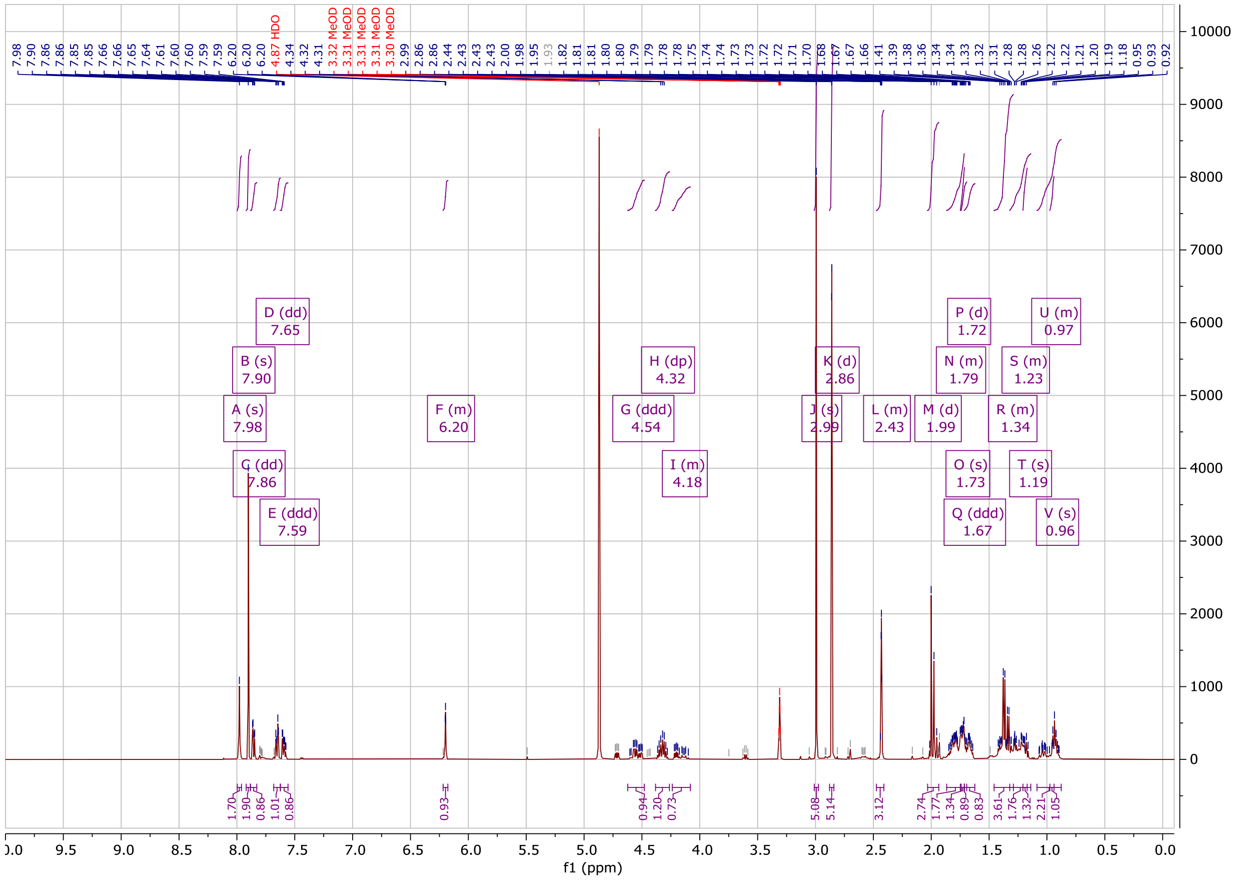

^{13}C-NMR LU-FS11i

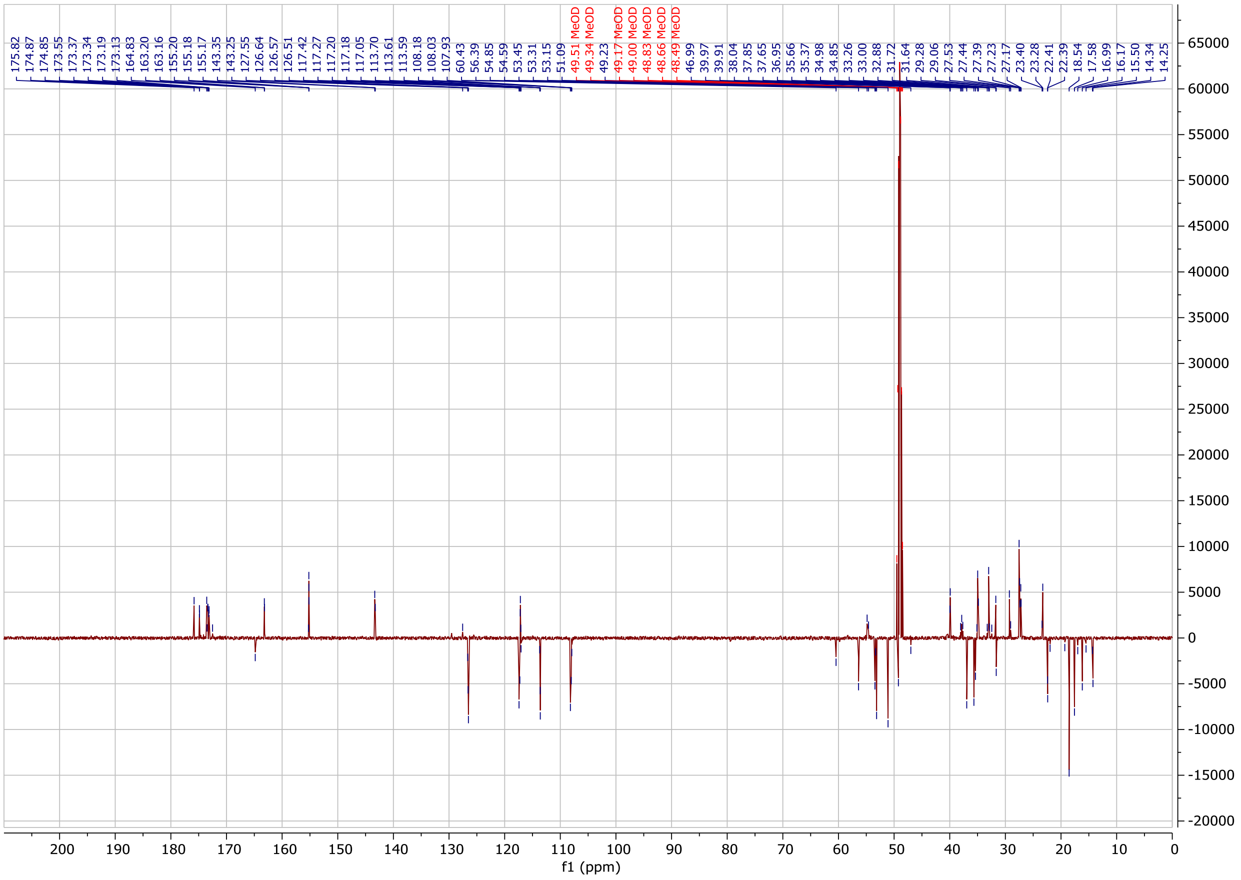

^{1}H-NMR LU-FS12c

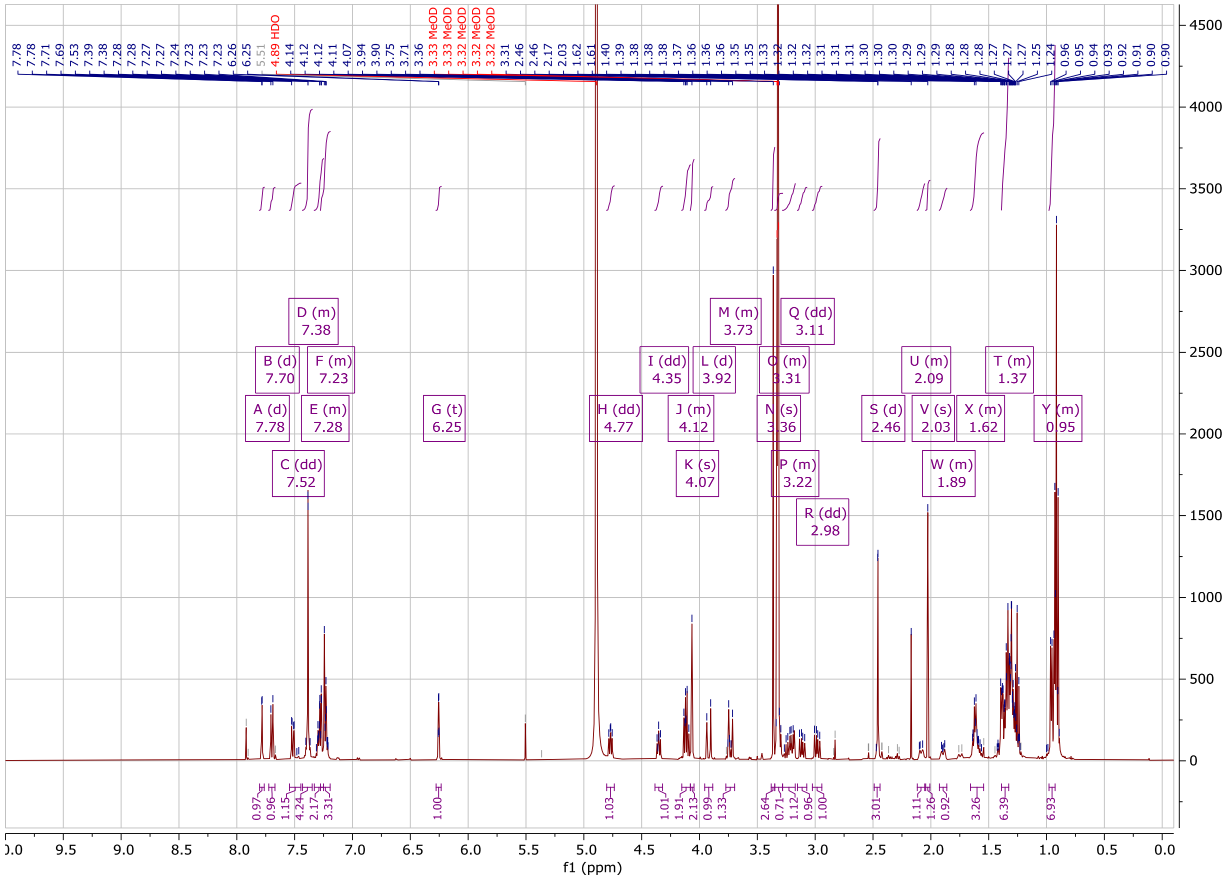

^{13}C-NMR LU-FS12c

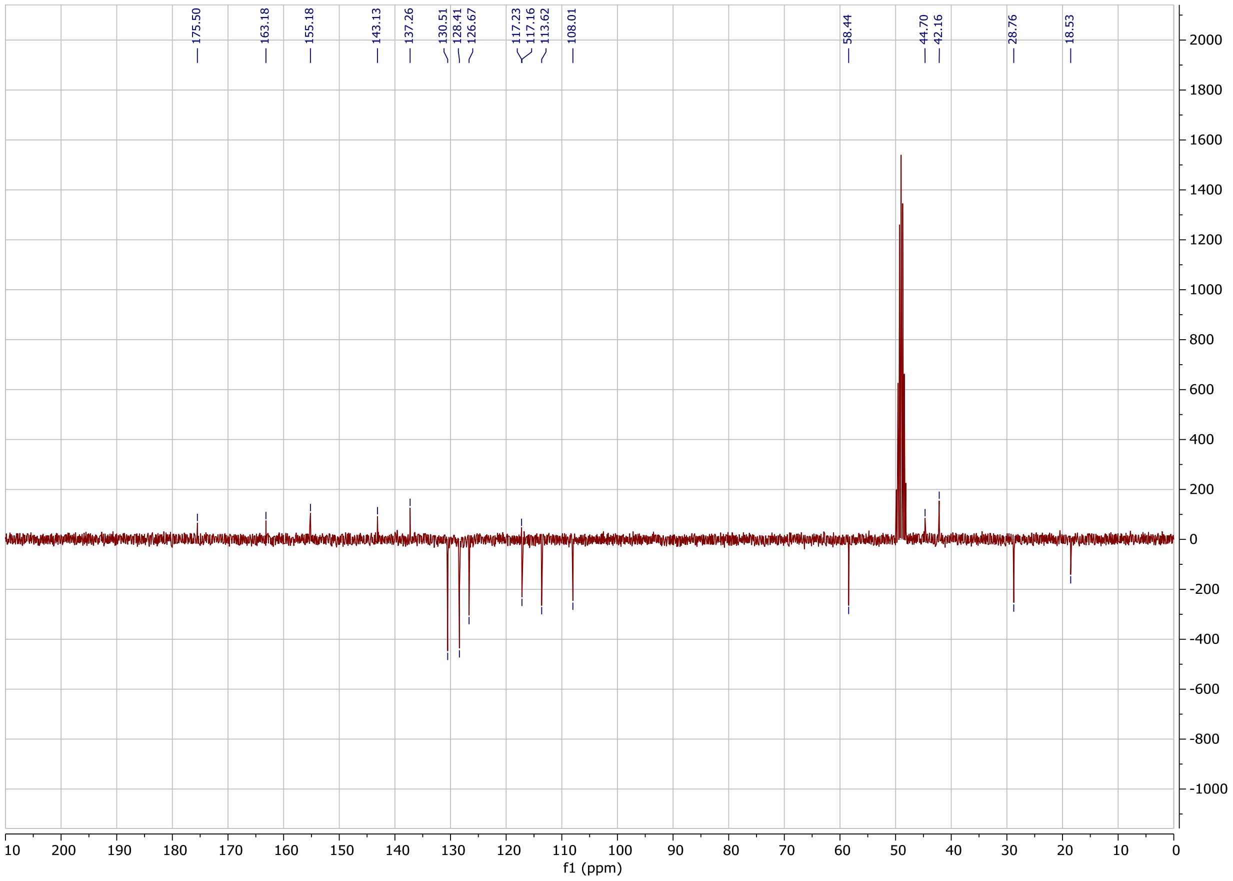

^{1}H-NMR LU-FS12i

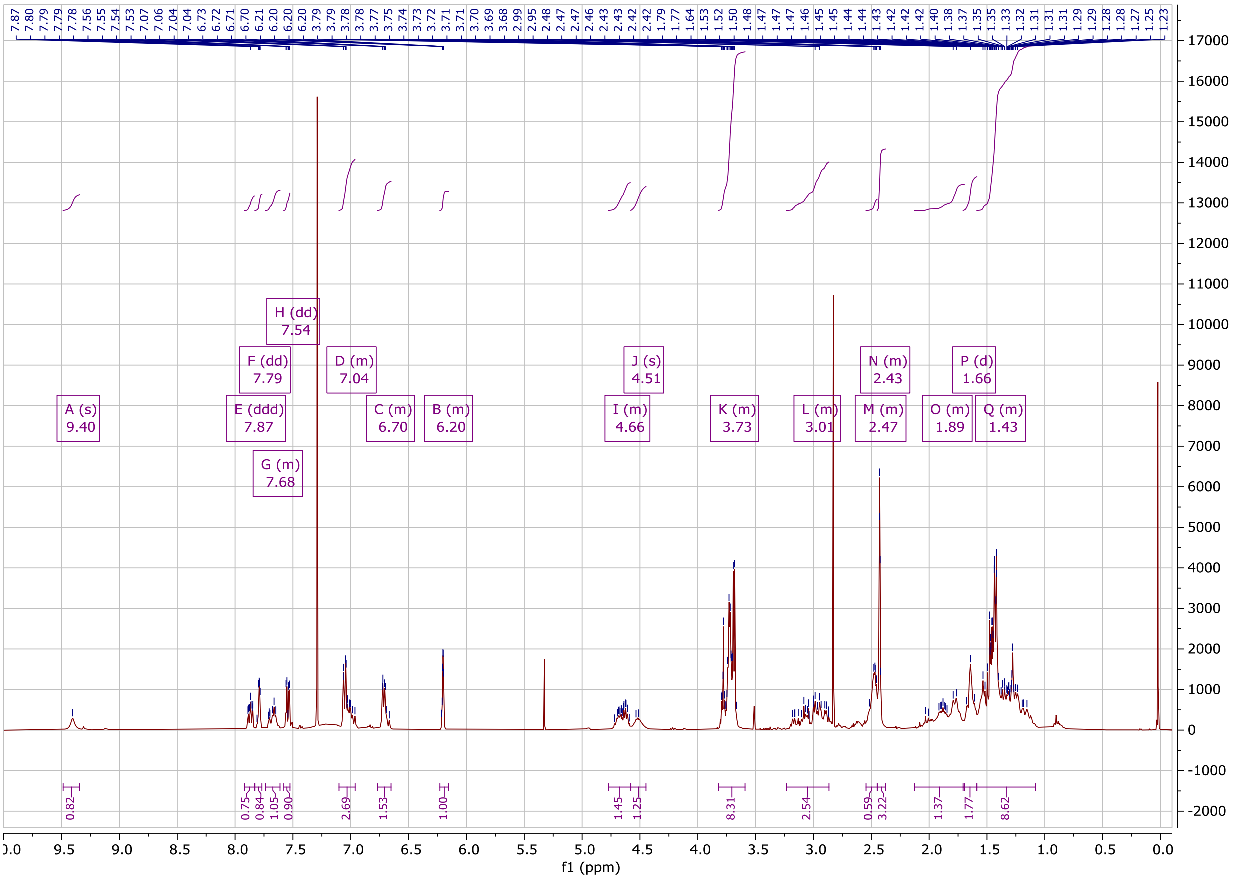

^{13}C-NMR LU-FS12i

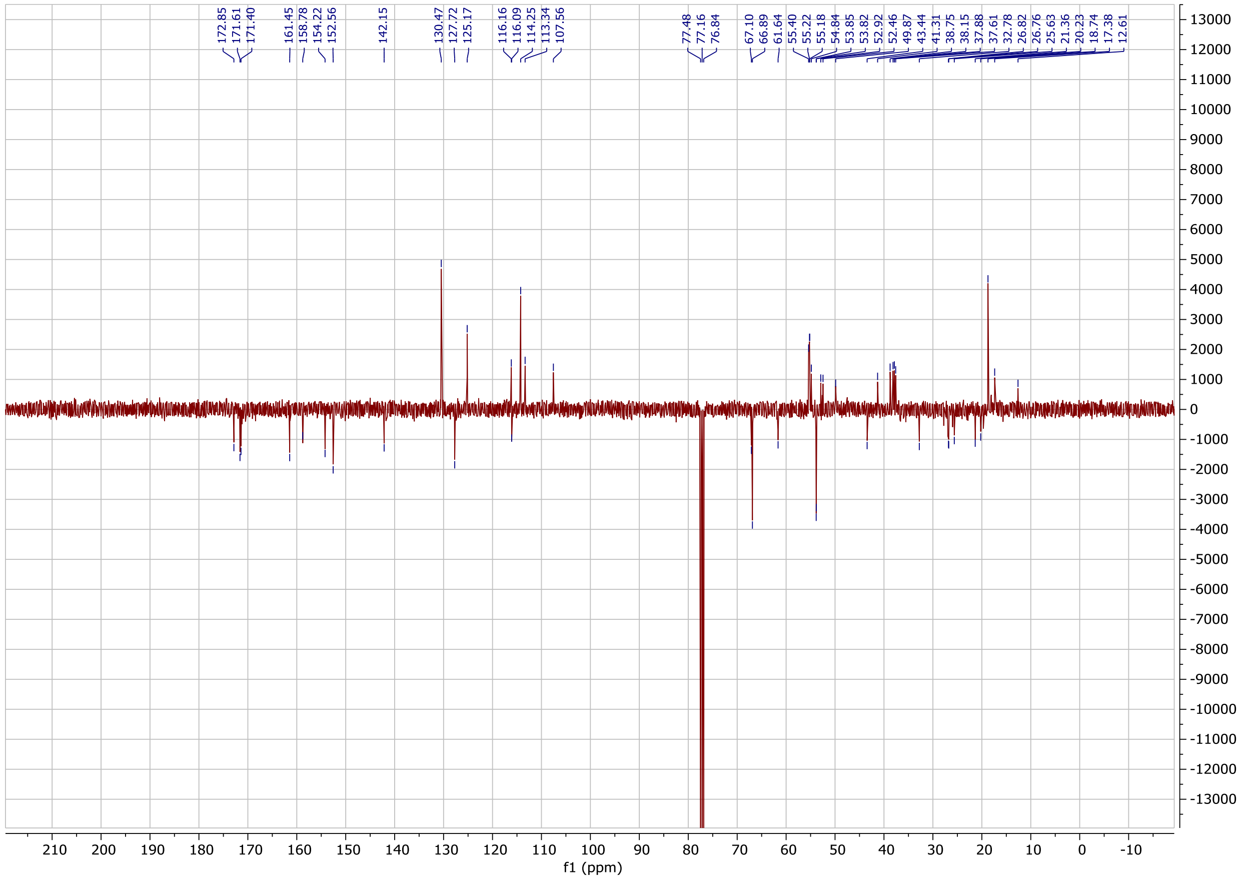

^{1}H-NMR LU-FS15c

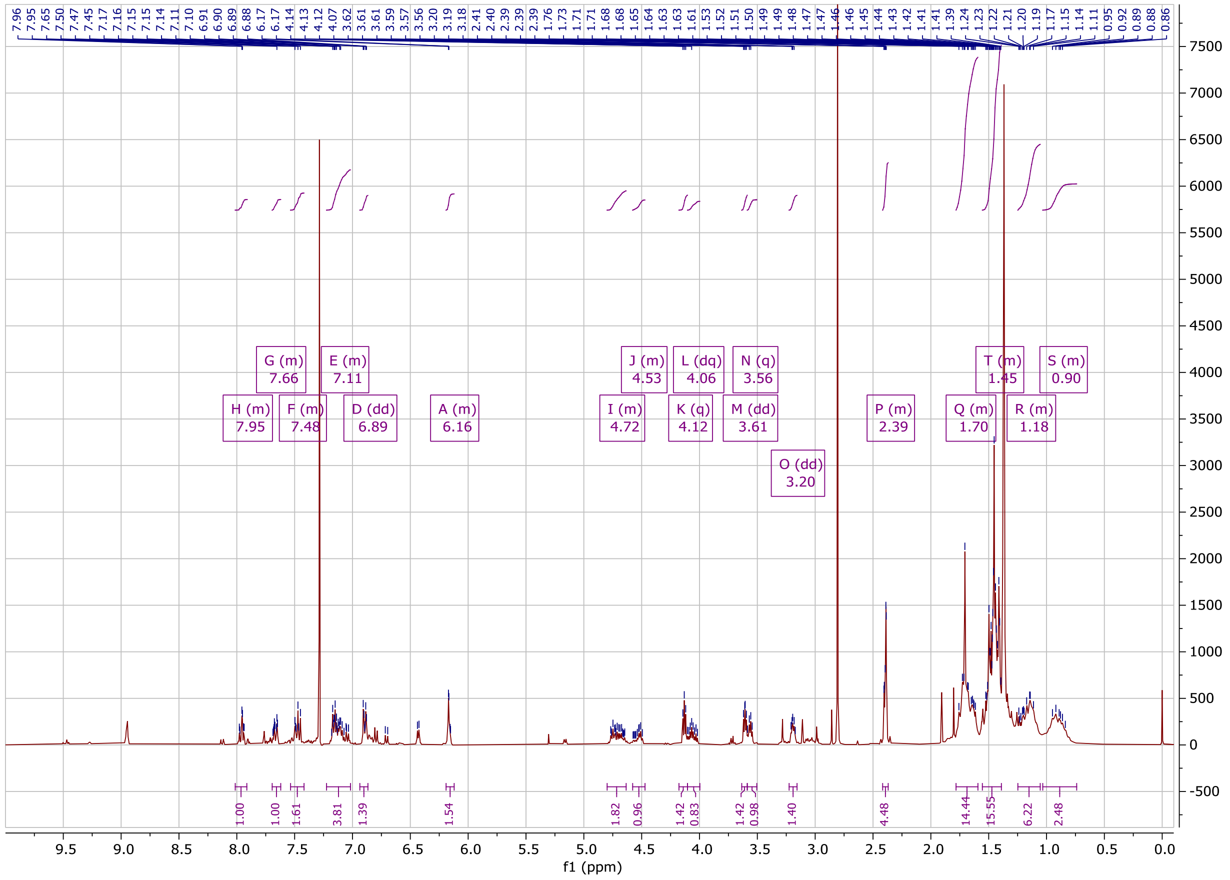

13C-NMR LU-FS15c

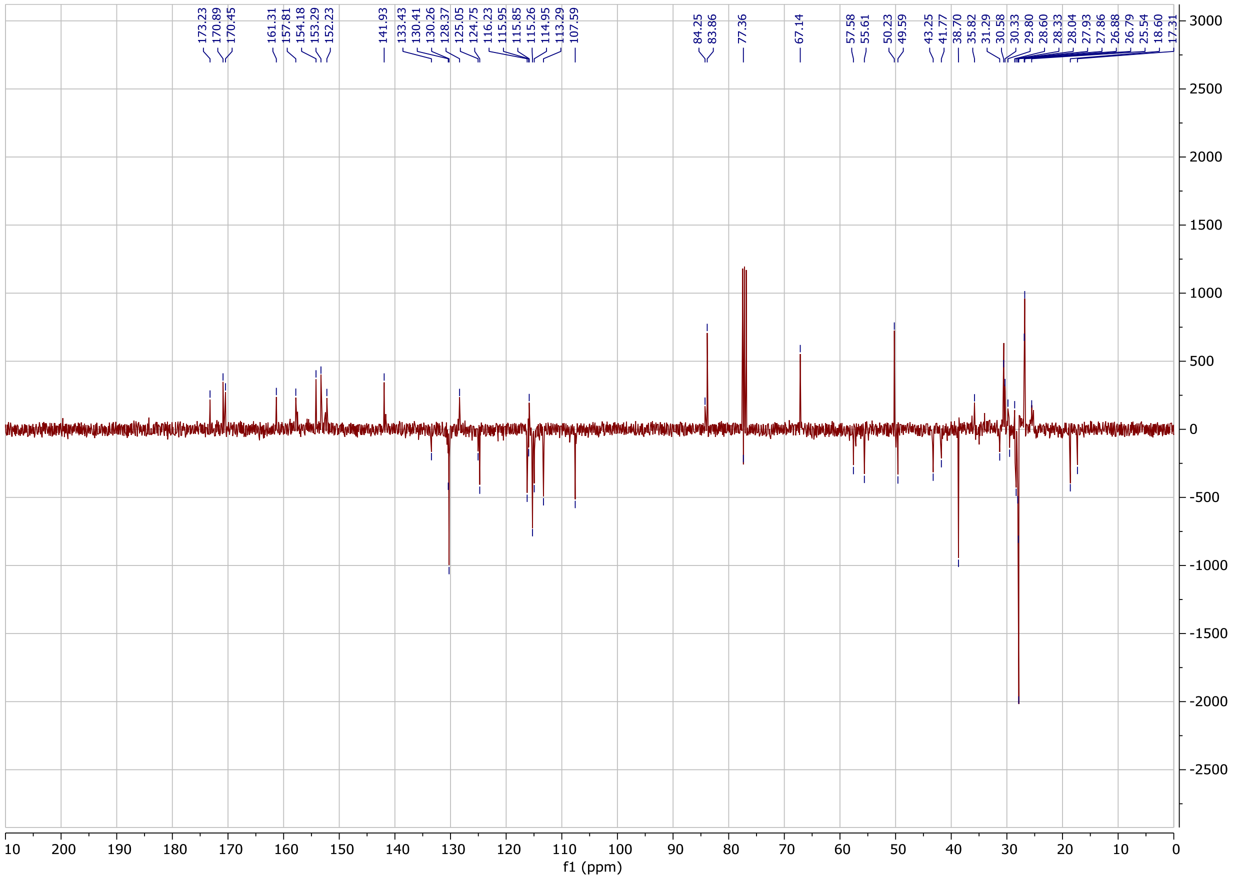

HRMS Data

HRMS LU-FS01c

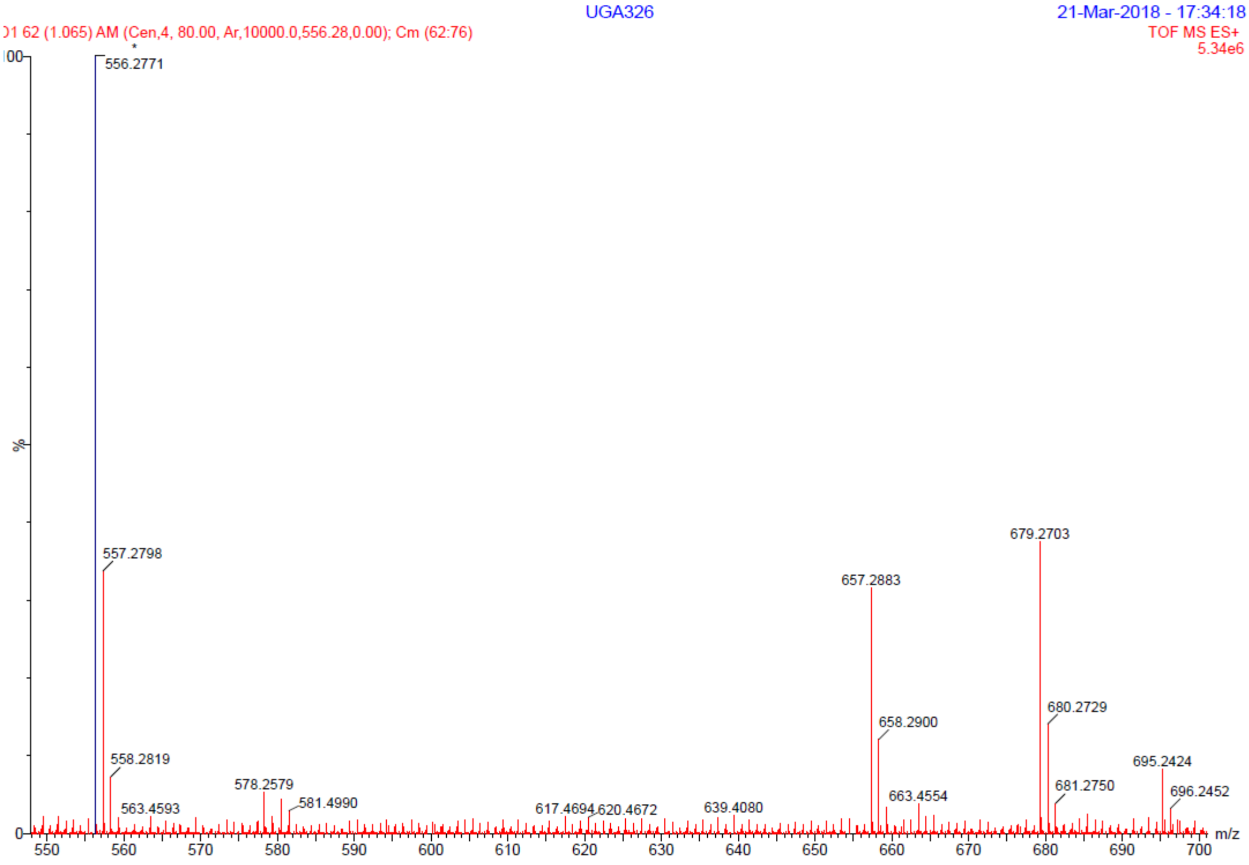

HRMS LU-FS01i

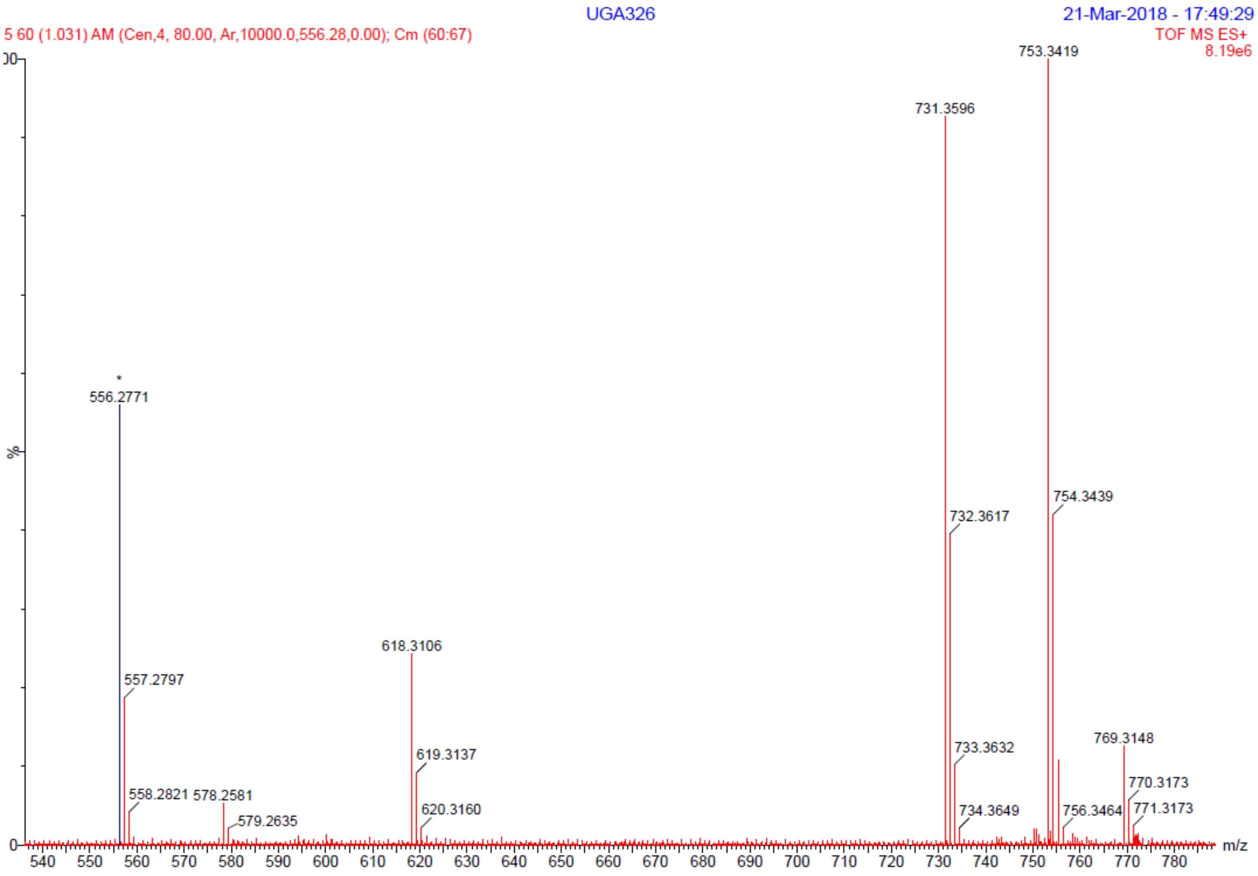

HRMS LU-FS02c

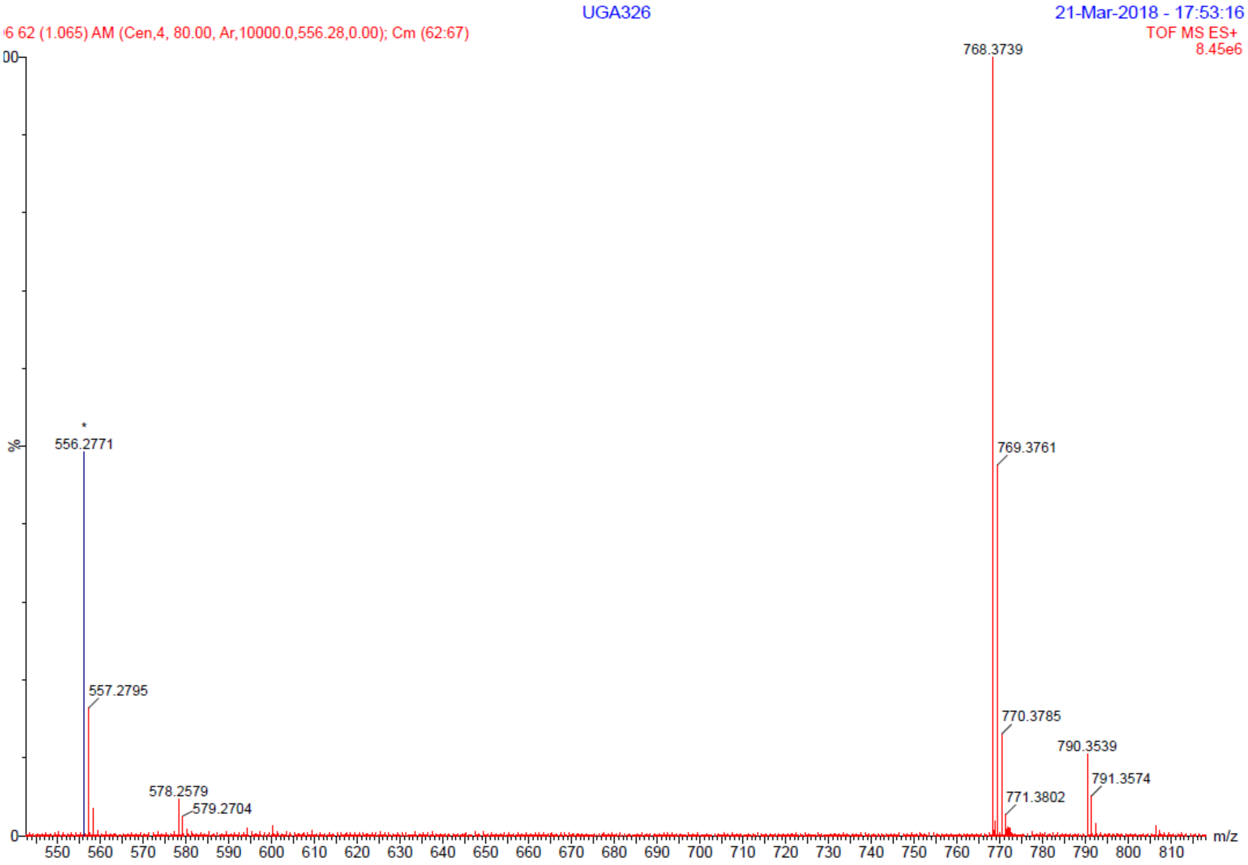

HRMS LU-FS02i

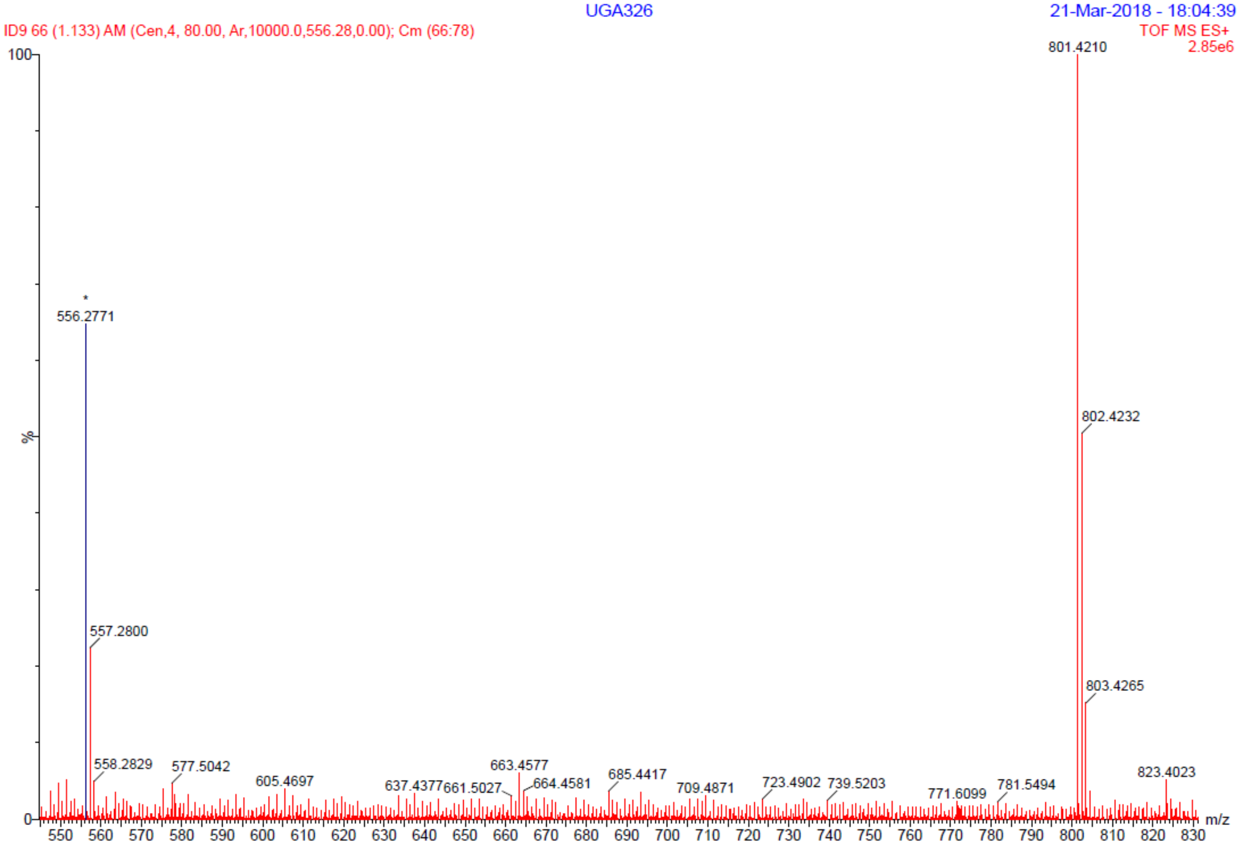

HRMS LU-FS05c

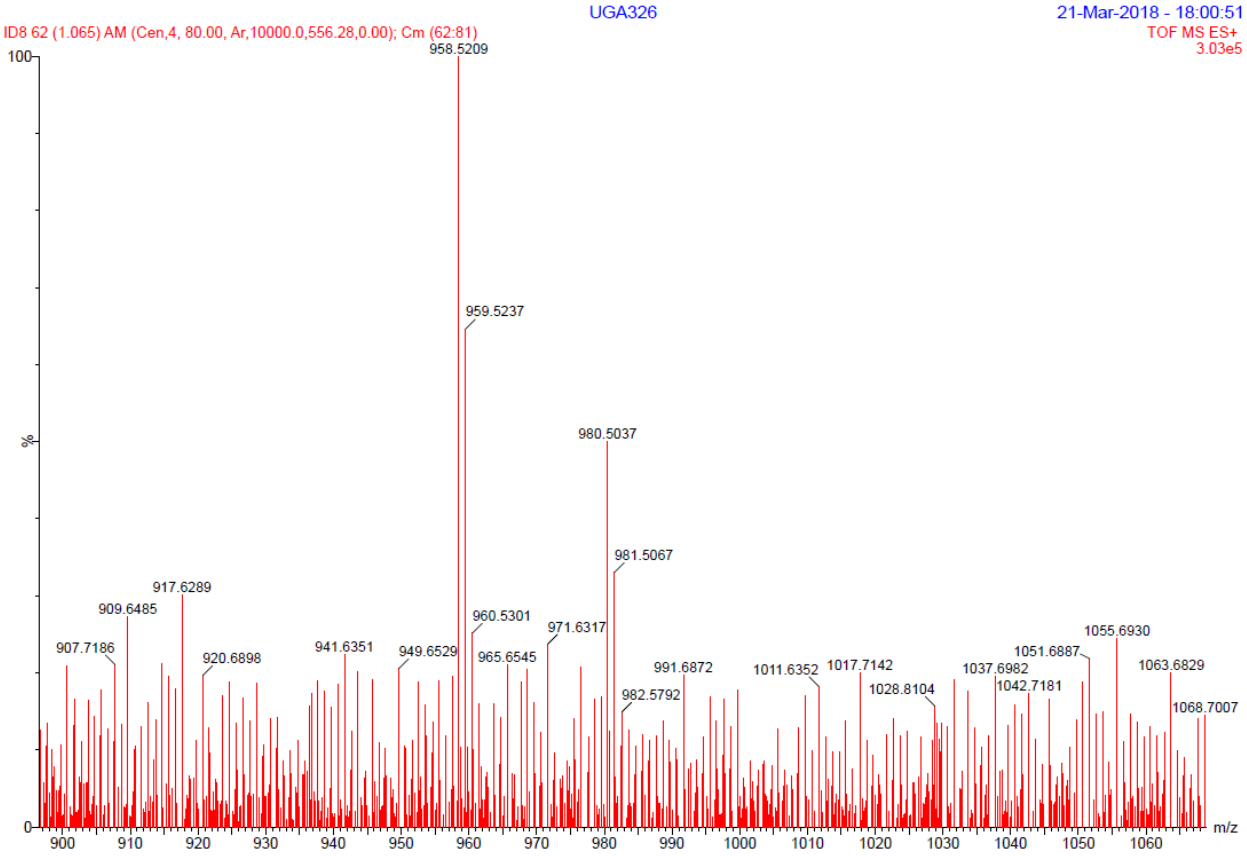

HRMS LU-FS05i

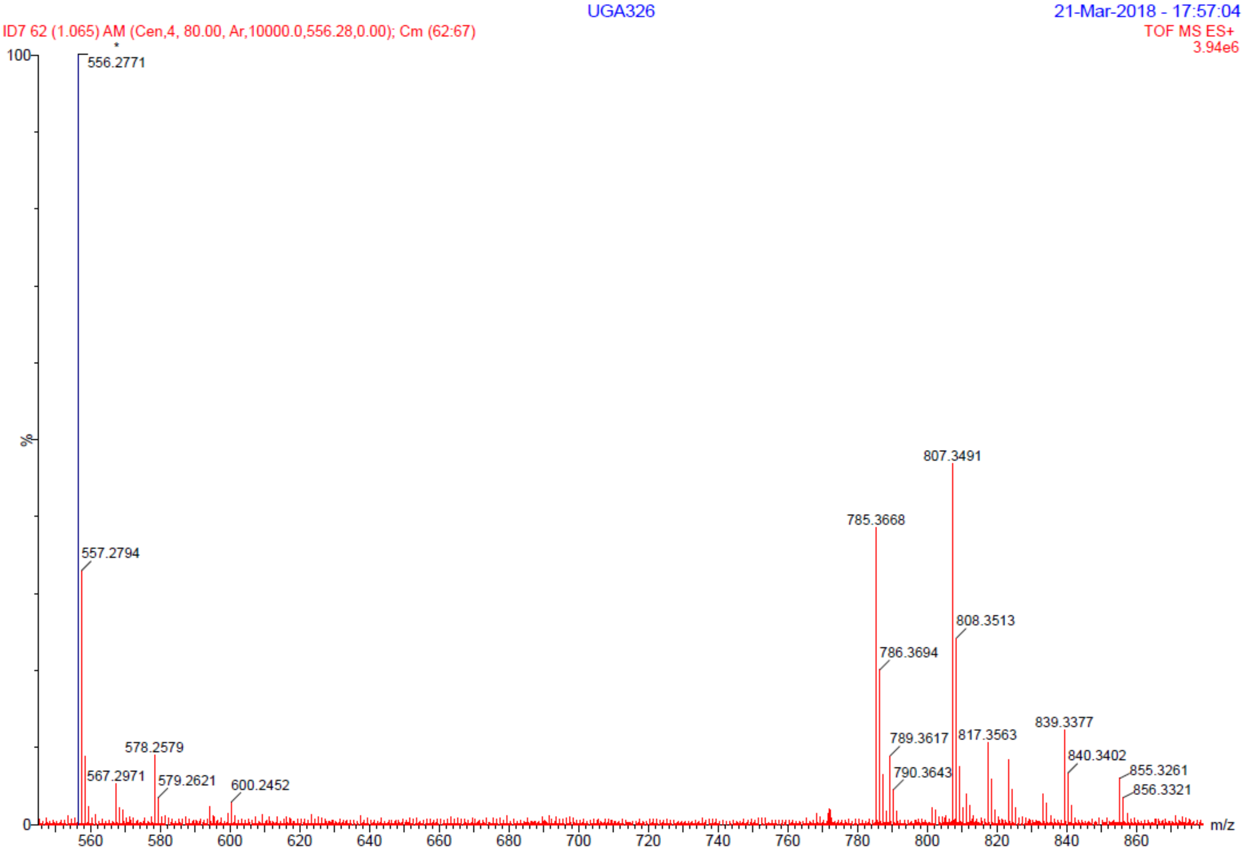

Supplement: Supplementary file 1 — Supplementary [file CBIC-21-3220-s001.pdf]
